# High-throughput functional mapping of variants in an arrhythmia gene, *KCNE1*, reveals novel biology

**DOI:** 10.1186/s13073-024-01340-5

**Published:** 2024-05-30

**Authors:** Ayesha Muhammad, Maria E. Calandranis, Bian Li, Tao Yang, Daniel J. Blackwell, M. Lorena Harvey, Jeremy E. Smith, Zerubabell A. Daniel, Ashli E. Chew, John A. Capra, Kenneth A. Matreyek, Douglas M. Fowler, Dan M. Roden, Andrew M. Glazer

**Affiliations:** 1 Vanderbilt Genetics Institute, Vanderbilt University Medical Center, 1235 Medical Research Building IV, 2215B Garland Avenue, Nashville, TN 37232 USA; 2 Medical Scientist Training Program, Vanderbilt University, Nashville, TN 37232 USA; 3 Department of Medicine, Vanderbilt University Medical Center, Nashville, TN 37232 USA; 4 Regeneron Pharmaceuticals Inc., Tarrytown, NY USA; 5 Bakar Computational Health Sciences Institute and Department of Epidemiology and Biostatistics, University of California, San Francisco, CA 94143 USA; 6 Department of Pathology, Case Western Reserve University School of Medicine, Cleveland, OH 44106 USA; 7 Department of Genome Sciences, University of Washington, Seattle, WA 98195 USA; 8 Department of Biomedical Informatics, Vanderbilt University Medical Center, Nashville, TN 37232 USA; 9 Department of Pharmacology, Vanderbilt University Medical Center, Nashville, TN 37232 USA

**Keywords:** Multiplexed assay of variant effect, Long QT syndrome, KCNE1, Ion channel, Arrhythmia, Saturation mutagenesis, Variant classification

## Abstract

**Background:**

*KCNE1* encodes a 129-residue cardiac potassium channel (*I*_Ks_) subunit. KCNE1 variants are associated with long QT syndrome and atrial fibrillation. However, most variants have insufficient evidence of clinical consequences and thus limited clinical utility.

**Methods:**

In this study, we leveraged the power of variant effect mapping, which couples saturation mutagenesis with high-throughput sequencing, to ascertain the function of thousands of protein-coding KCNE1 variants.

**Results:**

We comprehensively assayed KCNE1 variant cell surface expression (2554/2709 possible single-amino-acid variants) and function (2534 variants). Our study identified 470 loss- or partial loss-of-surface expression and 574 loss- or partial loss-of-function variants. Of the 574 loss- or partial loss-of-function variants, 152 (26.5%) had reduced cell surface expression, indicating that most functionally deleterious variants affect channel gating. Nonsense variants at residues 56–104 generally had WT-like trafficking scores but decreased functional scores, indicating that the latter half of the protein is dispensable for protein trafficking but essential for channel function. 22 of the 30 KCNE1 residues (73%) highly intolerant of variation (with > 70% loss-of-function variants) were in predicted close contact with binding partners KCNQ1 or calmodulin. Our functional assay data were consistent with gold standard electrophysiological data (*ρ* =  − 0.64), population and patient cohorts (32/38 presumed benign or pathogenic variants with consistent scores), and computational predictors (*ρ* =  − 0.62). Our data provide moderate-strength evidence for the American College of Medical Genetics/Association of Molecular Pathology functional criteria for benign and pathogenic variants.

**Conclusions:**

Comprehensive variant effect maps of *KCNE1* can both provide insight into *I*_Ks_ channel biology and help reclassify variants of uncertain significance.

**Supplementary Information:**

The online version contains supplementary material available at 10.1186/s13073-024-01340-5.

## Background

**Fig. 1:**
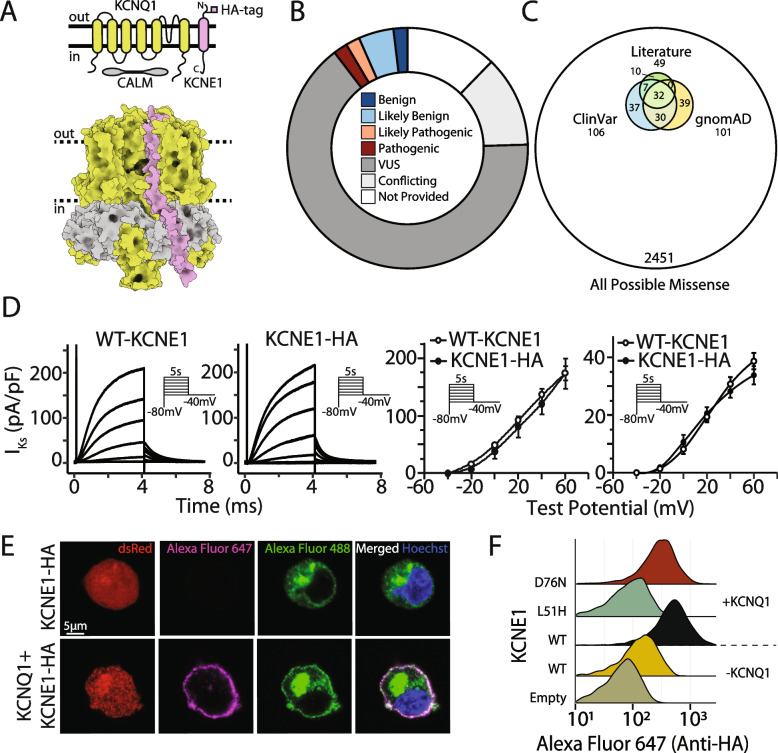
Overview of the *I*_Ks_ channel and a novel location for the HA epitope. **A** The *I*_Ks_ channel complex is formed by KCNE1 (pink), KCNQ1 (green), and calmodulin (gray; top: schematic, and bottom: three-dimensional structure). The HA tag in KCNE1 (cloned between residues 34 and 35) is labeled. **B** Most of the 106 missense KCNE1 variant classifications in the ClinVar database are VUS or conflicting (figure style adapted from Starita et al., 2017). **C** Variants reported in ClinVar, gnomAD, and previous studies comprise a small portion of all possible *KCNE1* missense variants. **D** The HA tag did not disrupt *I*_Ks_ (left: representative traces, inset: voltage protocol, and right: quantification of peak and tail current; *n* = 4 cells/condition). **E** An HA tag was used to visualize KCNE1. Live HEK293T cells were stained with an Alexa Fluor 647-conjugated anti-HA antibody to detect surface KCNE1, then fixed, permeabilized, and stained with an Alexa Fluor 488-conjugated anti-HA antibody to detect total KCNE1. dsRed is the KCNQ1 and KCNE1 transfection marker. Cells expressing only KCNE1-HA showed minimal surface labeling in the absence of KCNQ1 (top). Cells expressing KCNE1-HA and KCNQ1 had normal cell surface expression (bottom). **F** By flow cytometry, live cells stained for extracellular KCNE1-HA had minimal but detectable labeling in the absence of KCNQ1, but 6.1-fold higher labeling in the presence of KCNQ1. Known trafficking-null variant (L51H) had minimal cell surface expression, but a known gating variant (D76N) had near-wild type cell surface expression

Loss-of-function variants in *KCNE1 *can cause type 5 long QT syndrome (LQT5; MIM 613695), a cardiac arrhythmia disorder characterized by QT prolongation on the electrocardiogram and an increased risk of sudden cardiac death [1]. Heterozygous LQT5 variants can cause isolated QT prolongation (also known as Romano-Ward Syndrome) [2], and homozygous and compound heterozygous variants can cause both QT prolongation and deafness (Jervell and Lange-Nielsen Syndrome; MIM 612347) [3]. Almost 50 unique *KCNE1 *variants have been observed in patients with LQT5 [1, 4–6]. In addition, two gain-of-function variants have been associated with atrial fibrillation in isolated families [7]. *KCNE1* encodes a single-spanning transmembrane protein that acts as a modulatory subunit to the pore-conducting KCNQ1 to form the *I*_Ks_ channel critical for cardiac repolarization (Fig. 1A). The *I*_Ks _complex is conventionally thought of as comprising KCNE1 and KCNQ1. However, additional proteins, including calmodulin, interact with the complex to modulate channel electrophysiology [8]. *KCNE1 *variants can disrupt channel function in two major ways: by reducing cell surface expression or by altering gating to reduce potassium flux [9].

The American College of Medical Genetics and Genomics/Association of Molecular Pathology (ACMG/AMP) criteria are used to classify variants as pathogenic, likely pathogenic, likely benign, or benign [10]. Variants with inconclusive evidence for clinical classification are designated as variants of uncertain significance (VUS). For many Mendelian disease genes, including *KCNE1*, most discovered variants are VUS and thus have limited impact on clinical decision-making [10], presenting a major challenge to genomic medicine [11, 12]. In the ClinVar database of variant classifications [13], 77.4% of *KCNE1* variants are either VUS or have conflicting interpretations (Fig. 1B). These variants, together with those reported by the Genome Aggregation Database (gnomAD) [14] or clinical case studies, represent a small subset of all possible missense variants (Fig. 1C). Since it has been proposed that all missense variants compatible with life likely already exist in ~ 50 individuals on the planet, the VUS problem is likely to continue to grow as more individuals are sequenced [15]. In the ACMG/AMP scheme, well-validated in vitro functional studies can provide up to strong-level evidence for the PS3 (damaging effect on protein) and BS3 (no damaging effect) criteria [16, 17]. However, functional assessment lags behind the rate of VUS discovery due to the rapid increase of genetic testing in research and clinical domains [12]. One way to address this VUS problem is to leverage multiplexed assays for variant effects (MAVEs) to test thousands of variants in a single, highly multiplexed experiment [12, 18]. In a MAVE, a comprehensive variant library is coupled to a selection assay and high-throughput sequencing to ascertain variant function.

In this study, we conducted multiplexed assays of KCNE1 variant cell surface expression and function on a library of 2592 single-residue KCNE1 variants (95.7% of 2709 total possible variants, Fig. S1). The two assays were conducted to distinguish between the two mechanisms of KCNE1 deleteriousness: loss of cell surface expression and altered ion efflux through channels with otherwise unaffected cell surface expression. We used an HA epitope in KCNE1 to determine cell surface expression of 2554 individual variants using a “landing pad” cell line [19], which integrates a single variant per cell.

This assay identified 470 missense variants that decrease and 310 that increase cell surface expression. We also used a gain-of-function KCNQ1 variant to design a cell fitness assay that selects against cells with functioning channels to ascertain KCNE1 variant function. We deployed the functional assay on 2534 KCNE1 variants and found 574 loss- or partial loss-of-function missense variants, only 152 of which also had reduced cell surface expression. These datasets correlate well with previously validated in vitro studies, computational predictors, population metrics, and clinical phenotypes [6, 14, 20]. Our data provide insight into KCNE1 structure and biology and can be implemented to reclassify variants in the ACMG/AMP scheme.

## Methods

### Overview of multiplexed assays of KCNE1 variant effect

A hemagglutinin (HA) epitope tag was cloned into the extracellular region of *KCNE1*. Saturation mutagenesis was used to create a plasmid library of 2592 distinct single-amino-acid *KCNE1* variants (95.7% of total possible variants). Each plasmid was associated with a random 18-base barcode and the plasmid library was “subassembled,” i.e., deep sequenced to associate each barcode to its corresponding *KCNE1* variant. To express one *KCNE1*-HA variant per cell, the library was integrated into Human Embryonic Kidney (HEK) 293T cells engineered to include a “landing pad.” [19] The landing pad is a single promoter expression system with standardized locus expression [21]. To determine cell surface expression, *KCNE1*-HA variants were expressed with or without WT *KCNQ1* and the cell pool was stained with an anti-HA antibody. Cells were sorted into 4 bins based on the level of cell surface labeling, and each bin was deep sequenced to quantify variants. To determine channel function, *KCNE1-HA* variants were expressed with a gain-of-function *KCNQ1* variant for 20 days to deplete cells with normally functioning *I*_Ks_ channels. The cell pool was deep sequenced at days 0, 8, and 20 to quantify variants (Fig. S1). Each assay was conducted in triplicate, and each variant was represented by an average of 30.7 barcodes in each replicate, allowing for internal and external validation.

### Cloning a novel KCNE1 HA tag

We used *KCNE1 *(NCBI Reference Sequence NM_001127670.4) with the G allele at residue 38 (p.S38G, c.112A > G, rs1805127), as this variant allele is more common globally (65.8% allele frequency across all ancestral populations in gnomAD; range 57.4–71.3%) [14]. An HA epitope (5′-TACCCCTACGATGTACCAGATTATGCG-3′) was cloned between residues 34 and 35 with primers ag424 and ag425 using inverse PCR with Q5 polymerase (New England Biolabs, NEB) [22]. The sequences of all primers used in this study are presented in Table S1. The resulting gene, *KCNE1*-HA, was subcloned into the pIRES2-dsRED2 plasmid using PCR with ag409 and ag410 and NotI restriction digestion (NEB) to create p*KCNE1-*HA:pIRES2:dsRED2. All plasmid sequences were verified with Sanger sequencing. Using NotI restriction digestion, the *KCNE1*-HA gene was moved to an AttB plasmid containing pIRES:mCherry:BlastR to create p*KCNE1*-HA:IRES:mCherry:BlastR. We also used a compact (< 4 kb) promoterless plasmid for single-variant mutagenesis [23]. Maps of key plasmids used in this study are shown in Fig. S2A.

### Generation of HEK293T lines stably expressing KCNQ1 or KCNQ1-S140G

The experiments were conducted in either previously published Bxb1-mediated landing pad HEK293T (LP) cells [19], or LP cells genetically engineered to express *KCNQ1 *(WT or S140G). LP cells contain an AttP integration “landing pad,” Blue Fluorescent Protein (BFP), and iCasp9 caspase downstream of a doxycycline-inducible promoter [19]. The AttB:mCherry:BlastR plasmid integrates into the AttP landing pad. Prior to integration, the promoter drives BFP and iCasp9 expression. After integration, the promoter drives expression of mCherry and blasticidin resistance genes instead. Cells with a successful plasmid integration event are blasticidin resistant, express mCherry, and lack iCasp9, which can be used to select for recombined cells. Genetically engineered LP cells expressing *KCNQ1* (WT or S140G) were generated as below.

Since we used NotI restriction digestion for subcloning, we removed an intra-cDNA NotI restriction enzyme site, by introducing a synonymous mutation (c.246G > T), into WT *KCNQ1* cDNA (NM_000218.3) using QuikChange mutagenesis (Agilent). The *KCNQ1* gain-of-function variant (p.S140G, c.418A > G, rs120074192) was also generated by QuikChange mutagenesis. KCNQ1 S140G biases *I*_Ks_ channels to the open state [24, 25] at lower membrane potentials (including the resting potential of HEK293T cells, approximately −25 mV) [26] and leads to increased cellular K^+^efflux [27].

To construct a cell line with constitutive *KCNQ1* expression, *KCNQ1 *(WT or p.S140G) was cloned into a Sleeping Beauty transposon system, pSBbi-GN (a gift from Eric Kowarz, Addgene# 60517) [28], using SfiI restriction digestion (NEB) to create pSBbi-GN::KCNQ1. This plasmid expresses *KCNQ1* and *EGFP* via constitutively active bidirectional promoters. The plasmid was randomly integrated into a non-landing pad locus in the LP cells with the Sleeping Beauty transposase as follows. Cells were cultured at 37° at 5% CO_2_in HEK media: 10% FBS (Corning), 1% non-essential amino acids (Corning), 1% penicillin/streptomycin (Corning) in DMEM (Thermo). On day 0, pSBbi-GN::*KCNQ1* was transfected into the LP cell line along with a plasmid expressing the Sleeping Beauty transposase, pCMV(CAT)T7-SB100 (a gift from Zsuzsanna Izsvak, Addgene #34879) [29], using FuGENE6 (Promega) in a 1µg plasmid:3ul FuGENE ratio with DMEM. On day 6, the cells were exposed to HEK media with 1 µg/mL doxycycline (Sigma) to induce expression of the BFP/iCasp9 from the landing pad. The cells were sorted with a BD FACSAria IIIU (BD Biosciences), using a 100 µm nozzle for cells with high levels of BFP and high levels of GFP (Fig. S2B) into HEK media on amine-coated plates (Corning). Single-cell colonies were grown in HEK media, with the addition of 1 µg/mL doxycycline 48 h before being screened on a BD LSRFortessa SORP (BD Biosciences) to identify colonies with high rates of both landing pad (*BFP*) and *KCNQ1* (*GFP*) expression (Fig. S2C). Two distinct colonies with high blue and green fluorescence were expanded for further experiments. The landing pad cell line expressing WT KCNQ1 is referred to as LP-KCNQ1 and the cell line expressing KCNQ1 S140G is referred to as LP-KCNQ1-S140G.

For transient transfection experiments, plasmids that constitutively expressed *KCNE1* or *KCNQ1* were cloned using NotI restriction digestion into pIRES2:EGFP or pIRES2:dsRED2 to create all four combinations: p*KCNE1*:IRES:EGFP, p*KCNE1*:IRES:dsRED2, p*KCNQ1*:IRES:EGFP, and p*KCNQ1*:IRES:dsRED2 (see *Supplemental* *Methods* for more details).

### KCNE1 library creation

We used custom python scripts to design 129 primer pairs, one for each of the 129 KCNE1 residues, with a target T_m_ of 58° using the formula below:

$$T_m=4\times\left(\#\;of\;Gs\;and\;Cs\right)+2\times\;\left(\#\;of\;As\;and\;Ts\right)$$

The mutagenesis primers are presented in Table S2. Each residue was comprehensively mutated using inverse PCR on the compact template plasmid [22, 23]. For each mutagenesis reaction, the forward primers contained a 5' degenerate NNK sequence for each codon to introduce all 20 sense amino acid variants and 1 nonsense variant at each site. The 129 PCR reactions were pooled, PCR-purified (QIAGEN), phosphorylated with T4 PNK (NEB), and self-ligated using T4 ligase (NEB). The pooled product was re-PCR-purified and electroporated into ElectroMAX DH10B Cells (ThermoFisher) using a Gene Pulser Electroporator (Bio-Rad; 2.0 kV, 25 µF, 200 Ω). Serial dilutions of the electroporated cells were plated on Ampicillin plates to determine transformation efficiency. The library was purified using a MaxiPrep kit (Qiagen) and subcloned into a promoterless AttB pIRES:mCherry-BlastR [19, 23] plasmid using restriction digestion with AatII and Aflll (NEB). The library was then re-electroporated and purified as above.

For quantification, each variant construct was tagged with a random 18mer barcode to the library plasmid. The barcode was generated using ag289 (spacer-BsiWI-AflII-N18-AatII-XbaI-spacer) and ag290 (reverse complement of the 3′ end of ag289). The two primers were annealed by incubating at 95° for 3 min and cooling by 1°/10 s. The annealed primers were extended to fully double-stranded DNA using Klenow polymerase (NEB). The barcode was purified by phenol–chloroform extraction, digested with BsiWI and XbaI (Thermo Fisher), and re-purified by phenol–chloroform extraction. The resulting DNA fragment was ligated into the *KCNE1*-HA plasmid library. The plasmid library was deep sequenced to link each barcode to its associated variant (see *Supplemental* *Methods* ). A map of the final barcoded plasmid library (p*KCNE1*-HA library:pIRES:dsRed:BlastR) is provided in Fig. S3A.

### Integration of library into landing pad cells

LP, LP-KCNQ1, or LP-KCNQ1-S140G cells were cultured in 10-cm dishes in HEK media until approximately 60% confluent. Cells were then transfected with 25 µg of p*KCNE1*-HA library:IRES:dsRed:BlastR and 5 µg of site-specific recombinase, pCAG-NLS-HA-Bxb1 (a gift from Pawel Pelczar, Addgene #51,271) [30] using Lipofectamine 2000 (Thermo Fisher) in a 1µg plasmid:2ul Lipofectamine ratio [19]. 6–8 h after transfection, the cells were fed fresh media. The following day, cells were passaged using Accutase (Sigma) into two 10 cm dishes and fed media supplemented with 1 µg/mL doxycycline (Sigma) to induce expression of the landing pad. With successful integration of a single AttB-containing plasmid, the landing pad expresses only one *KCNE1*-HA variant, mCherry, and BlastR. Twenty-four hours later, cells were singularized in place using 1 mL Accutase, quenched with 10 mL selection media (HEK media as above with 1 µg/mL doxycycline, 100 µg/mL blasticidin (Sigma), and 10 nM AP1903 (MedChemExpress)). Addition of blasticidin and AP1903 selected for successfully integrated cells that expressed p*KCNE1*-HA library:pIRES:mCherry:BlastR and against non-integrated cells that continued to express BFP/iCasp9 caspase. Cells were grown in selection media for 8 days post-transfection. For the LP-KCNQ1-S140G line, cells with successful integration were enriched in HEK selection media containing a high concentration (500 nM) of the HMR 1556 (Tocris), a specific *I*_Ks_ blocker with an IC_50 _of 10.5 nM [31].

### Trafficking assay

We measured cell surface trafficking of the *KCNE1*-HA library in the presence and absence of *KCNQ1*, using LP-KCNQ1 and LP cells, respectively. Each experiment was performed in triplicate (three independently transfected, stained, sorted, and sequenced replicates). As a general overview for the trafficking MAVE assay, we (1) generated a pool of HEK293 cells, each expressing one *KCNE1* variant from the *KCNE1* variant library, (2) labeled cell surface abundance of KCNE1 using an anti-HA antibody covalently bound to a fluorophore, (3) flow sorted the pool of cells into four groups based on fluorescence (indicating KCNE1 surface abundance), (4) isolated DNA from each of the four sorted pools and deep sequenced these samples to quantify the barcodes present in each pool, and (5) used each variant’s abundance in each of the four sorted pools to quantify cell surface abundance of each variant.

To stain KCNE1-HA at the cell surface, day 8 post-selection, cells were dissociated using Accutase and quenched with HEK selection media. For any subsequent cellular suspension, cells were centrifuged for 5 min at 300* g* and suspended in the new solution. The cells were washed with block solution (1% FBS + 25 mM HEPES pH 7.0 [Sigma] in PBS without Ca^2+^/Mg^2+^). The cells were then stained with 1:500 anti-HA Alexa Fluor 647 conjugated antibody (Cell Signaling #3444) in block solution for 45 min at room temperature in the dark and washed three times. Stained cells were resuspended in block solution at a concentration of 4–6 million cells/mL for sorting. Two trafficking experimental replicates were performed with cells in suspension as described above. One replicate was stained while cells were adhered on the dish (see *Supplemental* *Methods* ).

Stained cells were FACS sorted on the BD FACSAria III (BD Biosciences) using a 100-µm nozzle. Compensation was performed using cells expressing single fluorophores or Alexa Fluor 647 compensation beads (Thermo Fisher). Single cells were identified based on forward and side scatter signals. In order to simultaneously assay pools of thousands of barcoded mutant plasmids, we did not individually assay each plasmid. Instead, similar to previously described approaches [32, 33], we sorted large pools of cells expressing pools of mutants into four groups of approximately equal size (~ 25% each) based on cell surface KCNE1 labeling for downstream sequencing. Specifically, BFP^−^ /mCherry^+^ single cells (with and without GFP for *KCNE1* with and without *KCNQ1* experiments, respectively) were sorted into 4 groups of approximately equal size based on Alexa Fluor 647 labeling (Fig. S4). At least 800,000 cells per group were sorted and replated onto amine-coated plates containing HEK media. Cells were expanded and genomic DNA was extracted from each group with the Quick-DNA midiprep kit (Zymo). Q5 polymerase was used to amplify the barcodes from gDNA with 20 cycles of amplification using Illumina-compatible primer pairs (Tables S1 and S3), and the barcodes were sequenced on the Illumina NovaSeq PE150 with at least 25 million reads per sample. Normalized cell surface trafficking scores were calculated from a weighted average of barcode counts from these samples (see *Supplemental* *Methods* for details). Variants were divided into 6 categories: loss-of-trafficking, partial loss-of-trafficking, possible loss-of-trafficking, normal trafficking, possible gain-of-trafficking, or gain-of-trafficking. Loss-of-trafficking variants were defined as variants with trafficking score point estimates less than 97.5th percentile of the early nonsense variant score distribution (0.2). Partial loss-of-trafficking variants were defined as variants with score estimates (95% confidence interval) between 0.2 and 0.83 (2.5th percentile of the synonymous variant scores). Possible loss-of-trafficking variants were defined as variants with a point estimate near 0.83, and the confidence interval for the variant trafficking score spanning 0.83. Normal trafficking variants were defined as those with score estimates (95% confidence interval) between 2.5th and 97.5th percentile respectively (0.83 and 1.18). Possible gain-of-trafficking variants were defined as variants with a point estimate near 1.18, and the confidence interval for the variant trafficking score spanning 1.18. Gain-of-trafficking variants were defined as variants with a point estimate and confidence interval above 1.18.

### Functional assay

The functional assay was validated by 1:1 competition experiments on control variants (see *Supplemental* *Methods* for details) and then conducted on the entire library. The comprehensive library was integrated into the landing pad of cells as described above. Fitness of cells expressing *KCNE1*-HA variants in the library was measured in LP-KCNQ1-S140G in three independent replicates. On the 8th day after successful plasmid integration, termed day 0 in subsequent analyses, cells were washed with selection media to remove HMR 1556, and a subset of cells harvested for gDNA extraction. Cells were passaged as needed (when cells were near confluent). Additional samples were harvested for gDNA extraction on day 8 and day 20. Genomic DNA from these three time points (days 0, 8, and 20) was isolated. Barcode pools were amplified and sequenced using the Illumina NovaSeq as described above. Variants were divided into 6 categories: loss-of-function, partial loss-of-function, possible loss-of-function, normal function, possible gain-of-function, or gain-of-function. Loss-of-function variants were defined as variants with functional score point estimates less than 97.5th percentile of the early nonsense variant score distribution (0.09). Partial loss-of-function variants were defined as variants with score estimates (95% confidence interval) between 0.09 and 0.44 (2.5th percentile of the synonymous variant scores). Possible loss-of-function variants were defined as variants with a point estimate near 0.44, and the confidence interval for the variant functional score spanning 0.44. Normal function variants were defined as those with score estimates (95% confidence interval) between 2.5th and 97.5th percentile respectively (0.44 and 1.53). Possible gain-of-function variants were defined as variants with a point estimate near 1.53, and the confidence interval for the variant functional score spanning 1.53. Gain-of-function variants were defined as variants with a point estimate and confidence interval above 1.53.

### Population-level analyses

The data for putative controls were obtained from gnomAD v.2.1.1 (exomes and genomes, accessed October 2022) [14]. Variant annotations were obtained from the ClinVar database (accessed October 2022, Supplemental file 1) [13]. In addition, we performed a literature review that consisted of a PubMed search (on November 30, 2022) for “KCNE1.” The resulting 929 manuscript titles and abstracts were manually reviewed to collate published patch clamp electrophysiology and patient data (Supplemental file 2, see *Supplemental* *Methods* for more details). Variants in at least 1 patient with LQT5 (Romano-Ward or one allele of JLNS) and an allele frequency < 2.5 × 10^−5^ across all ancestries in gnomAD were considered putative LQT5-associated variants. Similarly, the 2 variants previously seen in cases with atrial fibrillation, G25V and G60D, also had allele frequencies < 2.5 × 10^−5^, and thus were considered putative atrial fibrillation-associated variants. Putative benign variants had an estimated penetrance of < 10% [*(# cases in literature)/(# of total carriers in literature* + *allele counts in gnomAD)*] and had an allele frequency of > 3 × 10^−5^ in gnomAD. Only 1 variant (G55S) with an allele frequency of > 3 × 10^−5^ had a penetrance of > 10% and was excluded.

### SpliceAI scores

Precalculated SpliceAI scores for all coding variants on chromosome 7 for hg19 were downloaded from Illumina Basespace (Supplemental file 3) [34]. These were filtered for the genomic interval containing *KCNE1* (chr21:35,821,932–35,821,545) using tabix [35] and processed in Excel and R to determine the cDNA and protein change corresponding to each genomic coordinate variant.

### ACMG/AMP assay calibration and comparison

Missense variants classified in ClinVar as pathogenic/likely pathogenic or in our curated list of putative LQT5-associated variants (in at least 1 patient with an allele frequency < 2.5 × 10^−5^ across all ancestries in gnomAD) were considered “presumed pathogenic.” Missense variants classified as benign/likely benign in ClinVar or in our curated list of putative normal variants (estimated penetrance of < 10% and allele frequency of > 3 × 10^−5 ^in gnomAD) were considered “presumed benign” (Table S4). Variants with score estimates (mean and 95% confidence interval) below the 2.5th percentile (0.44) or above the 97.5th percentile (1.43) of the synonymous distribution were defined as loss-of-function and gain-of-function respectively. Variants with score estimates within the cutoffs were considered “functionally WT-like.” Missense variants score estimates that overlapped the cutoffs were considered “indeterminate.” To determine the strength of evidence at which MAVE scores could be used as evidence for the ACMG/AMP functional assay criteria (PS3/BS3), the assay OddsPath was calculated using previously described methods [17]. An interactive worksheet used to calculate OddsPath scores is presented in Supplemental file 4.

## Results

### Validation of an extracellular KCNE1 HA tag to detect cell surface expression

We cloned a 9-residue hemagglutinin (HA) tag into the extracellular domain of *KCNE1* (between residues 33 and 34; Fig. 1A). KCNE1-HA (33–34) resulted in threefold higher cell surface labeling of the *I*_Ks _channel complex compared to a previously described HA tag located in a predicted alpha helix (Fig. S5A) [36]. Neither *I*_Ks_ peak and tail current densities, nor activation properties were significantly different between KCNE1-WT and KCNE1-HA (Fig. 1D). Human Embryonic Kidney (HEK) 293T landing pad cells expressing *KCNE1*-HA were labeled with a fluorophore-conjugated anti-HA antibody, visualized by confocal microscopy, and quantified by flow cytometry (Fig. 1E, F). In cells expressing *KCNE1*-HA only, minimal plasma membrane anti-HA labeling was present, consistent with previous studies [9, 37]. In contrast, cells coexpressing both *KCNE1*-HA and *KCNQ1* demonstrated substantial plasma membrane anti-HA labeling in live cells, and both plasma membrane and intracellular labeling in permeabilized cells (Fig. 1E) [9, 38]. Quantification of cell surface expression by flow cytometry confirmed these observations (Fig. 1F). A minimal but detectable amount of plasma membrane labeling was seen in *KCNE1*-HA only cells but the addition of *KCNQ1* caused a 6.1-fold increase in cell surface expression (Figs. 1F and S2B). As expected, a known loss-of-trafficking variant (L51H) had minimal cell surface expression and a known gating variant (D76N) had near-WT cell surface expression when coexpressed with *KCNQ1* (Figs. 1F and S5B) [39].

### A comprehensive library of KCNE1 mutations

We generated a comprehensive variant library of the 129-residue KCNE1 protein associated with random 18-mer barcodes using inverse PCR mutagenesis with degenerate primers (see “Methods,” Fig. S3A) [22]. We linked 80,282 barcodes to 2368 missense, 100 synonymous, and 124 nonsense KCNE1 variants (95.7% of the total possible variants; Fig. S3B). Each variant was represented by a mean of 31 barcodes (Fig. S3C).

### Multiplexed assay of KCNE1 cell surface expression

**Fig. 2:**
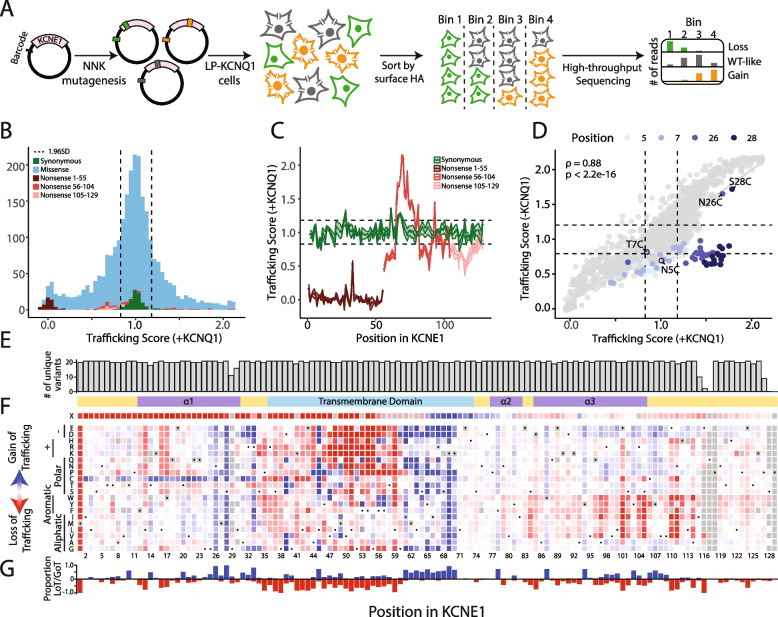
Multiplexed assay of *KCNE1* cell surface expression. **A** To conduct the cell surface expression multiplexed assay of *KCNE1*, we used an HA epitope in KCNE1, constructed a comprehensively mutated barcoded plasmid pool, and integrated the library into the LP-KCNQ1 cell line. Cells were stained with a fluorophore-conjugated anti-HA antibody, sorted cells into 4 bins by surface KCNE1 levels and each pool was deep sequenced. Variant abundance in each sequenced pool was used to calculate a trafficking score for each variant. The diagram shows three example variant plasmids, each denoted by a different color. The three example variants have different levels of KCNE1-HA surface expression (green: loss-of-trafficking, gray: normal trafficking, yellow: high trafficking), resulting in different patterns of sequencing results in each of the 4 sorted cell pools. **B** Histogram of normalized trafficking scores by functional class showed a unimodal distribution of missense variant scores centered at the median of synonymous variant scores. A smaller peak at the median of early nonsense variant scores and a tail of super-trafficking variant scores was also seen (Dotted lines: mean ± 1.96 SD for the synonymous distribution). **C** Most nonsense variants (1 per residue) from residues 1–55 (brown) are loss-of-trafficking but nonsense variants after residue 55 (red or pink) are gain-of-trafficking or WT-like. Synonymous variants (1 per residue) are represented in green (Dotted lines: mean ± 1.96 SD for the synonymous distribution). **D** Trafficking scores in the presence and absence of KCNQ1 were highly correlated with the exception of variants at residues 5, 7, 26, and 28 which disrupted glycosylation sites (highlight). Cysteine variants at these sites did not follow this trend (annotation). See Fig. S5 for a full description of the KCNQ1^−^ trafficking assay. Dotted lines: mean ± 1.96 SD for the synonymous distribution. **E** Near complete representation of variants was scored at each position in the KCNE1 protein (max = 21). **F** A heatmap showing trafficking scores. Red (score = 0), white (score = 1), and blue (score = 2) indicate low, WT-like, and high cell surface expression, respectively. Dots in squares indicate WT amino acids at that position. The colored ribbon indicates secondary structure (purple = alpha helices, light blue = transmembrane domain, yellow = unstructured regions). **G** For each residue, the proportion of gain-of-trafficking (GoT) and loss-of-trafficking (LoT) missense variants is displayed

We coupled antibody labeling of the extracellular HA tag to deep sequencing of the library barcodes to perform a multiplexed assay of KCNE1 cell surface expression in cells engineered to constitutively express *KCNQ1 *(Fig. 2A). We used a landing pad cell line that integrated a single KCNE1 cDNA construct downstream of a preexisting promoter in each cell, allowing standardized expression of a single protein variant per cell [19]. The cells were then stained with a fluorophore-conjugated anti-HA antibody, and sorted into four groups based on surface labeling of HA. Variant counts in each group were quantified using high-throughput sequencing (see “Methods,” Figs. 2A and S6A). After quality control, we obtained cell surface expression scores (see *Supplemental* *Methods* ) for 98 synonymous, 117 nonsense, and 2339 missense variants (94.2% of all possible variants; Fig. 2B). Scores calculated from three individual replicates were highly consistent (Spearman’s *ρ* = 0.83–0.89 for pairwise comparisons, *p* < 0.001; Fig. S6B). Cell surface trafficking scores for synonymous variants were normally distributed (0.988–1.024, 95% confidence interval, Fig. 2B,C). Nonsense variant scores were bimodally distributed (mode 1 = 0.02, mode 2 = 0.74). Nonsense variants from residue 1 to 55 were all trafficking-deficient, whereas nonsense variants at or after residue 56 had near-WT or higher than WT cell surface expression (*p* = 2.5 × 10^−20^, Wilcoxon test; Fig. 2B,C), indicating that only 55 residues are needed for KCNE1 expression at the cell surface. We refer to early nonsense variants (residues 1–55) and late nonsense variants (residues 56–129) in descriptions of trafficking experiments below and in the figures. All variants at the residue 1 start codon were complete loss-of-trafficking as expected.

Variants were divided into 6 categories: loss-of-trafficking, partial loss-of-trafficking, possible loss-of-trafficking, normal trafficking, possible gain-of-trafficking, or gain-of-trafficking. We identified 105 loss-of-trafficking, 365 partial loss-of-trafficking, and 310 gain-of-trafficking missense variants (Table S5). We also compared trafficking scores to the few literature reports of cell surface trafficking measurements, typically reported as “normal” or “loss-of-trafficking.” 7/9 variants were consistent: 6 variants with normal or near-normal trafficking (T6F, S74L D76A, D76N, Y81C, and W87R) and 1 loss-of-trafficking variant (L51H; Fig. S6C) [39–42]. Two variants, R98W and G52R, were previously reported to have normal trafficking but had reduced trafficking scores in our assay (R98W: 0.192–0.637, and G52R: − 0.033–0.247, 95% confidence interval) [9]. We conducted individual cell surface expression studies of these variants and found them to have mean trafficking levels of 53.0% (R98W) and 4.5% (G52R; normalized to WT, Fig. S6D), consistent with their partial loss-of-trafficking and loss-of-trafficking MAVE scores. After inclusion of our repeat validation experiments, the MAVE trafficking scores were fully consistent with individually studied variants (Fig. S6E).

Several interesting and previously unreported findings emerged from the cell surface trafficking scores (Fig. 2F). As mentioned above, nonsense variants after residue 55 were still able to traffic to the cell surface. In the extracellular N-terminus, almost all cysteine variants from residue 2 to 33 had increased cell surface expression. Residues 46–59 in the transmembrane helix, likely juxtaposed to the hydrophobic core of the lipid bilayer, were highly intolerant to polar or charged variation. Variants in residues 61–70, interacting with the intracellular hydrophilic edge of the cell membrane, increased cell surface expression. Predicted alpha helices in the intracellular C-terminus (residues 79–114) had multiple residues intolerant to aromatic or larger aliphatic variation.

As HEK293T cells expressing only KCNE1 exhibited detectable levels of anti-HA staining by flow cytometry, we conducted a second MAVE of KCNE1 cell surface expression in the absence of coexpressed KCNQ1 (“KCNQ1^−^ ”; Fig. S7). Trafficking scores for missense variants in the presence and absence of KCNQ1 were highly correlated (Fig. 2D, Spearman’s *ρ* = 0.88, *p* < 2 × 10^−6^), except for variants in the N-glycosylation sites (NXS/NXT motifs) at positions 5–7 and 26–28 [42, 43]. Overall, glycosylation variants comprised 39/50 variants with the largest difference in trafficking scores in the presence and absence of KCNQ1. In the presence of KCNQ1, most glycosylation variants at residues 5 and 7 had normal or near-normal trafficking compared to WT (28/34 normal, possible loss-, or possible gain-of-trafficking; Fig. 2D). However, in the absence of KCNQ1, most glycosylation variants at residues 5 and 7 were partial loss- or possible loss-of-trafficking compared to WT (33/34). On the other hand, in the presence of KCNQ1, almost all glycosylation variants at residues 26 and 28 had gain-of-trafficking scores compared to WT (31/32). However, in the absence of KCNQ1, only N26C and S28C had gain-of-trafficking scores compared to WT, and 27/32 had partial- or possible loss-of-trafficking scores. Thus, most variants disrupting N-glycosylation had higher trafficking scores with coexpression of KCNQ1 when compared to in the absence of KCNQ1, and this increase was especially pronounced at residues 26 and 28 (Fig. 2D). All subsequent mentions of trafficking scores refer to the trafficking assay conducted in the presence of KCNQ1.

### Development of a cell fitness assay for KCNE1 function

**Fig. 3:**
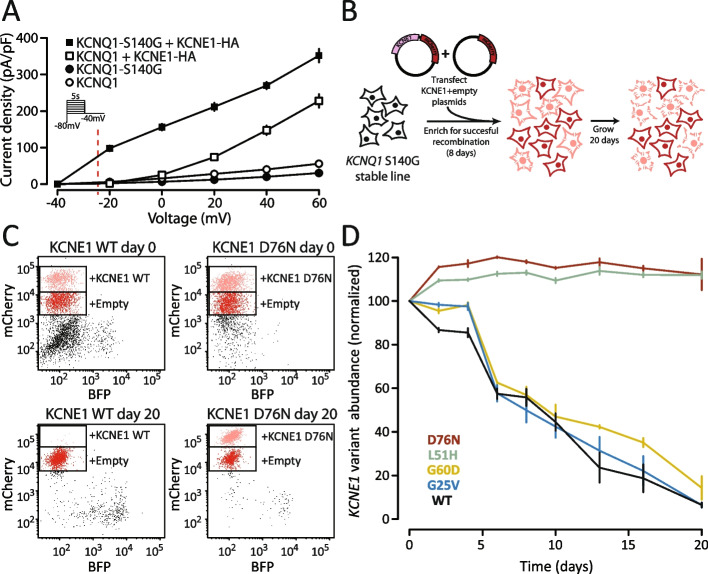
A KCNE1 functional assay using a gain-of-function KCNQ1 variant. **A** Voltage clamp measurements of outward potassium currents in HEK293T cells transfected with various combinations of WT or S140G KCNQ1 and/or KCNE1-HA. At the resting potential of HEK293T cells (~ −25 mV, dotted line), there is almost no potassium current in cells transfected with KCNQ1 ± KCNE1-HA. However, the gain-of-function KCNQ1-S140G variant results in a leftward shift of voltage of activation and increased current at −25 mV, only in the presence of KCNE1. **B** Experiment to validate KCNE1 selection assay using LP-KCNQ1-S140G cells transfected with 1:1 pools of KCNE1 variant (pink) and empty vector (red) plasmids. **C** Representative flow cytometry measurements of cells transfected with 1:1 pools of plasmids. Cells with empty vectors had reduced mCherry fluorescence allowing quantification of distinct cell populations. After 20 days of selection, there was a strong depletion of cells expressing KCNE1-HA-WT but not a loss-of-function variant D76N. **D** Time course of relative fitness of cells expressing KCNE1 variants compared to cells expressing empty vectors. All values are normalized to the values at day 0. Cells expressing KCNE1-HA-WT or two gain-of-function variants (G25V and G60D) depleted over 20 days but cells expressing two loss-of-function variants (D76N and L51H) persisted in the cell pool. Points and error bars indicate mean and standard error of three replicates

Since *I*_Ks _channels are closed at the resting potential of HEK293T cells (approximately −25 mV) [26], we developed an assay for KCNE1 function by leveraging the electrophysiology of a previously described gain-of-function variant, KCNQ1-S140G [25, 44]. In the presence of KCNE1, KCNQ1-S140G left-shifts the voltage of *I*_Ks_ channel activation [25] and reduces channel deactivation [24]; in the absence of KCNE1, the channel expresses minimal current (Fig. 3A). These electrophysiological shifts result in substantial KCNE1-dependent K^+^ efflux at −25 mV. We hypothesized that long-term expression of *I*_Ks_ channels formed by KCNQ1-S140G and normally functioning KCNE1 would increase K^+^ efflux and thereby decrease cell fitness. To test this hypothesis, we integrated a 1:1 mixture of empty vector control and either wildtype KCNE1-HA or previously studied KCNE1 variants into HEK293T landing pad cells stably expressing KCNQ1-S140G (LP-KCNQ1-S140G, Fig. 3B). We then quantified KCNE1^+^ and KCNE1^−^ cell proportions over 20 days (Fig. 3C, D). Compared to cells with empty vector controls, cells with WT KCNE1 depleted over time, whereas cells with loss-of-function KCNE1 variants (L51H or D76N) persistently survived. Two previously described KCNE1 gain-of-function variants (G60D and G25V) [7] depleted at a similar rate as KCNE1-HA-WT (Fig. 3D), indicating that this assay is not well-powered to distinguish gain-of-function variants from WT.

In order to quantify the expression levels of *KCNQ1* and *KCNE1* in our experiments, we performed RNA sequencing of two stable lines (in 3 replicates each) expressing *KCNQ1* (wildtype or S140G), with *KCNE1* integrated into the landing pad. We quantified *KCNQ1* and *KCNE1* expression, measured as fragments per kilobase mapped (FPKM; Table S6). *KCNQ1* had a mean FPKM of 629 and 1065 in the wildtype and S140G lines, respectively. *KCNE1* had a mean FPKM of 83 and 46, respectively.

### Multiplexed assay of KCNE1 function

**Fig. 4:**
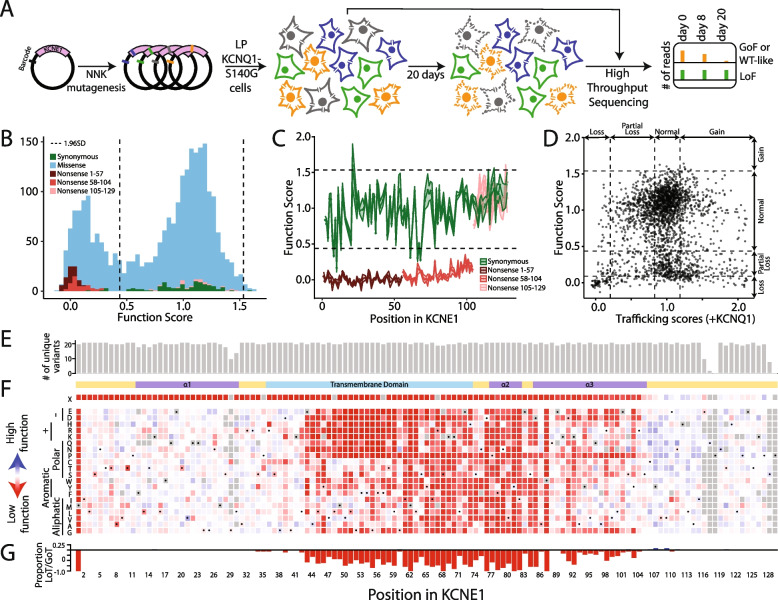
Multiplexed assay of *KCNE1* function. **A** Schematic of multiplexed assay of *KCNE1* function. A comprehensively mutated barcoded plasmid pool was integrated into LP-KCNQ1-S140G cells. Cells were grown for 20 days and cell pools at 0 days, 8 days, and 20 days were deep sequenced. Different cell colors represent example KCNE1 variants (green: loss-of-function, blue: low function, gray: normal function, yellow: high function). Cells with functional KCNE1 variants were depleted from the cell pool over time. **B** Histogram of normalized functional scores by functional class showed a bimodal distribution of missense variants (Dotted lines: mean ± 1.96 SD for the synonymous distribution). **C** Nonsense variants (1 per residue) up to residue 104 are functionally deleterious (brown and red), including residues 56–104 that were dispensable for cell surface trafficking (red). Synonymous variants (1 per residue) are represented in green (Dotted lines: mean ± 1.96 SD for the synonymous distribution). **D** Relationship between trafficking scores in the presence of KCNQ1 and functional scores for missense variants showed that most deleterious variants disrupt gating (Dotted lines: mean + 1.96SD for the nonsense distribution, and mean ± 1.96 SD for the synonymous distribution). **E** Near complete representation of missense variants was scored at each position in KCNE1 (max = 21). **F** KCNE1 functional score heatmap. Red (score = 0), white (score = 1), and blue (score = 2) indicate loss-of-function, normal function, and gain-of-function, respectively. Dots in squares indicate WT amino acids at that position. The colored ribbon indicates secondary structure as in Fig. 2. **G** For each residue, the proportion of loss-of-function missense variants is displayed

We used this KCNQ1-S140G-based cell fitness assay to conduct a multiplexed assay of KCNE1 function. We hypothesized that *KCNE1* variants that decrease *I*_Ks_ (through multiple mechanisms such as reduced peak current or severe alterations to channel gating) would persist in the cell pool over time, whereas those with normal or increased *I*_Ks_ would deplete from the pool. To conduct the assay, we integrated the KCNE1 library into HEK293T cells stably expressing KCNQ1-S140G and quantified cell fitness over 20 days (Fig. 4A). After 20 days, cell expressing synonymous variants were depleted and those expressing most nonsense variants persistently survived (Fig. S8A). We also found that depletion of missense and nonsense variants from the cell pool expressing the comprehensive library was only observed against the background of KCNQ1-S140G (Fig. S8B). These changes over time were not seen in the presence or absence of KCNQ1-WT (Fig. S8B), suggesting that selection against cells with functional channels is due to the gain-of-function S140G variant. Variant depletion from day 0 to day 20 was used to calculate normalized KCNE1 functional scores for 98 synonymous, 121 nonsense, and 2320 missense variants (93.7% of all variants; Figs. 4B and S8C). Functional scores across three replicates were highly consistent (Spearman’s *ρ* = 0.81 for all 3 pairwise comparisons; Fig. S8D). Synonymous variants followed an asymmetric unimodal distribution (median = 0.99, IQR = 0.27, Fig. 4B). Nonsense variants were bimodally distributed (mode 1 = 0.02, mode 2 = 1.08); nonsense variants at residues 1–104 were loss-of-function, whereas nonsense variants at residue 105–129 had WT-like function (*p* = 2.6 × 10^−13^, Wilcoxon test, Fig. 4C). Thus, across the trafficking and functional assays, three broad classes of nonsense variants were present based on residue number: #1–55 caused loss of trafficking and function, #56–104 caused WT-like or elevated trafficking but loss-of-function, and #105–129 caused near WT-like trafficking and function (Fig. S9A). All variants at residue 1 were loss-of-function as expected. Missense variant functional scores were bimodally distributed (mode 1 = 0.1, mode 2 = 1.12). The two modes approximately corresponded to the peaks of the early nonsense and synonymous distributions. Variants were divided into 6 categories: loss-of-function, partial loss-of-function, possible loss-of-function, normal function, possible gain-of-function, or gain-of-function. We identified 173 loss-of-function, 401 partial loss-of-function, and 1 gain-of-function missense variants (Table S7).

There was a complex and statistically significant relationship between trafficking and functional scores (*p* < 2.2 × 10^−16^, chi-square test; Fig. S9B-D and Table S8). 84/105 (80%) loss-of-trafficking variants had partial, possible, or loss-of-function scores (Table S8, Fig. S9D). This includes known LQT5-associated trafficking and function-disrupting variants such as L51H [39]. However, only 103/365 (28.2%) of the partial loss-of-trafficking variants fell into loss-of-function categories. We validated this finding by patch clamping R32T, a partial loss-of-trafficking variant (0.54) with a normal functional score (0.99). R32T had near-normal function by patch clamping (85% of WT peak current and a − 0.1 mV shift in the voltage of half activation; Fig. S10A). Thus, partial loss-of-trafficking *KCNE1* variants can have mild impacts on channel function. 422 of 574 loss- or partial loss-of-function variants (73.5%) did not have reduced cell surface expression (Fig. 4D, Table S8). We refer to these as “gating variants.” These included known gating variants, such as D76N [39].

Using the heatmap of KCNE1 functional scores, we examined regions that were variation-intolerant (i.e., regions that had consistently low scores when mutated to non-WT residues). Variation-intolerant regions were located in the transmembrane helix (residues 61–73) and the transmembrane-proximal cytosolic alpha helix (residues 77–82; Fig. 4F). Common polymorphisms (G38S, D85N) had WT-like functional scores, consistent with their minimal effects on baseline QT interval. Residue 85 is otherwise intolerant of variation and D85N is only one of 2 missense variants at this residue with WT-like score estimates (D85N: 0.79–1.04, and D85E: 0.68–0.98, 95% confidence interval). Variants that disrupted glycosylation sites (residues 5–7 and 26–28) increased cell surface expression in the presence of KCNQ1, but displayed normal function in our functional assay; 60/66 of all missense variants at glycosylation sites had normal functional scores, 2/66 were possible gain-of-function, and 4/66 were possible loss of function (Fig. S9E and F). This appears to be consistent with previous work showing that manipulations to the glycosylation sites have dramatic effects on glycosylation but minimal effects on channel function [42, 43]. Similarly, N-terminal cysteine residues, while gain-of-trafficking, did not drastically affect function in the functional assay. However, a caveat is that the functional assay is not well-powered to detect gain-of-function variants (Fig. 3). Two representative variants (Y107R and C106L), classified as possible gain-of-function in the functional assay, were studied by patch clamp and had gain-of-function properties (Fig. S10B) [42, 43].

The coding region of *KCNE1 *is completely contained within a single exon, 50 bp from the nearest splice site. Nevertheless, we considered potential effects of KCNE1 variants on RNA splicing using the machine learning algorithm SpliceAI (Supplemental file 3) [34]. Of the 1109 investigated single-nucleotide variants affecting the coding region of KCNE1, no variants had SpliceAI scores above the SpliceAI “recommended” cutoff of 0.5, and only 8 variants had SpliceAI scores above “high recall” threshold of 0.2 [34]. These 5 missense and 3 synonymous variants all had SpliceAI scores between 0.2 and 0.4, and thus are moderately predicted to disrupt splicing. One of the candidate splice-disrupting missense variants also had a low functional score (L59Q, estimate − 0.09–0.11, Fig. S10C).

To determine whether KCNE1-HA and KCNQ1-S140G retain a response to beta-adrenergic stimulation, we measured *I*_Ks_ response after addition of forskolin and isobutylmethylxanthin (IBMX), two activators of the adenylyl cyclase-PKA pathway. The compounds had an acute effect of increasing *I*_Ks_ current (Fig. S10D). Therefore, future work could use the framework established in this paper to measure the comprehensive impact of *KCNE1* variants on beta-adrenergic response.

### Structural model of the KCNE1/KCNQ1/calmodulin complex

**Fig. 5:**
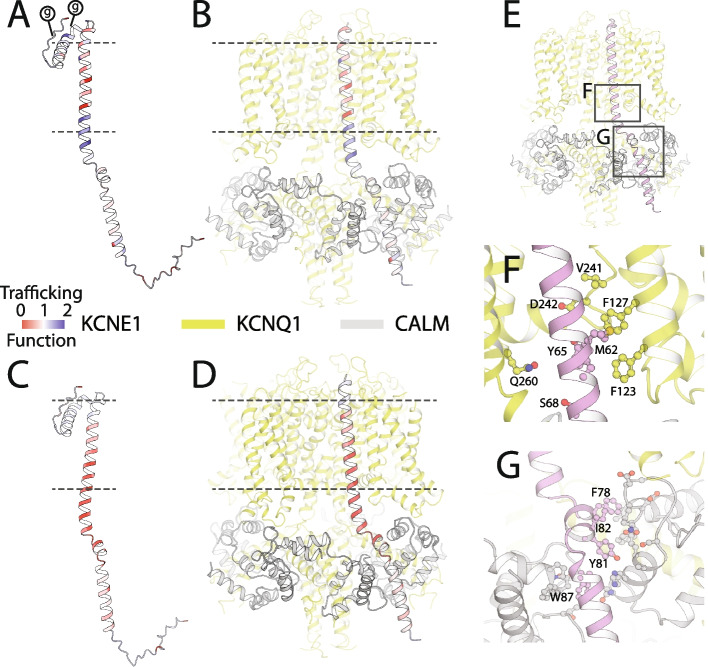
Structural impact of variant effects. **A–D** KCNE1 structural model, coded by mean missense trafficking scores (**A**,**B**) or missense functional scores (**C**,**D**) at each residue on the same color scale as in Figs. 2F and 4F (Red = loss-of-trafficking/function, white = WT-like, blue = gain-of-trafficking/function). Dotted lines indicate the approximate location of the plasma membrane. (*g*) represents glycosylation sites. Panels **B** and **D** show KCNE1 in complex with KCNQ1 (green) and calmodulin (gray). **E** *I*_Ks_ complex showing locations of panels **F** and **G**. **F** Constrained KCNE1 residues (pink) that make contacts with KCNQ1. KCNE1 residue M62 forms a hydrophobic cluster with KCNQ1 residues F123, F127, and V241, whereas KCNE1 residues Y65 and S68 make polar contacts with KCNQ1 residues D242 and Q260, respectively. **G** Constrained KCNE1 residues (pink; F78, Y81, I82, W87) that make contacts with calmodulin. Residues on calmodulin not labeled. A PDB model and and Pymol session files with trafficking and functional scores overlaid on the structure are in Supplemental files 7–8

An experimental structure of the KCNE1/KCNQ1/calmodulin complex has not been determined, so we examined multiple AI-based models (Fig. S11). Aligning predicted structures of KCNE1 to a reported cryo-EM structure of the KCNQ1-KCNE3 complex indicated that the conformation obtained from AlphaFold-multimer modeling (see *Supplemental* *Methods*) was likely the most biologically meaningful (Figs. 1A and 5). We therefore overlaid mean missense trafficking and missense functional scores on the AlphaFold-multimer structural model of the KCNE1-KCNQ1-calmodulin complex (Fig. 5A–D). As discussed above, the hydrophobic core of the transmembrane helix (residues 44 to 60) did not tolerate polar or charged variation in both trafficking and functional maps. Multiple variants in the internal half of the transmembrane domain (residues 61–70) increased cell surface expression. However, these variants were highly intolerant to variation in the functional assay, highlighting that KCNE1 variants can reduce potassium efflux by disrupting either cell surface expression or interactions with KCNQ1. Given the increased cell surface expression of these variants, we hypothesized that variants in this region likely disrupt normal interactions with KCNQ1 and gating properties of the *I*_Ks_ channel. Our hypothesis was supported by examining the *I*_Ks_ channel complex structure, which demonstrated that this region contains extensive contacts with KCNQ1, with 7 of the 9 mutation-intolerant variants within 5 Å of KCNQ1 (Fig. 5F). Polar and charged variation in an additional predicted intracellular helix (residues 85–105 including functionally constrained residues F78, Y81, I82, and W87) preserved cell surface expression. However, the helix was functionally intolerant to variation likely due to observed interaction with calmodulin (Fig. 5G) that affects gating properties of the *I*_Ks_ complex. We identified 30 KCNE1 residues highly intoleract of variation (defined as positions where > 70% of missense variants were loss- or partial loss-of-function). Of these 30 residues, 22 (73%) were in close contact (< 5 Å) with KCNQ1 (14 residues) and/or calmodulin (9 residues; residue 77 was in close contact with both proteins). These variation-intolerant residues comprise 40% of 55 KCNE1 residues in close contact with KCNQ1 and/or calmodulin. On the other hand, none of the 70 residues where > 50% of the missense variants had WT-like functional scores were in close contact with either KCNQ1 or calmodulin.

### Correlation of MAVE scores with patch clamp and patient data

**Fig. 6:**
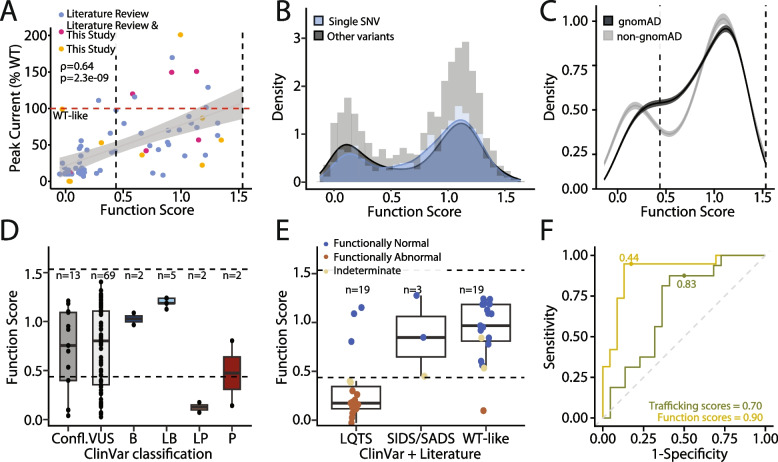
Functional scores correlate with in vitro assays and clinical outcomes. **A** Correlation of function scores with peak current (normalized to WT) from patch clamp studies. Blue: currents obtained from literature review, pink: mean of currents from literature review and this study, yellow: currents measured in this study. **B** Variants generated by single SNVs (blue) are less likely to have low functional scores compared to all other variants (black; *p* = 2.0 × 10^−2^, Wilcoxon Test). **C** Variants present in gnomAD (black) were more likely to have WT-like scores than variants absent from gnomAD (gray; *p* = 3.9. × 10^−3^, Wilcoxon test). Mean and confidence intervals are generated by re-sampling both distributions 100 times. **D** Functional scores by ClinVar classification. **E** Functional scores for presumed benign and presumed pathogenic variants. See Supplemental file 2 for the literature review dataset and Supplemental file 4 for an interactive worksheet with OddsPath calculations. **F** Receiver operator characteristic curves evaluating prediction of variant pathogenicity by functional and trafficking scores. Dots indicate the deleteriousness cutoffs used in this study (functional scores = 0.44, trafficking scores = 0.83). The area under the curve is listed at the bottom of the panel

We curated a list of 149 *KCNE1* variants with previously published patch clamp, trafficking and/or patient data (Supplemental file 2), and conducted additional patch clamp studies of 18 variants (Table S9). The MAVE functional scores were strongly correlated with measured or reported peak currents (*ρ* = 0.64, *p* = 2.3 × 10^−9^, *n* = 71; Fig. 6A). Thirty nine variants with available patch clamp data were partial loss-of-function or loss-of-function in the MAVE assay (functional scores below the cutoff of 0.44). Thirty three of the 39 variants (85%) had a 50% or greater reduction in peak current compared to wildtype (Fig. 6A). Since the patch clamp data was generated with coexpression of WT KCNQ1, these results suggest that most *KCNE1* loss-of-function variants are loss-of-function when coexpressed with either WT or S140G *KCNQ1*. Although functional scores correlated best with peak current, *KCNE1* variants with large shifts in the voltage or kinetics of activation or deactivation were also more likely to have lower scores (Figs. 6B, S12A, and B). We also saw a correlation between functional scores and current measured at −20 mV (*ρ* = 0.65, *p* = 6.4 × 10^−5^; Fig. S12C), though there were fewer variants for this parameter. As expected, the trafficking scores had weaker correlations with patch clamp parameters (Fig. S12D, E, and F). Since transition mutations occur more frequently than transversion mutations, we also investigated the functional effects of transition vs. transversion missense variants. Single-nucleotide variants (SNVs) generated by transversion mutations were more likely to be functionally deleterious compared to variants generated by transition mutations (*p* = 3.4 × 10^−5^, Wilcoxon test; Figs. S12H and I). In addition, we hypothesized that functional scores of variants generated by two or three SNVs would be more likely to have altered scores in our functional assay than those generated by one SNV. Indeed, variants generated by more than one SNV tended to have lower functional and trafficking scores (*p* = 2.0 × 10^−2^ and 3.4 × 10^−5^, respectively, Wilcoxon test; Figs. 6B and S12G). These results are consistent with previous calculations showing that the genetic code is optimized to maximize the chemical similarity of amino acids introduced by transition and single-variant mutations [45].

We examined the distribution of missense variant scores in presumed benign and pathogenic variants, curated from gnomAD, ClinVar, and our literature review of LQT5 and JLNS cases (see “ACMG/AMP assay calibration and comparison,” Table S4 and Supplemental file 1). gnomAD is a genetic resource that aggregates whole exome or whole genome sequence data largely from case–control studies of common adult-onset diseases [14], the vast majority of which do not have long QT syndrome. Thus, we hypothesized that variants absent from gnomAD would be more likely to have deleterious functional scores than variants present in gnomAD. Indeed, variants in gnomAD tended to have lower functional scores (*p* = 3.9 × 10^−3^, Wilcoxon test; Figs. 6C and S13A). Functional scores were largely consistent with clinical outcomes: 7/7 ClinVar benign/likely benign variants and 16/19 presumed benign variants had WT-like functional scores; and 3/4 ClinVar pathogenic/likely pathogenic variants and 16/19 presumed pathogenic variants had loss- or partial loss-of-function scores (Fig. 6D, E). We next tested the performance of our scores against our set of presumed benign and presumed pathogenic variants (see “Methods” for definitions; Table S4). The predictive performance of MAVE scores was tested by estimating true positive and false positive rates across a range of thresholds using receiver operator characteristic (ROC) curves. The area under the ROC curve was 0.90 for functional scores and 0.70 for trafficking scores (Fig. 6F). Using a cutoff for loss-of-function of 0.44, the functional assay had a sensitivity of 94.7% and a specificity of 76.9%. Functional scores also correlated well with several previously validated computational and evolutionary predictors of variant effect, with the highest correlation to AlphaMissense (*ρ* =  − 0.62; Fig. S14 and Table S10). The predictive performance of functional scores was comparable to these metrics (Fig. S13B and Supplemental file 5). We note that while computational metrics perform well for *KCNE1* variants, our MAVE data are derived from direct in vitro data, and thus provide orthogonal information. These trends were weaker for trafficking scores (Figs. S15 and S16).

We examined our assay scores for presumed pathogenic and benign variants to determine the ability of the assay to distinguish pathogenic and benign variants. This process is recommended by the ClinGen Sequence Variant Interpretation working group who propose the term “assay calibration” [17]. We used functional scores for assay calibration because of their better correlation with clinical outcomes and other metrics of disease risk. Using the ClinVar only dataset, the functional assay achieved a likelihood ratio of pathogenicity (termed “OddsPath”) of 5.25 for pathogenic (PS3_moderate) and 0.25 for benign (BS3_supporting). Using the expanded set of presumed benign and pathogenic variants, the functional assay achieved an OddsPath of 14.0 for pathogenic (PS3_moderate) and 0.188 for benign (BS3_moderate).

## Discussion

A majority of variants in clinically relevant genes, including *KCNE1*, are VUS, and resolving the clinical significance of VUS at scale is a major challenge in genomic medicine [11]. High-throughput functional data can be deployed to reclassify as many as 89% of known VUS in clinically actionable genes [16]. Loss-of-function *KCNE1* variants cause type 5 long QT syndrome (LQT5) via two major mechanisms: reduced cell surface expression and/or defective gating (activation of the *I*_Ks _complex) [33]. Our study ascertains trafficking and electrophysiology properties to better understand the disease risk of nearly all possible *KCNE1* variants. Furthermore, our dataset provides functional scores for 871 of the 924 *KCNE1 *variants accessible by a single SNV. Each SNV, if compatible with life, is estimated to exist in approximately 50 humans currently alive [15]. Our comprehensive datasets also reveal new biology not apparent from previous smaller-scale mutational studies.

### Insights from trafficking scores

We identified 470 loss-of-trafficking and 310 gain-of-trafficking missense variants. While we refer to these as “trafficking” scores, we note that our assay actually measures steady state cell surface KCNE1 expression. Variants that disrupt cell surface expression may do so through altered trafficking, or additional processes such as decreased protein translation, increased protein degradation, or altered channel retrograde trafficking. The gain-of-trafficking variants include a stretch of super-trafficking N-terminal cysteine variants; we hypothesize that these residues may increase cell surface expression by forming intermolecular disulfide bonds with other proteins (e.g., KCNE1 or KCNQ1). We tested this hypothesis by conducting a MAVE of cell surface expression in the absence of KCNQ1, but this dataset may be influenced by other low-abundance binding partners of KCNE1 endogenous to HEK293T cells. The trafficking scores in the presence and absence of KCNQ1 for cysteine variants in the extracellular domain were similarly elevated. A comparison of the trafficking scores in the presence and absence of KCNQ1 suggested that loss of N-glycosylation increases cell surface expression in the presence of KCNQ1. This result may indicate that glycosylation disrupts that the interaction between KCNE1 and KCNQ1 but not between KCNE1 and other KCNQx binding partners in HEK293T cells. The latter half of the protein (residues 56–129) was dispensable for cell surface trafficking. Truncated proteins containing only the N-terminal 55-amino acids—including only half of the transmembrane alpha helix—likely still reached the cell surface because they demonstrated cell surface staining of the HA tag in live cells with an intact cell membrane. This transition point for nonsense variants in the trafficking assay is near the FTL motif (residues 57–59), which is important for electrophysiological modulation of the *I*_Ks_channel [46, 47], and corresponds to a previously reported break in the alpha helicity of the transmembrane domain [48, 49].

### Insights from functional scores

We developed a novel selection assay that used a gain-of-function KCNQ1 variant (S140G) to identify loss-of-function KCNE1 variants. With the assay, we identified 574 loss-of-function variants, of which 152 were loss- or partial loss-of-trafficking (26.5%) and the rest had near-normal or increased cell surface expression. We refer to the latter as “gating” variants, as proper K^+^ flux through the *I*_Ks_ channel is likely disrupted. The predominance of pathogenic gating variants is in contrast to other potassium channel disease genes, such as *KCNH2*; 88% of 193 studied KCNH2 loss-of-function variants disrupt cell surface expression [50]. The high proportion of gating variants is likely explained by KCNE1’s primary function as a KCNQ1-activating subunit to modulate the *I*_Ks_ complex. The gating variants identified in our data include well-studied variants like D76N and W87R [39] and novel variants in the cytosolic region adjacent to the transmembrane domain that interact with KCNQ1 and calmodulin. Indeed, most of the variants that were highly mutation-intolerant in the functional assay made close contacts with KCNQ1 or calmodulin (23 of 30 variants). Therefore, although we cannot definitively identify the mechanism of action, we conclude that many of these variants may disrupt function through their interactions with KCNQ1 or calmodulin.

Our work defines three broad classes of nonsense variants based on residue location: #1–55 that disrupt cell surface expression, #56–104 traffic to the cell surface but do not function, and #105–128 have near-normal trafficking and function [51]. We note that to create a functional KCNE1 protein, i.e., a cell surface β subunit that demonstrates wildtype-like behavior in the functional assay, residues 1–104 were needed; this region includes the entire transmembrane domain. While D85N (rs1805128; AF 0.14–2.54% in gnomAD) is associated with modest QT prolongation and fatal arrhythmias in the presence of drugs and other environmental factors [52], the variant is too common to cause long QT syndrome in isolation [53]. The WT-like MAVE score estimates are consistent with this observation (trafficking score: 0.82–1.00; functional score: 0.79–1.02).

One caveat in interpreting our functional scores is that the assay was performed in the context of *KCNQ1*-S140G, not *KCNQ1* WT. Therefore, a *KCNE1* variant may have different properties in the context of these two versions of *KCNQ1*. Despite this difference, we observed high correlations between functional scores measured in the *KCNQ1*-S140G background compared to “gold standard” patch clamp data measured in the WT *KCNQ1* background (peak current *ρ* = 0.64, *p* = 2.3 × 10^−9^). Extrapolating the correlations from the 71 variants with available patch clamp data to the rest of the high-throughput functional dataset, we expect that most of the *KCNE1* loss-of-function variants measured in the *KCNQ1*-S140G background will also cause loss-of-function in the WT *KCNQ1* background.

We observed a complex relationship between *KCNE1* trafficking and functional scores. Nearly all (84/105) variants with loss-of-trafficking had decreased function. However, variants in all other trafficking categories had a range of functional scores (Fig. S9D and Table S8). This result indicates that while KCNE1 trafficking is important, it is not the only factor influencing *I*_Ks_ channel function. *KCNQ1* had a higher expression level than *KCNE1 *at the RNA level in our experiments, so this result may reflect the flexible stoichiometry of the channel, as KCNE1 can bind to KCNQ1 variably in a 1:4 to 4:4 conformation [54]. A similar complex relationship between high-throughput trafficking and functional scores was observed in another cell surface cardiac ion channel gene, *KCNJ2* [55].

cDNA-based assays like ours do not capture defects in protein function due to aberrant splicing. However, while *KCNE1 *has 4 exons in the most common reference isoform [56], the entire protein-coding sequence is in one exon, located 50 base pairs downstream of the nearest splice junction. We therefore predict minimal effects of most *KCNE1* coding variants on RNA splicing. Only 5 missense and 3 synonymous out of 1109 SNVs with normal MAVE scores are moderately predicted to alter splicing. Thus, we urge caution before concluding normal in vitro function for these variants.

### Structural implications of MAVE data

Although there is no solved cryo-EM structure of the KCNQ1-KCNE1-calmodulin complex, we used AlphaFold2 and homology modeling to construct a structural model of the *I*_Ks_ complex. In our MAVE datasets, hydrophilic variants in the hydrophobic transmembrane core (including previously studied variants like L51H) [39] disrupt function by affecting cell surface expression. We also identify a 26 amino acid membrane-proximal cytosolic region (residues 61–87) that comprises essential elements for *I*_Ks_ complex function: KCNQ1-binding and calmodulin-binding regions, and a structured linker connecting the two. Variants in this region likely disrupt hydrophobic and polar interactions and pi-stacking with the corresponding residues of KCNQ1 and calmodulin respectively. Accordingly, the region is highly constrained in the functional, but not trafficking, assay. With increasing confidence in and accuracy of AI-generated structural models, the resolution and interpretation of MAVE-based structure–function relationships will likely improve.

### Clinical associations of deleterious KCNE1 variants

Our dataset also highlights the strength of association between *KCNE1* loss-of-function variants and QT prolongation. In 2020, the ClinGen Channelopathy Working Group assessed *KCNE1 *as having only limited evidence for association with congenital type 5 long QT syndrome (LQT5) and strong evidence for association with acquired long QT syndrome [57]. Since then, several papers have further described the LQT5 risk of *KCNE1* variants. Roberts et al. [1] reported in a multi-center review of LQT5 that *KCNE1* variants are a low penetrance cause of congenital long QT syndrome, i.e., only a small number of mutation carriers develop the long QT phenotype. Our group has previously shown that carriers of pathogenic/likely pathogenic *KCNE1 *variants in the electronic MEdical Records and GEnomics (eMERGE) sequencing study had longer QT intervals and higher odds of arrhythmia diagnoses and phenotypes [58]. However, these variants conferred a lower risk than pathogenic or likely pathogenic variants in *KCNQ1* or *KCNH2* (LQT1 and LQT2). The QT prolonging effects of deleterious *KCNE1 *variants has also been established in both the TOPMed and UK Biobank cohorts, with effect sizes up to 15–20 ms [59]. Thus, *KCNE1* loss-of-function variants appear to contribute to modest increases in baseline QT interval.

All 7 variants with a benign/likely benign classification in ClinVar had normal functional assay scores. Of the 4 variants with a pathogenic/likely pathogenic classification in ClinVar, 3 had loss-of-function assay scores. The inconsistent variant was T7I, which was pathogenic in ClinVar, but had a normal-range assay score of 0.80, above our cutoff of 0.44. T7I was one of two compound heterozygous *KCNE1* variants in the initial report linking *KCNE1 *to Jervell and Lange-Nielsen syndrome (JLNS) [3]. All three JLNS patients had the same genotype (*KCNE1* T7I/D76N) and a specific phenotype (both prolonged QT and congenital deafness) that was used to annotate T7I and D76N as pathogenic. However, there were also 3 T7I carriers (*KCNE1 *T7I/WT) who had a normal QT interval [1, 3]. Two patch clamp studies of T7I showed that it has partial loss-of-function (45 and 41% of WT peak current) [43, 60]. It may be the case that one partial loss-of-function variant (e.g., T7I) and one total loss-of-function variant (e.g., D76N) can together cause JLNS, but that partial loss-of-function *KCNE1* variants do not have a high penetrance for a prolonged QT interval when heterozygous with wildtype (Romano-Ward syndrome).

The presence of a deleterious KCNE1 variant may not be sufficient for development of the congenital LQT5 phenotype due to incomplete penetrance. For the QT interval to rise above a high-risk threshold, LQT5-associated variant carriers may require additional genetic or environmental insults, such as drug exposure or a high polygenic risk score for QT interval [1, 61]. Based on the strong correlation between MAVE scores and functional and clinical outcomes, variants identified as functionally deleterious in this study likely predispose carriers to LQT5. However, due to its small gene size and lower penetrance compared to genes such as KCNQ1 and KCNH2, KCNE1 variants currently appear to be a minor cause of congenital LQTS.

### MAVE data can supplement ACMG/AMP criteria

Both our trafficking and functional assays had excellent separation between early nonsense and synonymous variants, high concordance among replicates, and strong correlations with previous measurements of cell surface trafficking or patch clamp function. In addition, the functional MAVE dataset correlates well with patient and population cohorts, using controls curated from ClinVar, literature reports, and gnomAD. Another advantage of the MAVE approach is the uniformity and replication of the platform used to analyze variants, unlike literature reports derived from multiple individual laboratories with low concordance [62]. Because of this heterogeneity, data from literature reports cannot accurately contribute to ClinGen guidelines for interpreting in vitro functional datasets [17]. The small number of discordant variants between our assay and the literature might represent an error of previous reports or of our assay, which has high but less than 100% sensitivity (94.7%) and specificity (76.9%). Based on the ClinGen guidelines, functional scores can be implemented at the moderate level for both PS3 and BS3 criteria to aid in variant interpretation. However, given the higher clinical burden of false negatives, we recommend using our scores conservatively to apply BS3 at the supporting level. This work adds to the growing set of arrhythmia genes previously assayed by multiplexed assays for either the full protein (KCNJ2, calmodulin) [55, 63] or a portion of the protein (SCN5A, KCNH2) [23, 33, 64].

## Limitations

The MAVE functional assay was not well-powered to detect gain-of-function variants. The scores were generated in a heterologous system that may not fully model the behavior of the *I*_Ks_ channel in a cardiomyocyte. We chose to use HEK cells because of their human origin and an existing landing pad, enabling the rapid integration, selection, and expression of pools of thousands of variants. With advances in genome editing and culturing techniques for induced pluripotent stem cell-derived cardiomyocytes, we are optimistic about their future use for high-throughput functional ascertainment of cardiac ion channel variants. Additionally, we did not model variant effects on behaviors of the *I*_Ks_ channel such as regulation by PIP_2_or beta-adrenergic stimulation, which can be investigated in future studies. We also did not measure dominant negative properties of the channel, demonstrated for some KCNE1 variants including D76N [65]. HEK293T cells endogenously express non-KCNQ1 binding partners of KCNE1 that might confound trends when assessing the trafficking of KCNE1, especially in the absence of KCNQ1. While our functional scores correlated strongly with traditional patch clamp measurements and clinical metrics, some *KCNE1* variants may have different effects on *I*_Ks_ when coexpressed with KCNQ1 wildtype compared to KCNQ1-S140G.

Although KCNE1’s major role in the cardiac action potential is through its role in *I*_Ks_, KCNE1 has also been reported to interact with other proteins including KCNH2 [66] and TMEM16A, a cardiac chloride channel [60]. These interactions might influence arrhythmia risk that is not captured by the *I*_Ks_ assay performed in this study. For example, D85N has been reported to cause a partial loss-of-function of KCNH2 [67], which may help explain its influence on the QT interval. An extension of this work would be to investigate the influence of comprehensive KCNE1 variant libraries on other protein complexes.

## Conclusions

We comprehensively ascertained variant function in an important arrhythmia gene, *KCNE1*. We identified 470 variants that affect KCNE1 trafficking and 574 variants that alter function. Our work highlights new biology of the *I*_Ks_ channel and can be implemented in the ACMG/AMP scheme to classify variants. Only a very small fraction of the world population has been sequenced for *KCNE1*, so most of the 574 putative loss- or partial loss-of-function *KCNE1* variants have not yet been observed. As genetic testing becomes more prevalent, we expect that most of these 574 variants will be detected.

### Supplementary Information

**Additional file 1:** ClinVar Classifications of KCNE1 variants.

**Additional file 2:** Literature review of KCNE1 variants.

**Additional file 3:** SpliceAI scores for single SNV variants tested in this study.

**Additional file 4:** Interactive worksheet used to calculate “OddsPath” scores.

**Additional file 5:** Computational scores curated for variants tested in this study.

**Additional file 6:** Functional and trafficking scores for KCNE1 variants.

**Additional file 7:** AlphaFold2 structure of the KCNE1/KCNQ1/calmodulin complex.

**Additional file 8:** A zipped folder containing .pse Pymol session files corresponding to Figs. 1A and 5B, C, D, F, and G.

**Additional file 9.**

## Data Availability

The three MAVE datasets supporting the conclusions of this article are available in Supplemental file 6 and in MaveDB ( https://www.mavedb.org/#/experiments/urn:mavedb:00000674-a) [68]. Illumina sequence data are available in the Short Read Archive (https://www.ncbi.nlm.nih.gov/bioproject/?term=PRJNA1103764). Code to analyze MAVE data and re-create figures is available at https://github.com/GlazerLab/KCNE1_DMS.
