# Supplementary material for: High-throughput functional mapping of variants in an arrhythmia gene, KCNE1, reveals novel biology
Source: Genome Med. 2024 May 30;16:73. doi: 10.1186/s13073-024-01340-5 (PMC11138074; doi:10.1186/s13073-024-01340-5)
Supplement: Supplementary file 9 — Additional file 9. [file 13073_2024_1340_MOESM9_ESM.docx]

**High-throughput functional mapping of variants in an arrhythmia gene, *KCNE1*, reveals novel biology**

**Authors:** Ayesha Muhammad, Maria E. Calandranis, Bian Li, Tao Yang, Daniel J. Blackwell, M. Lorena Harvey, Jeremy E. Smith, Zerubabell A. Daniel, Ashli E. Chew, John A. Capra, Kenneth A. Matreyek, Douglas M. Fowler, Dan M. Roden,* and Andrew M. Glazer*

**Online Supplement**

Table of Contents

[Supplemental Methods 4](#_Toc164279486)

[Supplemental Figures 11](#_Toc164279487)

[Fig. S1: Overview of multiplexed assays of variant effect in this study 11](#_Toc164279488)

[Fig. S2: Plasmid maps, flow cytometry gates, and sequencing procedures in the study 12](#_Toc164279489)

[Fig. S3: Creation, diversity, and coverage of the KCNE1 variant library 13](#_Toc164279490)

[Fig. S4: Flow cytometry gates for sorting cells based on cell surface KCNE1 expression 14](#_Toc164279491)

[Fig. S5: Quantification of KCNE1-HA protein at the cell surface by flow cytometry 15](#_Toc164279492)

[Fig. S6: Inter-replicate distributions of KCNE1 trafficking scores (+KCNQ1) 16](#_Toc164279493)

[Fig. S7: Cell surface expression heatmap for KCNE1 library (-KCNQ1) 17](#_Toc164279494)

[Fig. S8: Development of functional assay and distribution of functional scores 18](#_Toc164279495)

[Fig. S9: Relationship of functional and trafficking variant scores and validation of gain-of-function variants 19](#_Toc164279496)

[Fig. S10: Patch clamp data and splicing evaluation for individual variants 20](#_Toc164279497)

[Fig. S11: Comparison of structural models of full-length KCNE1 from various structure prediction methods and NMR 21](#_Toc164279498)

[Fig. S12: Correlation of functional and trafficking scores with electrophysiological parameters and genomic variant categories 22](#_Toc164279499)

[Fig. S13: Distribution of functional scores by gnomAD category and MAVE data prediction performance 23](#_Toc164279500)

[Fig. S14: Spearman correlation of functional scores to previously developed computational predictors of protein function and evolution 24](#_Toc164279501)

[Fig. S15: Correlation of trafficking scores to population and clinical case/control data 25](#_Toc164279502)

[Fig. S16: Spearman correlation of trafficking scores to previously developed computational predictors of protein function and evolution 26](#_Toc164279503)

[Fig. S17: Convergence of the docking calculation of KCNE1 to KCNQ1 and calmodulin complex 27](#_Toc164279504)

[Supplemental Tables 28](#_Toc164279505)

[Table S1: Primers used in this study 28](#_Toc164279506)

[Table S2: Primers for Jain mutagenesis for *KCNE1* variant library generation 32](#_Toc164279507)

[Table S3: Primers used for each Illumina library prepared in this study 36](#_Toc164279508)

[Table S4: List of presumed pathogenic and presumed benign variants included in the population level analyses 37](#_Toc164279509)

[Table S5: Number of variants in each trafficking category 38](#_Toc164279510)

[Table S6: Expression levels of *KCNQ1* and *KCNE1* by RNAseq 39](#_Toc164279511)

[Table S7: Number of variants in each functional score category 40](#_Toc164279512)

[Table S8: Relationship between trafficking and functional score categories for missense variants 41](#_Toc164279513)

[Table S9: Patch clamp data generated in this study 42](#_Toc164279514)

[Table S10: List of previously developed computational predictors compared to MAVE scores in this study 43](#_Toc164279515)

[References 44](#_Toc164279516)

# Supplemental Methods

***Modifying pIRES2 to have a NotI restriction site***

Using QuikChange mutagenesis, we modified the pIRES2-dsRED2 and pIRES2-EGFP mammalian expression constructs (Clontech) in two ways. We introduced a NotI restriction site in the multiple cloning site (MCS) and removed a NotI site outside the MCS. These plasmids were used for cloning *KCNE1* or *KCNQ1* cDNA into the MCS using NotI restriction digestion for expression studies.

***Subassembly of the variant library***

To subassemble the library, i.e., associate each 18-mer barcode with its specific *KCNE1* variant, the library was divided into 9 regions as shown in Fig. S2D. The barcode was located 110 bp upstream of KCNE1 in the plasmid, so PCR primers were designed to amplify both the barcode and selected regions of the KCNE1 gene. Each region was sequenced on the Illumina NovaSeq platform using paired-end 150 base pair sequencing to give at least 100 million reads per region. The barcodes were extracted from reads using custom Python and Unix scripts. Each read included in the downstream analyses was required to have the correct anticipated 6 base prefix and 6 base suffix surrounding the barcode. Barcodes with over 500 total reads of subassembly data were retained, and barcodes were required to differ by at least 2 SNVs from each other. 99,418 candidate barcodes met these criteria (Fig. S2E). The reads associated with each barcode were aligned to the reference *KCNE1*-HA plasmid sequence using the Burrows-Wheeler Alignment (bwa) tool and variants were called using samtools.^1,2^ Any barcodes associated with WT constructs, variants within the HA tag, multiple *KCNE1* variants, insertions or deletions were excluded from all analyses, resulting in 80,282 “good” barcodes each corresponding to a single nonsense, missense, or synonymous variant out of 2,592 unique protein variants.

***In-dish cell staining***

Cells in replicate 1 of the *KCNE1* with *KCNQ1* cell surface trafficking experiment were stained while adhered to the dish as follows. Cells were washed with OptiMEM (Thermo Fisher), before being stained in dishes for 30 min with 1:500 anti-HA Alexa Fluor 647 antibody (Cell Signaling, #3444) dissolved in DMEM. The cells were washed with HEK media 3 times, and then briefly with OptiMEM. Next, the cells were incubated with 0.5 mM EDTA in PBS (Corning) for 7 minutes at room temperature and resuspended in DMEM. All other steps were identical to the "in-suspension" staining described in the Methods section. Since the trafficking scores from this replicate were highly concordant with those from the other two replicates where cells were stained in suspension (Fig. S6B), the scores from all three replicates were combined and averaged as described below.

***Trafficking score calculation***

For each sample, custom Python, R, and Unix scripts were used to process reads and calculate trafficking scores as follows. Reads that matched the expected 6-base sequences before and after each barcode were retained and aggregated. Only barcodes in the list of 80,282 “good” barcodes from the subassembly (see above) were used for subsequent analyses.

The barcode counts were normalized to the total number of reads in each sample to calculate a "reads per million" metric according to the equation below:

$$F_{i,j}= \frac{R_{i,j}}{T_{j}} \times{10}^{6}$$

Where *i* is the barcode ID, *j* is the bin number (1 through 4, Figs. S4A and S4B), *R_i,j_* is the number of raw reads for barcode *i* in bin *j*, and *T_j_* is the total number of reads in bin *j*.

Next, the read counts were aggregated by variant:

$F_{v,j}= \sum_{i=1}^{barcode} F_{i,j}$

Where *i* is the barcode ID corresponding to variant *v*, *j* is the bin number (1 through 4), *F_v,j_* is the frequency of the variant *v* in bin *j*, and *F_i,j_* is the frequency of barcode *i* in bin *j*.

A weighted average was then calculated for each variant (*W_v_*) as follows:

$W_{v}= \frac{{0\times F}_{v,bin 1}+ {1\times F}_{v, bin 2}+{2\times F}_{v, bin 3}+{3\times F}_{v, bin 4}}{F_{v,bin 1}+ F_{v, bin 2}+F_{v, bin 3}+F_{v, bin 4}}$

Where cells expressing variants with lowest and highest cell surface anti-HA labeling are in bins 1 and 4 respectively.

Next, the weighted averages were linearly transformed so that synonymous variants would have a median score of 1, and trafficking-null nonsense variants would have a median score of 0. This way, the normalized scores could be biologically interpreted: for example, a variant with a score of 0.5 would traffic approximately half as well to the cell surface compared to WT. Since we observed that the nonsense mutations followed a bimodal distribution, with a drastic shift in scores at residue #56, this normalization of raw scores was based on the distribution of synonymous and early (i.e., before residue 55) nonsense variants, as follows:

$$S_{v}= \frac{W_{v}-Median(W_{v,syn})}{Median\left( W_{v,syn} \right)-Median(W_{v,early nonsense})}$$

Where for variant *v*, *S_v_* is the normalized score, *W_v_* is the weighted average across the 4 bins, and *W_v,syn_* is the set of weighted averages of all synonymous variants represented in the library. *S_v_* for each variant was calculated across each replicate. For quality control (QC), the frequency of each variant across each replicate experiment was calculated as follows:

$$F_{v,tot}= F_{v,bin 1}+ F_{v, bin 2}+F_{v, bin 3}+F_{v, bin 4}$$

For different cutoffs of *F_v,tot_*, the coefficient of variance (CV) for the distribution of synonymous variants was calculated across each replicate as follows:

$$CV=\frac{std dev(S_{v,syn})}{mean(S_{v,syn})}$$

For each replicate, we plotted the CVs, the mean scores of the synonymous distribution, and the number of unique missense variants against different cutoffs of *F_v,tot_* (Fig. S6A). The threshold for *F_v,tot_* to minimize the CV and maximize the number of unique missense variants was determined to be 100 for each replicate for downstream analyses. Accordingly, variants with a total frequency lower than 100 in each replicate were dropped from trafficking score calculations. 39/2,592 variants did not meet this frequency threshold. For each variant meeting this QC threshold, the normalized scores *S_v_* were averaged across 3 replicates and standard error of the mean calculated. Variants were divided into 6 categories: loss-, partial loss-, possible loss-, normal, possible gain-, and gain-of-trafficking as defined in the main methods and in Table S5.

For ACMG-AMP assay calibrations, we used a simplified classification system. Variants with confidence intervals below 0.83 were considered “loss-of-trafficking,” variants with confidence intervals above 0.83 and below 1.18 were considered “normal,” and variants with confidence intervals above 1.18 were considered “gain-of-trafficking.”

***Functional Score Calculation***

To calculate functional scores, custom Python, R, and Unix bash scripts were used to process reads for each replicate, where each replicate was harvested on Days 0, 8 and 20. Reads were filtered to remove mismatches in the expected 6-base region before and after the barcode position and aggregated by barcode. Only barcodes found in the list of 80,282 “good” barcodes from the subassembly were used for downstream analyses as described above.

Similar to the trafficking score calculation, barcode counts were normalized to the total number of reads in each sample to calculate a "reads per million" metric according to the equation below:

$$F_{i,d}= \frac{R_{i,d}}{T_{d}} \times{10}^{6}$$

Where *i* is the barcode ID, *d* is the day number (0, 8 or 20), *R_i,d_* is the number of raw reads for barcode *i* in the sample harvested on day *d*, and *T_d_* is the total number of reads in the sample harvested on day *d*.

Next, the read counts were aggregated by variant:

$F_{v,d}= \sum_{i=1}^{barcode} F_{i,d}$

Where *i* is the barcode ID corresponding to variant *v*, *d* is the day number (0, 8 or 20), *F_v,d_* is the frequency of the variant *v* in the sample harvested on day *d*, and *F_i,d_* is the frequency of barcode *i* in the sample harvested on day *d*.

Next, a log_2_ transformed ratio of the frequency of each variant on day 0 compared to day 20 was calculated (*R_v,20_*) as below. We added 1 to the numerator and denominator to avoid dividing by 0.

$$R_{v,20}={log}_{2}\left( \frac{F_{v,20}+1}{F_{v,0}+1} \right)$$

A similar ratio (*R_v,8_*) representing the functional scores on day 8 was also calculated. Next, day 8 and day 20 scores were linearly transformed as above. Synonymous variants would have a median score of 1, and trafficking-null early nonsense variants would have a median score of 0. 54/2,592 variants with less than 30 reads at day 0 of the functionassay (*F_v,0_*) were dropped from score calculations.

Heatmaps were generated using the R package *heatmap.2*^3^ and ROC curves were generated using *plotROC*.^4^

Variants were divided into 6 categories: loss-, partial loss-, possible loss-, normal, possible gain-, and gain-of-function as defined in the main text and Table S7.

For ACMG-AMP assay calibrations, we used a simplified classification system. Variants with confidence intervals below 0.44 were considered “loss-of-function,” variants with confidence intervals above 0.44 and below 1.53 were considered “normal,” and variants with confidence intervals above 1.53 were considered “gain-of-function.”

***Function assay — control variants***

To determine the degree of selection against control variants, a 1:1 ratio of single *KCNE1* variant and non-*KCNE1* containing AttB plasmids was integrated into the landing pad of LP-KCNQ1-S140G cells as described in the Methods and Fig. 3B. During initial plasmid integration and selection for successful integration events, cells were grown in 500 nM HMR 1556 (Tocris) to inhibit I_Ks_. The KCNE1^-^ plasmid consistently results in a lower red fluorescence level than the KCNE1^+^ plasmids and allows for distinguishing between cell populations. After 8 days to enrich for successfully integrated cells, HMR 1556 was removed from cell media to allow K^+^ flux-based selection (Day 0). Cells were grown and passaged as described above. The ratio of KCNE1^-^ (low dsRed) to KCNE1 variant (high dsRed) cells was serially measured by flow cytometry (LSR Fortessa SORP, BD Biosciences). These ratios were then normalized to the ratio at Day 0 corresponding to HMR 1556 removal. This experiment was performed with 3 replicate samples per variant. The mean and standard error of the three replicate measurements was calculated for each data point.

***Cell staining for manual flow cytometry validation and microscopy***

Several previously studied variants were examined by flow cytometry and confocal microscopy to validate the trafficking assay (Figs. 1D and 1E). HEK293T cells were transfected with p*KCNQ1*:IRES2:dsRED2 and p*KCNE1*-HA:IRES2:dsRED2 using Lipofectamine 2000. 48 hours later, transfected cells were harvested using Accutase, washed in block (1% FBS +25 mM HEPES (Sigma, pH 7.0) in PBS without Ca^2+^/Mg^2+^), and incubated for 30 minutes with the anti-HA Alexa Fluor 647 (AF647) antibody. If used for flow cytometry, the cells were washed three times and analyzed on the LSRFortessa SORP (BD Biosciences). If used for microscopy, the cells were washed, fixed in 4% paraformaldehyde (Thermo Fisher), and permeabilized in 0.2% SDS (Sigma, #L4390) for 5 minutes. Cells were stained with anti-HA Alexa Fluor 488 (AF488) antibody (Cell signaling, #2350), washed and stained with 1:1000 Hoechst, and resuspended in 50% glycerol before being added to cover slips. Images were acquired on an inverted confocal microscope (Olympus) equipped with a spinning disk (Yokogawa), 60x silicone objective, and ORCA-Fusion CMOS camera (Hamamatsu). Excitation was achieved using 100 mW solid-state diode lasers. Hoechst was excited at 405 nm, AF488 at 488 nm, dsRED2 at 561 nm, and AF647 at 640 nm. 16-Bit images were acquired sequentially and exported to ImageJ (FIJI) for analysis. Images are shown with equivalent intensity scaling after background subtraction.

***Literature Review (additional information)***

Variants with an AF >0.1% in gnomAD were excluded from the literature review. For variants reported in multiple papers from the same site or with overlapping authors, we included patient data only from manuscripts with the largest number of reported patients to prevent double counting. Patient phenotype annotations were as reported in the manuscripts (i.e., we did not attempt to re-adjudicate the phenotype if a patient was annotated as having long QT syndrome). If only a QT interval without assertion of either long QT syndrome or unaffected carrier was given, we used a QTc of ≥460 ms to determine LQT5, as per a large *KCNE1* patient curation study.^5^ For patch clamp data, the peak current densities of *KCNE1* variants coexpressed with *KCNQ1* (normalized to WT) were collected. When possible, the current density at +60 mV was used, however other voltages (e.g., +40 mV) were used if those were the only data reported. In addition, we curated voltage of half activation (V_½_) and the time constant (τ) of deactivation (each normalized to WT). Some papers reported *KCNE1* variants that, when co-expressed with *KCNQ1*, had KCNQ1 only-like currents (i.e., complete loss of KCNE1 function) but did not quantify peak current. These variants were annotated as having a normalized peak current of 10% (the typical approximate peak current of KCNQ1 only compared to KCNQ1+KCNE1). For variants with multiple papers reporting *in vitro* patch clamp data for a variant, these data were averaged. For most literature reports, there is no record of whether the WT allele was S38 or G38. However, S38G does not substantially alter properties of the I_Ks_ channel^6^ and does not affect the QT interval in large, well-powered GWAS studies,^7^ and is therefore unlikely to significantly affect mutation results.

***Modeling KCNE1 structure with AlphaFold2***

We leveraged multiple deep-learning protein structure prediction algorithms (ESMFold,^8^ trRosetta,^9^ and AlphaFold2^10^) to generate tertiary structure models of KCNE1. The AlphaFold KCNE1 structural model was constructed through the AlphaFold Google Colab notebook, a slightly simplified version of the full AlphaFold2 algorithm.^11^ We ran the Colab notebook with default parameters using the full-length KCNE1 amino acid sequence only (UniProtKB: P51382) as well as both the full-length sequences of KCNQ1 (UniProtKB: P51787 isoform 1) and KCNE1, i.e., AlphaFold-Multimer.^12^ The latter was to test the hypothesis that KCNQ1 might influence KCNE1 conformation upon binding of the two proteins. It has been noted that the Colab notebook has a small drop in average accuracy for multimers compared to local AlphaFold installation.^13^ We compared the structures to each other and to a previously resolved NMR structure (PDB ID: 2K21; Fig. S11).^14^

Comparing the resolved cryo-EM structure of KCNQ1/KCNE3 to the predicted models of KCNE1, the AlphaFold-Multimer model appeared to be the most biologically meaningful, and thus all further analyses used this model. We modeled the structure of KCNE1 in complex with KCNQ1 and calmodulin via protein-protein docking using the Rosetta macromolecular modeling software suite.^15,16^ Briefly, we first energy-minimized AlphaFold2 predicted KCNE1 structure with respect to the RosettaMembrane energy function.^17^ We then prepared the starting pose of KCNE1 with respect to tetrameric KCNQ1 by aligning energy-minimized KCNE1 structure to KCNE3 in the KCNQ1-KCNE3-calmodulin cryo-EM structure (PDB ID: 6V00)^18^ and removing the KCNE3 subunits after aligning. AlphaFold2 predicts a “low” to “very low” confidence flexible linker between the extracellular alpha helix and the transmembrane domain. This flexible linker causes KCNE1 residues 1-30 to clash with the transmembrane domains of KCNQ1 in the homology model. Thus, we removed residues 1-30 from the N terminus of KCNE1. In protein-protein docking, we treated KCNE1 as the moving component and set the values of perturbation flags as -dock_pert 0.3 0.8, -dock_mcm_trans_magnitude 0.1, and -dock_mcm_rot_magnitude 1.0 to enable more local sampling. We generated 5000 models of KCNE1 in complex with KCNQ1 and calmodulin with good convergence (Fig. S17) and selected the model with the best interface score for structural analysis in this work. Models of the I_Ks_ complex with mean function and trafficking scores overlaid are given in Supplemental File 8.

***Computational and evolutionary metrics***

We tested the performance of MAVE scores against previously published and validated computational metrics (Table S10).^19–35^ These consisted of metrics that assess variant deleteriousness on protein function and/or evolutionary conservation of the residues. These scores for each variant in KCNE1 were either obtained from dbNSFP version 4.0a,^36,37^ a database of functional prediction and annotation of all nonsynonymous SNVs, or calculated as previously described. A curated list of computational scores is provided in Supplemental File 5.

***Whole-cell voltage clamping:***

Electrophysiological functional studies were performed at room temperature (22 - 23˚C) using a patch-clamp system: MultiClamp 700B amplifier, 1350 DigiData and data acquisition software pClamp 10.7 (Molecular Devices Inc., Sunnydale, CA, USA). Patch glass microelectrodes with 3~5 mΩ were used to patch cells. The pipette (intracellular) solution contained (in mmol/L) KCl 110, MgCl_2_ 1.0, ATP-K_2_ 5.0, BAPTA-K_4_ 5.0, and HEPES 10 with the pH of 7.2, adjusted with KOH. The extracellular solution contained (in mmol/L) NaCl 145, KCl 4.0, MgCl_2_ 1.0, CaCl_2_ 1.8, glucose 10, and HEPES 10 with the pH of 7.4, adjusted with NaOH. Data acquisition was performed using pClamp 10.7, sampling at 1 kHz and low-pass-filtered at 5 kHz. Activating current was elicited with 5-second depolarizing pulses from a holding potential of −80 mV to +60 mV at 20-mV increments, and deactivating tail current was recorded upon return to −40 mV for another 5 seconds. The voltage-clamp protocols used are shown in Fig. S10. Pulses were delivered every 30 seconds. Data were analyzed by using Excel and current-voltage (I-V) relationship curves for activating I_Ks_ in different groups of cells were generated by using the software version Origin Pro 8.5.1 (OriginaLab Corp., MA, USA). Current densities (pA/pF) were obtained after normalization to cell surface area calculated by the function Membrane Test in pClamp 10.7.

To measure beta-adrenergic response, HEK293 cells expressing KCNE1-HA and KCNQ1-S140G were measured using voltage clamp with repeated pulses (every 15 s) from a holding potential of -80 mV to +60 mV for 5s, followed by a 5s hold at 40 mV. Two activators of the adenylyl cyclase-PKA pathway, 10 μM forskolin (Sigma) and 200 μM IBMX (isobutylmethylxanthin; Sigma) were added to the cells. *I*_Ks_ response to the compounds was then measured.

***RNA sequencing***

HEK293 cells were harvested with accutase (Gibco), resuspended in HEK media, pelleted, and

stored in Qiagen RLT buffer. RNA was prepared using an RNeasy kit (Qiagen). Stranded mRNA

poly-A RNAseq libraries were prepared by the Vanderbilt Technologies for Advanced Genomicscore facility (VANTAGE). 150 base paired-end sequencing was performed on an Illumina NovaSeq 6000 instrument (approximately 25 million reads/sample). Fastq files containing the reads were cleaned (adapter trimming and quality filtering) using fastp version 0.19.4.^38^ Reads were mapped to the hg19 genome with HISAT2 version version 2.2.1.^39^ hg19 was chosen due to a known error for KCNE1 in the genome assembly for hg38 (an incorrect duplication of KCNE1^40^). Alignments were assembled with stringtie version 2.1.1.^41^ FPKM was quantified against the hg19 genome using ballgown in R version 4.0.2.^42^ KCNE1 and KCNQ1 Fragments Per Kilobase Mapped (FPKMs) were averaged across replicate samples and a standard error of the mean was calculated in R.

# Supplemental Figures

## Fig. S1: Overview of multiplexed assays of variant effect in this study

Comprehensive KCNE1-HA variant libraries were integrated into either LP-KCNQ1 cells (trafficking assay), LP cells (trafficking assay in the absence of KCNQ1) or LP-KCNQ1-S140G cells (functional assay). Cells for the trafficking assays were stained with anti-HA antibody and flow sorted into four groups based on KCNE1 cell surface expression. Each group was deep sequenced to quantify variant presence in each group for trafficking score calculations (See Supplemental methods). Cells for the functional assay were grown for 20 days and samples at Day 0, Day 8 and Day 20 were deep sequenced to calculate functional scores. Different colors of cells represent example variants with different KCNE1-HA surface expression or function (green: loss of trafficking or function, blue: low trafficking or function, gray: normal trafficking or function, yellow: high trafficking or function).


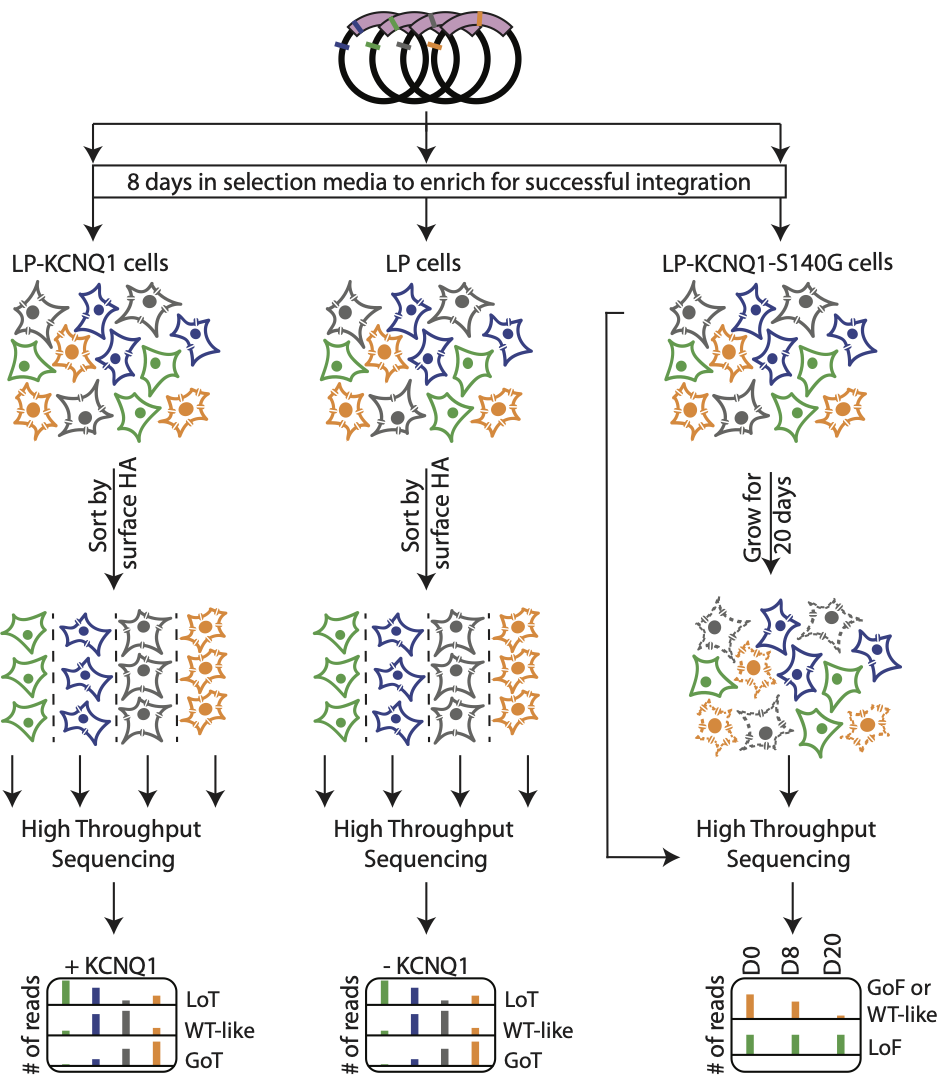


## Fig. S2: Plasmid maps, flow cytometry gates, and sequencing procedures in the study

A) Three different plasmids, constitutively expressing *KCNE1* (left), landing pad-compatible AttB-*KCNE1*-mCherry-BlastR (middle), and non-expressing minimalist *KCNE1* (right) were used for cloning in the study. B-C) Representative fluorescent marker expression and gating to select for LP-KCNQ1 cells during development (B) and single colony screening (C). D) Primers used to sequence different portions of *KCNE1* and the corresponding barcode for subassembly. E) Histogram of barcode frequency during subassembly. Variant assignment was attempted for barcodes with >=500 reads (red line). Barcodes below this threshold were discarded.


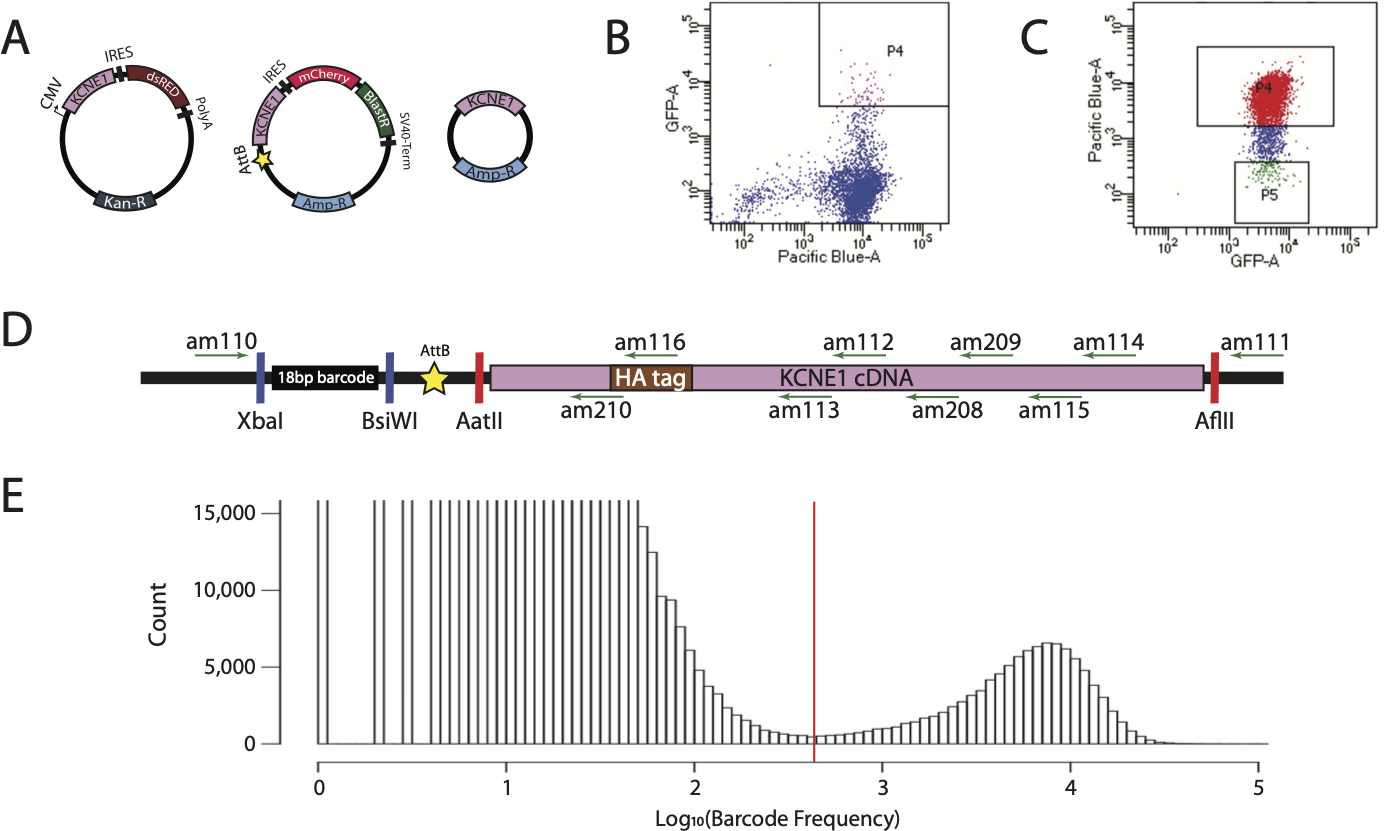


## Fig. S3: Creation, diversity, and coverage of the KCNE1 variant library

A) Creation of the KCNE1 variant library. Saturation PCR mutagenesis using degenerate NNK primers on a small, promoterless plasmid template was used to generate the variant library. The library was then subcloned with restriction digestion into the AttB landing pad compatible plasmid backbone. A poly-N 18 base barcode was added to the AttB plasmid by restriction digestion. B) Number of variants present subassembled per KCNE1-HA residue (max = 21: 19 missense, 1 synonymous, and 1 nonsense). The HA tag (not targeted during mutagenesis) had 2 off-target variant barcodes; these barcodes were discarded. C) Mean number of barcodes per variant at each KCNE1 residue. The ribbons represent the standard deviation. The HA tag is not represented in this panel.

**
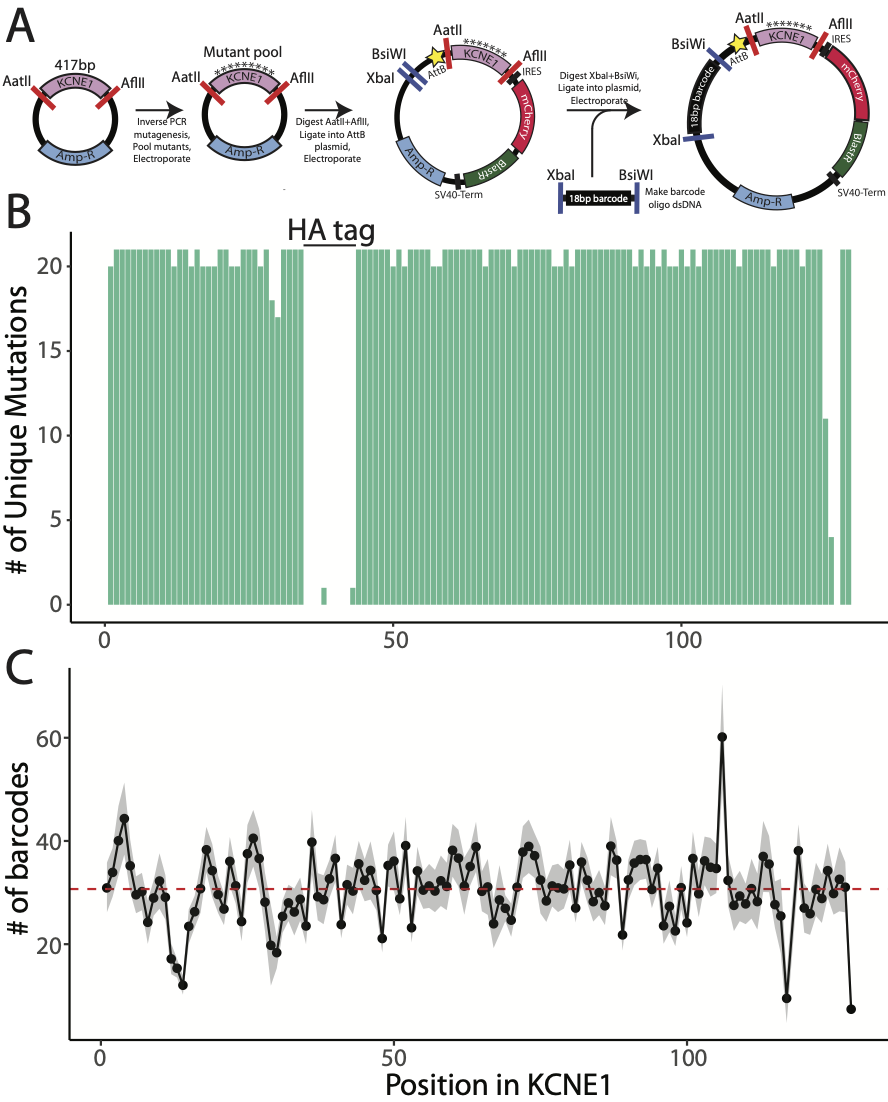
**

## Fig. S4: Flow cytometry gates for sorting cells based on cell surface KCNE1 expression

Flow cytometry gates for +KCNQ1 (A) and -KCNQ1 (B) trafficking experiments.


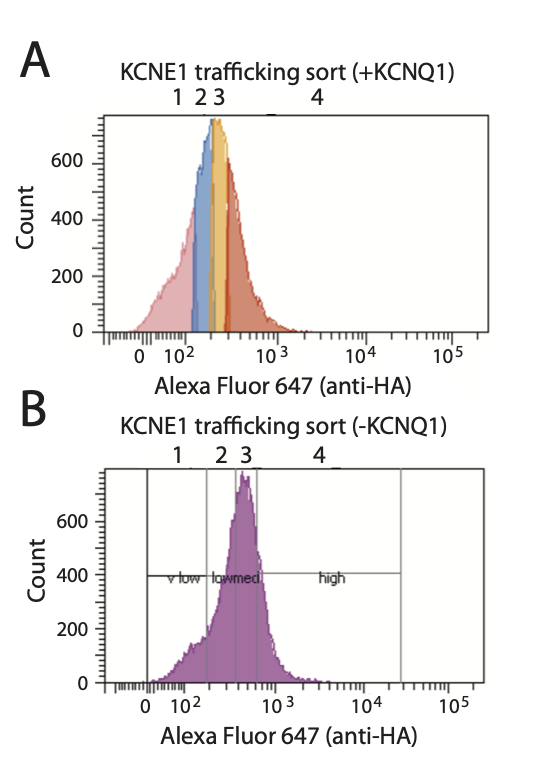


## Fig. S5: Quantification of KCNE1-HA protein at the cell surface by flow cytometry

A) Comparison of anti-HA labeling of two KCNE1 HA tags in alternate positions coexpressed with KCNQ1. An HA tag cloned between KCNE1 residues 34 and 35 (pink, this study) labeled approximately 3-fold more strongly with an anti-HA Alexa Fluor 647-conjugated antibody than a previously-studied HA tag between residues 22 and 23 located in a predicted alpha helix (purple).^43^ B) Cell surface abundance of KCNE1-HA (34-35) for WT and previously studied variants. Data are from the same experiment in Fig. 1E. Three independent replicates for each sample were transfected and stained. Blue = KCNE1-HA variants integrated into LP cells; Red = KCNE1-HA variants integrated into LP-KCNQ1 cells. Cells were stained with an Alexa Fluor 647-conjugated anti-HA antibody. For each sample, the median Alexa Fluor 647 value across all cells is plotted. Bars show the mean of the 3 replicate values ± standard error. All values are normalized to the mean values for empty vector controls (0%) and KCNE1-HA-WT + KCNQ1 (100%). Co-expression of KCNQ1 results in a 6.1-fold increase in KCNE1-HA-WT cell surface abundance, but variant effects were similar in the presence and absence of KCNQ1.


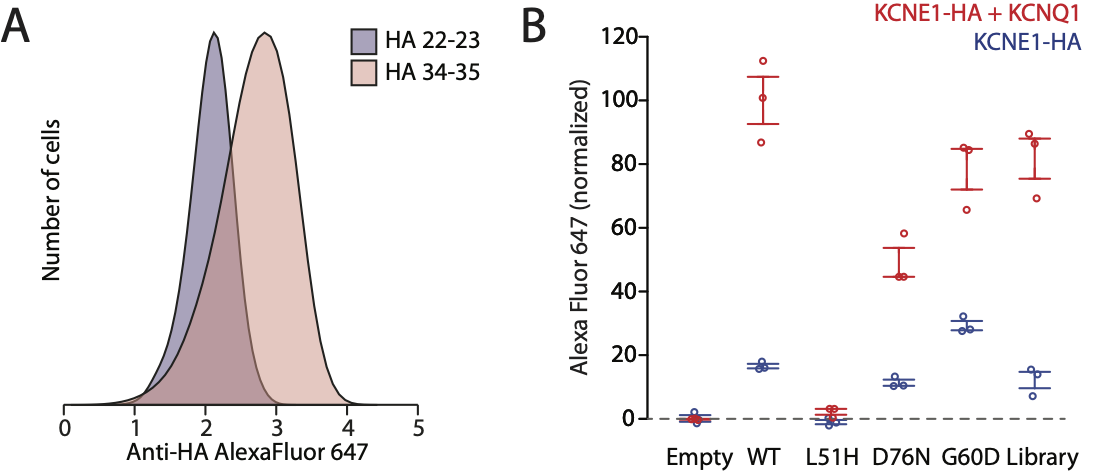


## Fig. S6: Inter-replicate distributions of KCNE1 trafficking scores (+KCNQ1)

A) Quality control prior to trafficking score calculation across three replicates. The coefficient of variation of the synonymous distribution (top), and the mean and median (middle, black and red respectively) synonymous variant scores as a function of total variant frequency, *F_v,tot_*, across each replicate experiment. The number of unique missense variants that passed each variant frequency cutoff (bottom). A cutoff of 100 (dotted line) was determined to minimize the coefficient of variation and maximize the number of unique missense variants for which scores were calculated. Variants with less than 100 reads per million in any replicate were excluded. B) inter-replicate Spearman correlation of trafficking scores based on variant category. ***: p-value < 0.001; **: p-value < 0.01; *: p-value < 0.05. For the trafficking scores, early and late nonsense variants are defined as variants at residue 1-55 and 56-129 respectively. C) Trafficking score correlations against reported measurements of variant cell surface expression. See Supplemental File 2 for the literature review dataset. D) Manual validation of R98W and G52R showed partial and complete loss of cell surface expression, respectively. E) Trafficking score correlations against reported and manually validated measurements of variant cell surface expression.

**
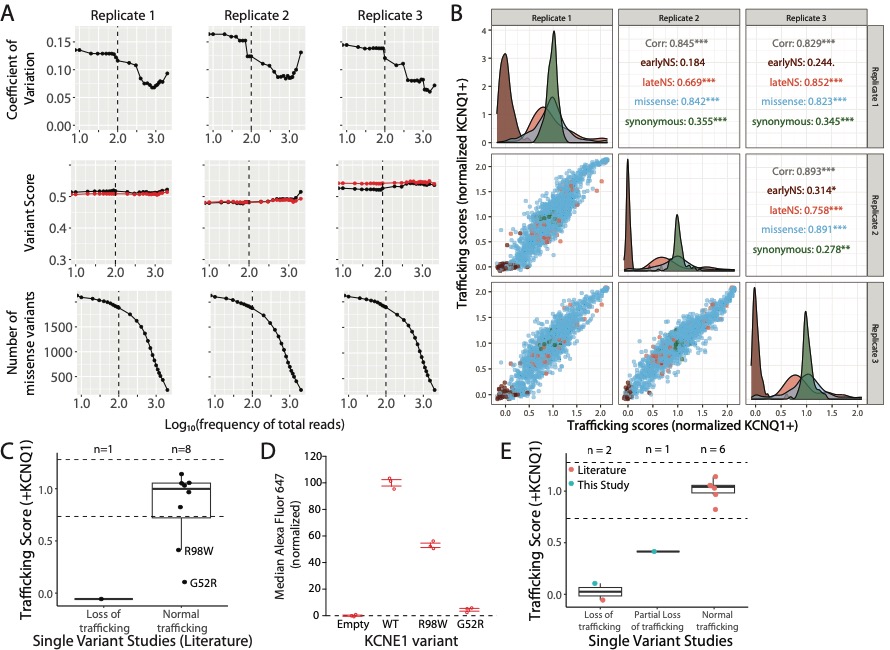
**

## Fig. S7: Cell surface expression heatmap for KCNE1 library (-KCNQ1)

A) Multiplexed assay of KCNE1 variant trafficking as seen in Fig. S1. A comprehensively mutated barcoded plasmid pool was integrated into LP-KCNQ1 (“+KCNQ1”; panels B-C) or LP cells (“-KCNQ1”, panels D-F). Cells were stained and sorted by surface HA and deep sequenced. Different colors of cells represent three example variants with different KCNE1-HA surface expression (green: loss of trafficking, gray: normal trafficking, yellow: high trafficking). B,E) KCNE1 trafficking heatmaps. Red, white, and blue indicate loss-of-trafficking, normal trafficking, and gain-of-trafficking, respectively. WT amino acids are indicated at each position with a dot. The colored ribbon indicates secondary structure as seen in Figs. 2 and 4. C,D) For each residue, the proportion Gain-of-Trafficking (GoT) and Loss-of-Trafficking (LoT) missense variants is displayed. F) The number of unique variants observed in the -KCNQ1 trafficking map per position (max = 21).

## Fig. S8: Development of functional assay and distribution of functional scores

A) Variant abundances in the KCNE1 functional assay at various timepoints (in the subassembly/original library, at day 0, day 8, and day 20 of the growth experiment). Variant frequencies are expressed as normalized reads per million. B) Proportion of sequencing reads per variant category across different sequencing experiments: original subassembly, trafficking assay (conducted in the presence or absence of KCNQ1-WT; sequenced at day 8), functional assay (conducted in KCNQ1-S140G; cell fitness measured at day 8 and day 20). C) Quality control prior to functional score calculation across three replicates. The coefficient of variation of the synonymous distribution (top), and the mean and median (middle, black and red respectively) synonymous variant scores as a function of variant frequency at day 0, across each replicate experiment. The number of unique missense variants that passed each variant frequency cutoff (bottom). A cutoff of 31.6 (10^1.5^, dotted line) was determined to minimize the coefficient of variation and maximize the number of unique missense variants for which scores were calculated. Variants with less than 31.6 reads per million at day 0 in any replicate were excluded. D) inter-replicate Spearman correlation of functional scores based on variant category. ***: p-value < 0.001; **: p-value < 0.01; *: p-value < 0.05. Early and late nonsense variants are defined as variants at residues 1-55 and 56-129 respectively.


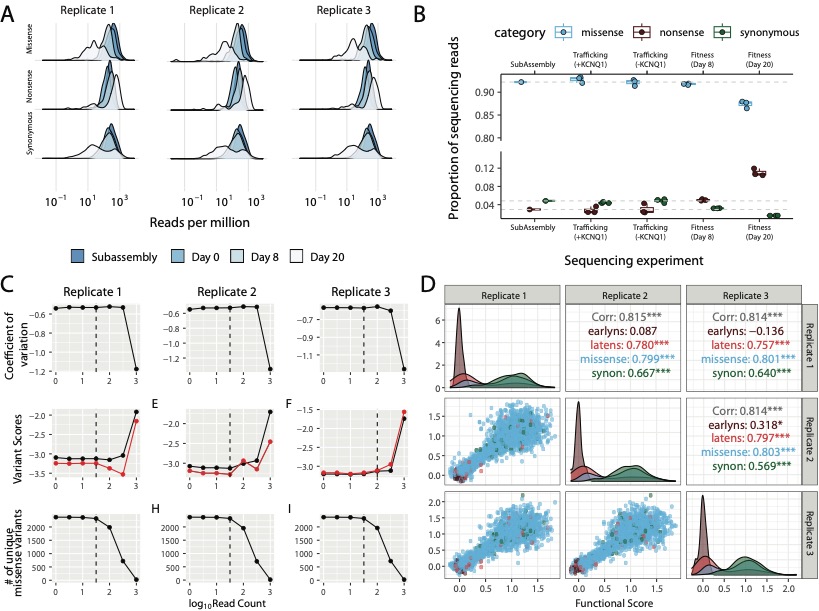


## Fig. S9: Relationship of functional and trafficking variant scores and validation of gain-of-function variants

A) Distribution of nonsense variant functional and trafficking scores show three categories of nonsense variants: those with trafficking and functional defects (brown, residue 1-55), those with WT-like trafficking but gating defects (red, residue 56-104) and those with WT-like trafficking and function (pink, residue 105+). B-C) Relationship between trafficking scores and functional scores. The loess best fit line and 95% confidence interval are plotted in blue. For trafficking scores between 0-1, there is a correlation between trafficking scores and functional scores. The vertical red lines indicate the cutoffs for complete loss, partial loss or increase of trafficking (0.20 or 97.5^th^ percentile of the early nonsense distribution, 0.83 or 2.5^th^ percentile of the synonymous distribution, and 1.18 or 97.5^th^ percentile of synonymous distribution). Panel C shows complete loss-of-function variants. However, some partial loss-of-trafficking variants (scores between 0.20 and 0.83) have normal functional scores. Some gain-of-trafficking variants also have loss-of-function scores. D) Bar chart showing proportion of different functional score categories for each trafficking score category. Cutoffs for each category are defined in *Supplemental Methods*. E-F) Variants at glycosylation sites alter trafficking in the absence of KCNQ1 but do not affect functional scores.


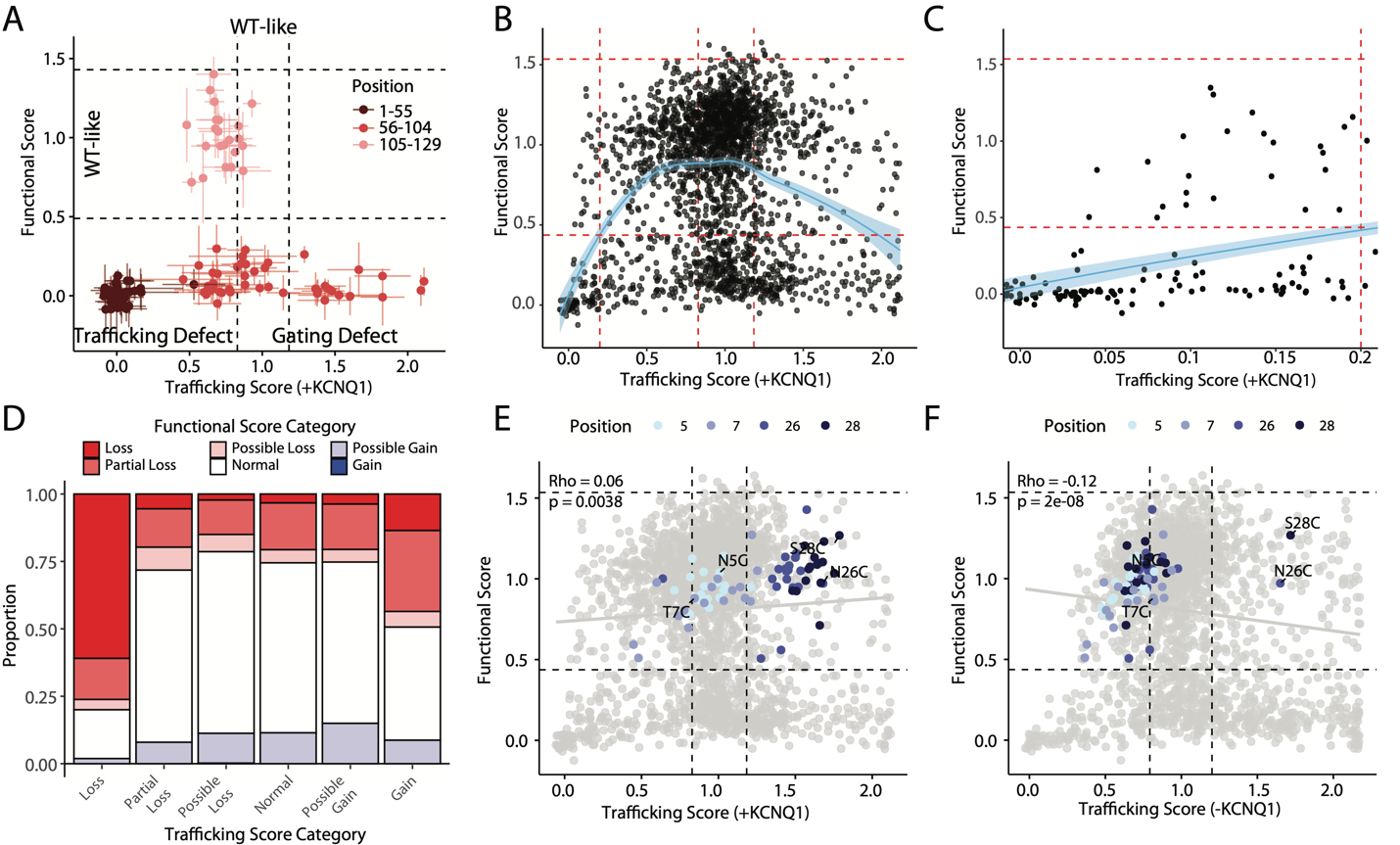


## Fig. S10: Patch clamp data and splicing evaluation for individual variants

A) Peak potassium currents of WT KCNE1-HA and R32T, a variant classified as partial loss-of-trafficking but normal function in our assays (n=4 cells each). B) Peak and tail potassium currents of WT KCNE1-HA and Y107R and C106L, variants classified as possible gain-of-function (n=7 cells each). C) SpliceAI delta scores as a function of MAVE functional assay scores. 8 KCNE1 variants were moderately predicted to disrupt splicing (score between 0.2 and 0.4), and 1/8 had a low functional score. See Supplemental File 3 for SpliceAI scores. D) KCNE1-HA and KCNQ1-S140G retain a beta-adrenergic response in patch clamp measurements of I_Ks_ current in HEK293 cells. Left top: example trace of WT KCNQ1 and WT KCNE1-HA. Left bottom: Example trace of KCNQ1-S140G and WT KCNE1-HA. Right: Acute response of KCNQ1-S140G and WT KCNE1-HA to forskolin and isobutylmethylxanthin (IBMX) which increase cAMP and mimic beta-adrenergic response. Traces in the panel on the right are consecutive measurements in the same cell, taken every 15 seconds. Addition of forskolin and IBMX resulted in increased current over time. Insets: voltage protocols used in panel D.


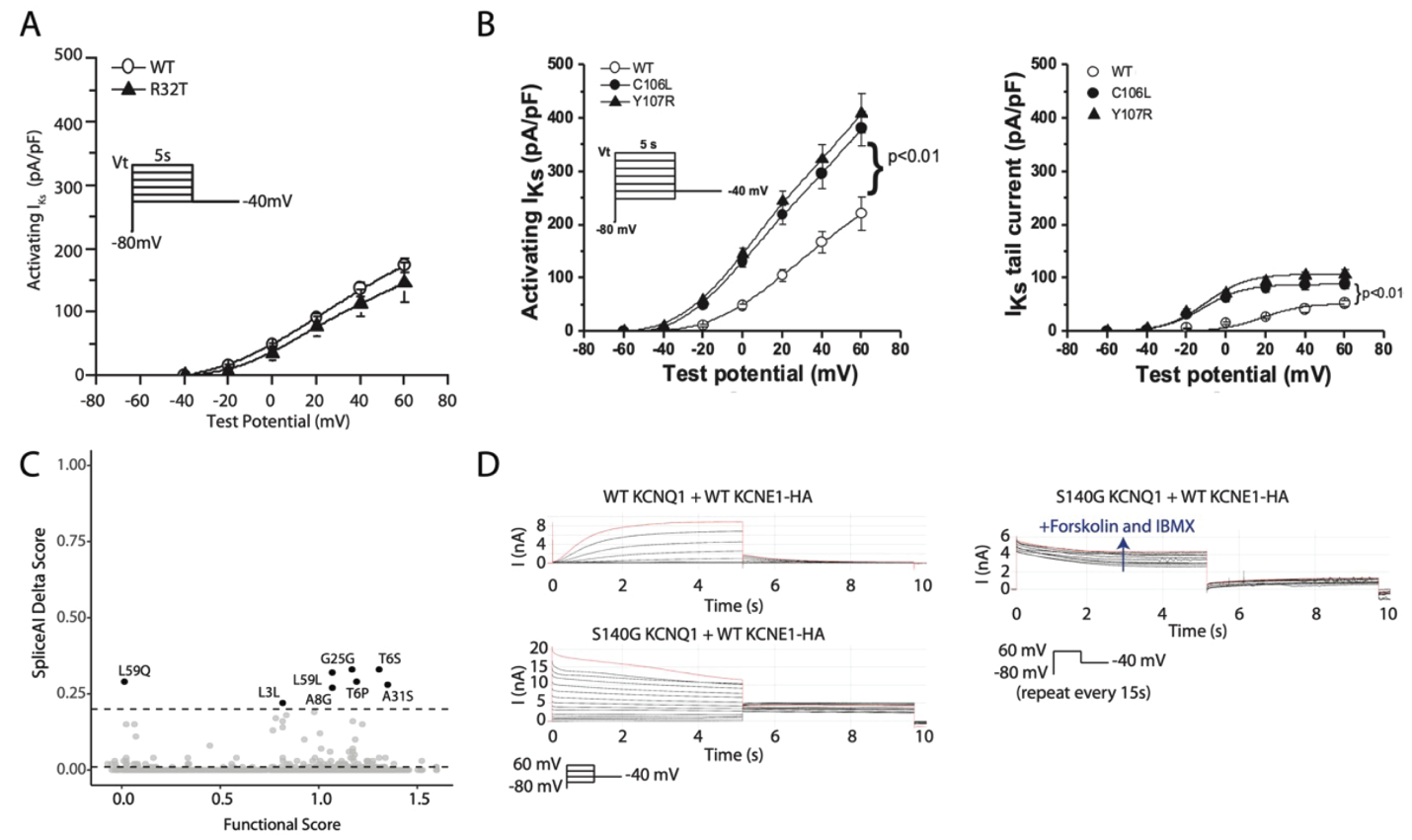


## Fig. S11: Comparison of structural models of full-length KCNE1 from various structure prediction methods and NMR

The image of each model generated by ESMFold,^8^ trRosetta,^9^ and NMR (PDB ID: 2K21)^14^ was rendered after alignment with the model predicted using AlphaFold-Multimer. After considering these structures in light of the KCNQ1:KCNE3 homology model, the AlphaFold-Multimer structure was selected as the primary structure for structural analyses in this study.


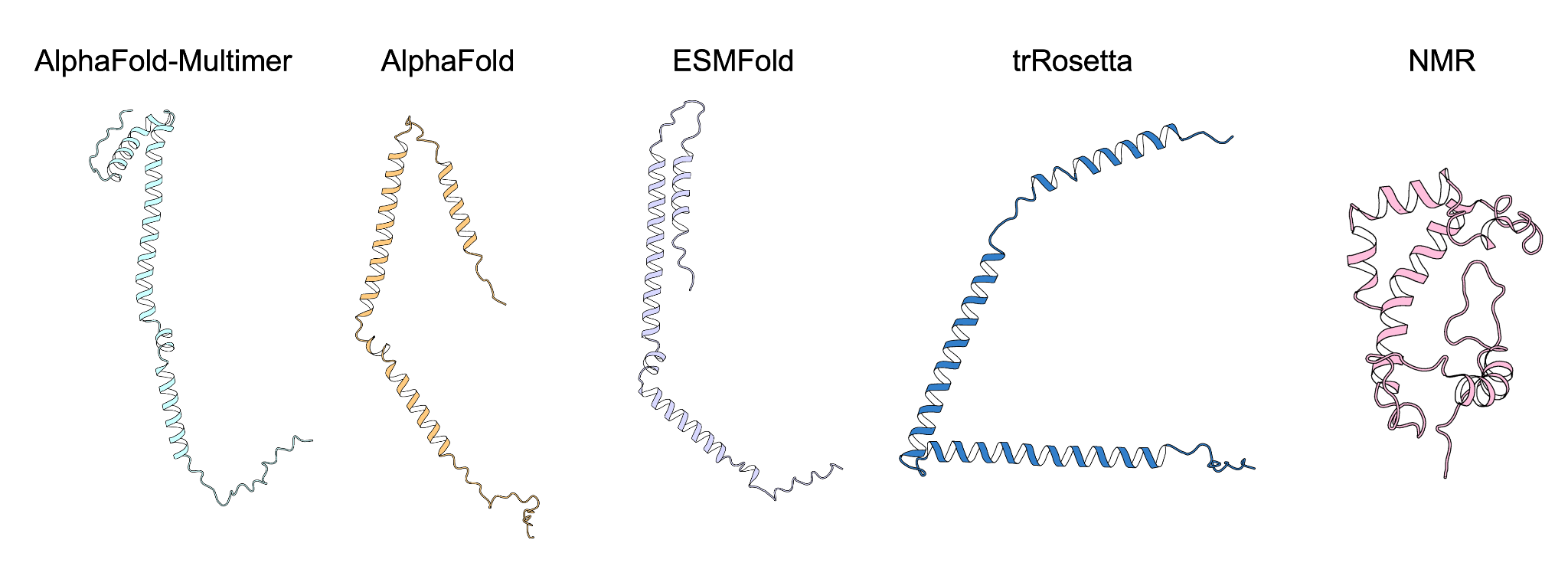


## Fig. S12: Correlation of functional and trafficking scores with electrophysiological parameters and genomic variant categories

A-C) In addition to their strong correlation with peak current (Fig. 6A), functional scores also correlate with other electrophysiological properties of the I_Ks_ channel (A: V_½_ activation deviated from WT, B: deactivation time constant, as a percent of WT, C: current at -20mV). D-F) Trafficking scores are less correlated with electrophysiological properties (C: peak current, D: V_½_ activation, and E: deactivation time constant) than functional scores. G) Variants achievable by a single SNV (blue) are less likely to be trafficking-deficient than all other variants (black; p = 7.4x10^-5^, Wilcoxon test). H-I) Variants achieved by genomic transitions (black) are less likely to be functionally deleterious (H) than variants achieved by genomic transversions (gray; p = 3.4x10^-5^, Wilcoxon test). The effect does not exist in trafficking variants (H; p = 0.973, Wilcoxon test).

**
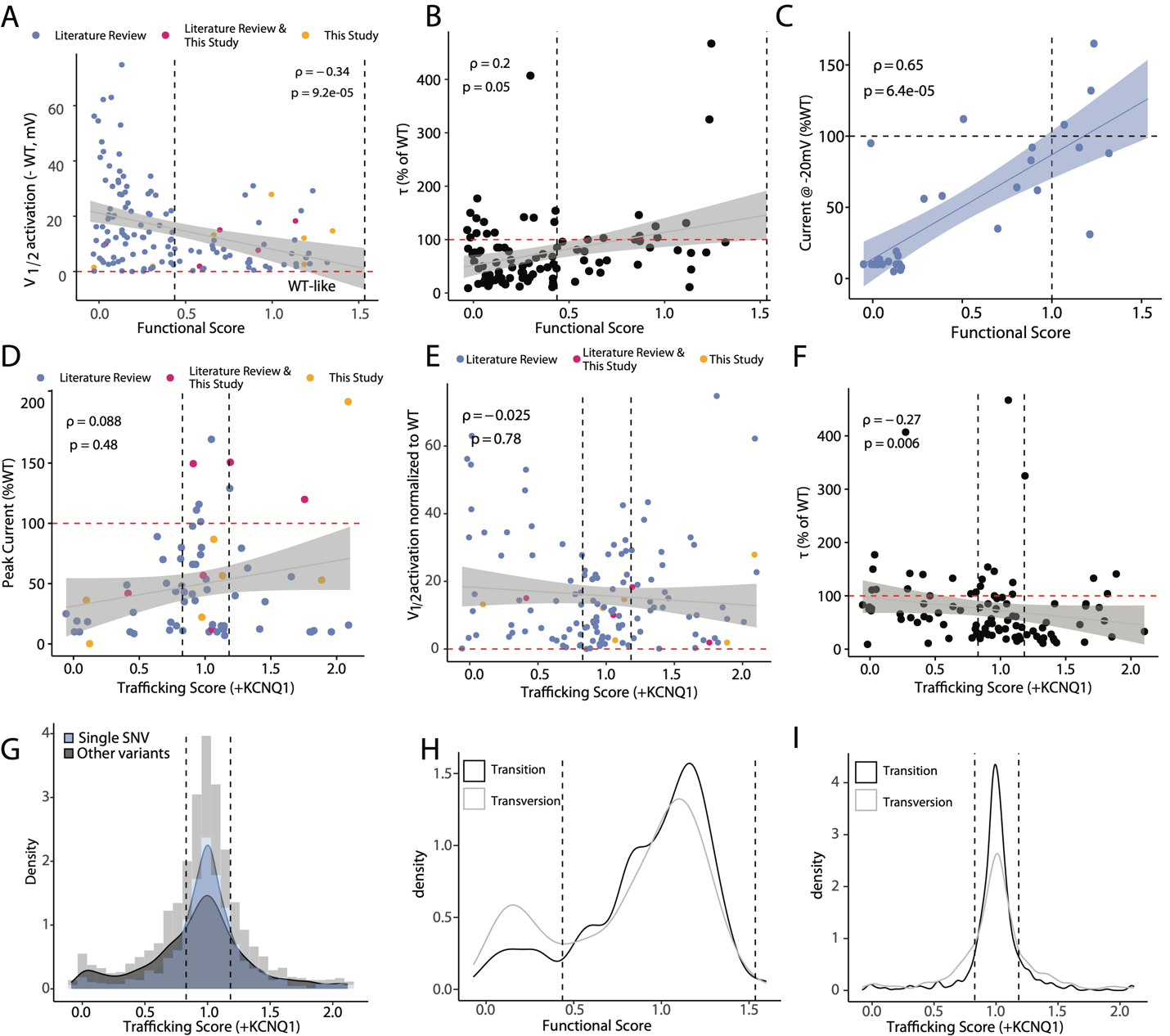
**

## Fig. S13: Distribution of functional scores by gnomAD category and MAVE data prediction performance

A) Variants more common in gnomAD are more likely to have WT-like functional scores. B) Receiver operator characteristic curves evaluating prediction of variant pathogenicity based on computational metrics and MAVE data. MAVE functional scores perform equally as well as previously developed computational metrics of protein function and evolution.

**
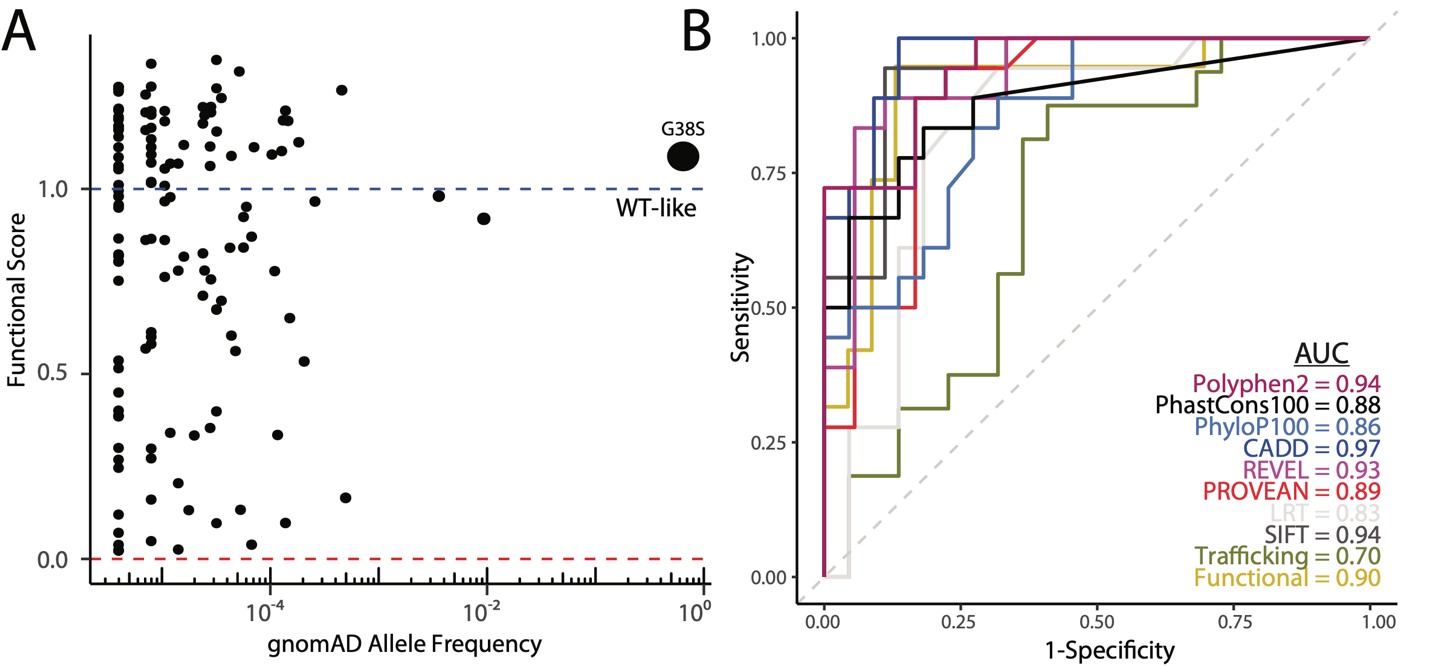
**

## Fig. S14: Spearman correlation of functional scores to previously developed computational predictors of protein function and evolution

Correlation coefficients and p-values are given. Blue dotted lines represent the trend lines.

**
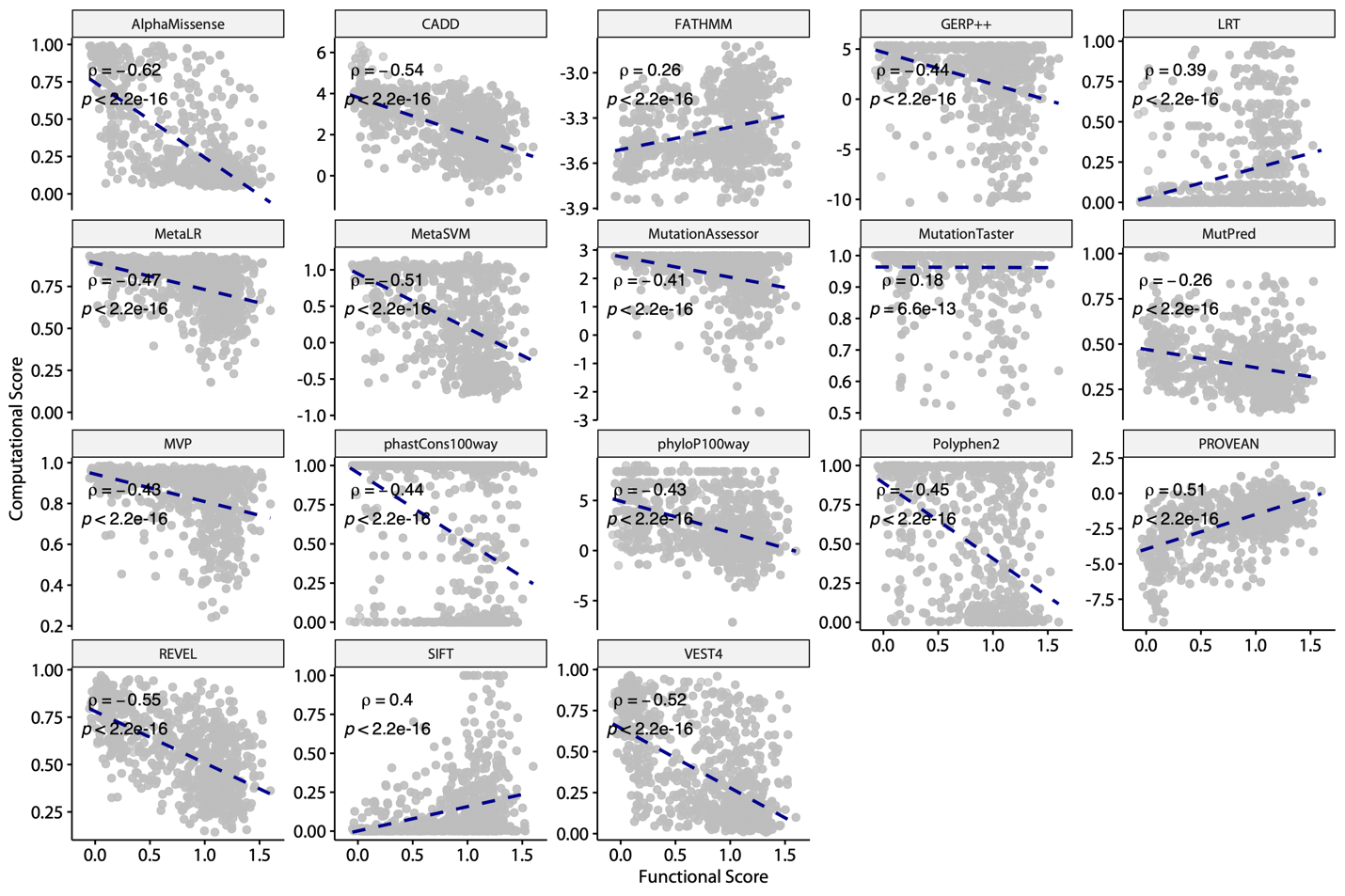
**

## Fig. S15: Correlation of trafficking scores to population and clinical case/control data

A) Variants more common in gnomAD are more likely to have WT-like trafficking scores. B) Distribution of trafficking scores of variants present in and absent from gnomAD (black and grey, respectively). Variants absent from gnomAD are slightly more likely to have low trafficking scores (p = 4.2x10^-3^, Wilcoxon test) C) Trafficking score distribution of variants annotated in ClinVar by category. 3/4 P/LP variants have low scores, and 5/7 B/LB variants have WT-like trafficking scores. D) Trafficking score distributions of presumed benign and presumed pathogenic variants.


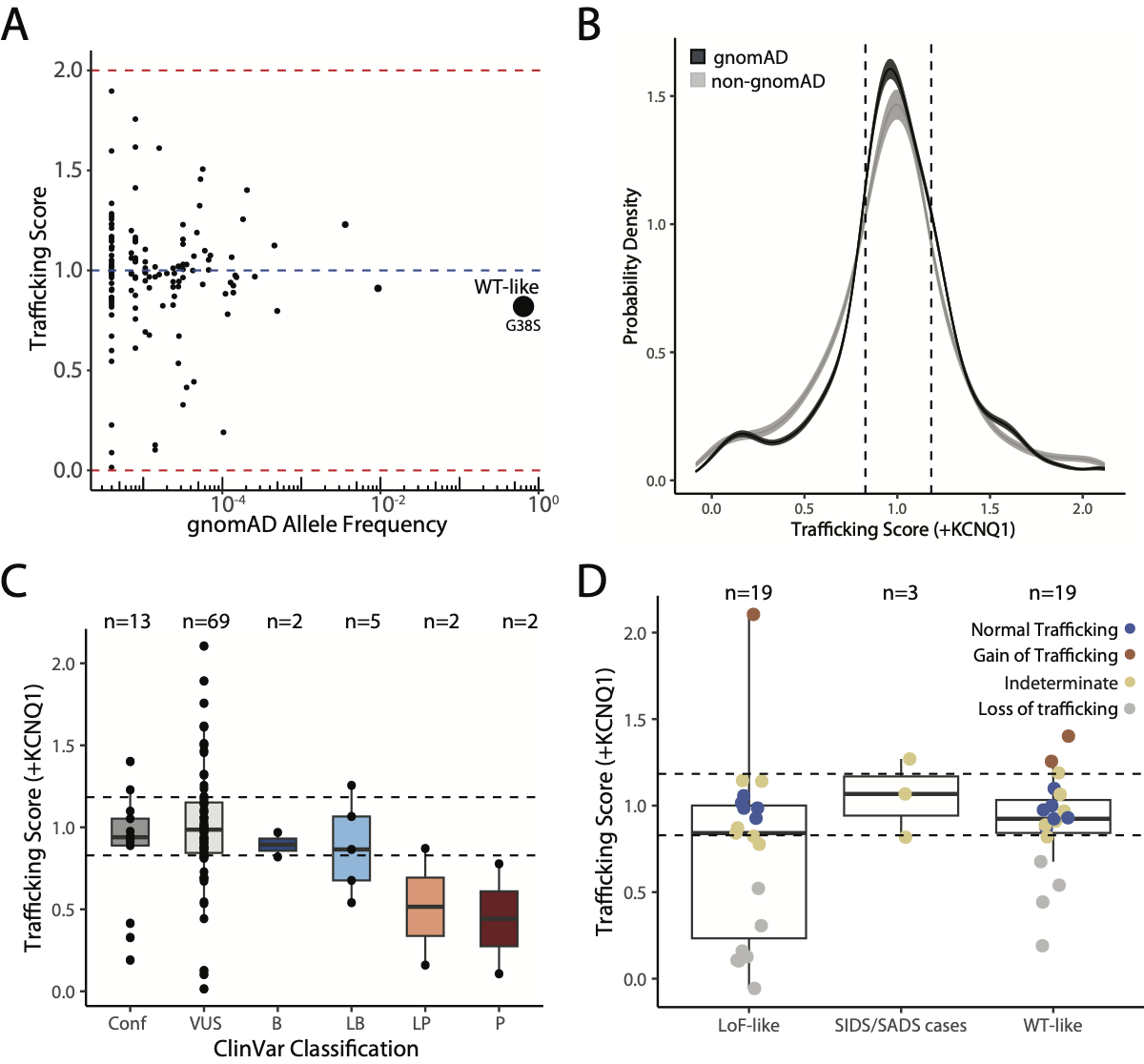


## Fig. S16: Spearman correlation of trafficking scores to previously developed computational predictors of protein function and evolution

Since gain-of-trafficking variants have been associated with a deleterious phenotype, i.e., atrial fibrillation, we checked the correlation of computational metrics to deviation of trafficking score from WT. Spearman correlations and p-values are given. Blue dotted lines represent the trend lines.

**
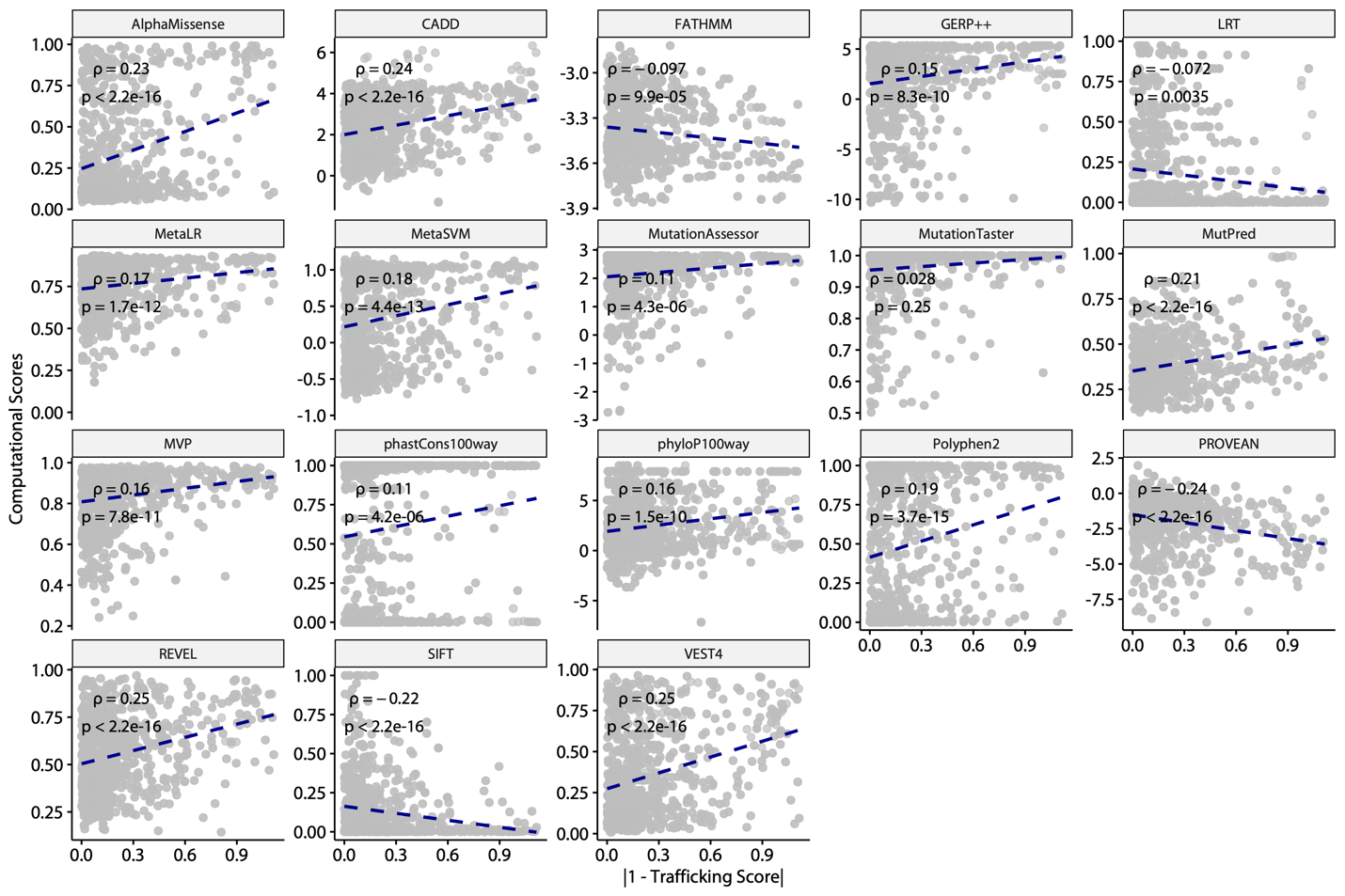
**

## Fig. S17: Convergence of the docking calculation of KCNE1 to KCNQ1 and calmodulin complex

The red dot represents the docked complex with the lowest binding energy (in Rosetta Energy Unit or REU; more negative values mean stronger binding). This complex was selected for structural analysis. Interface root-mean-square distance (r.m.s.d.) measures the average deviation of interface residues in all other docked models from the interface residues in the lowest-energy model. Interface residues are defined as residues in KCNE1 that are within 8Å from any KCNQ1 or calmodulin residues. The plot shows a typical “energy funnel” where binding energy becomes more negative as r.m.s.d. becomes smaller, suggesting the convergence of docking.


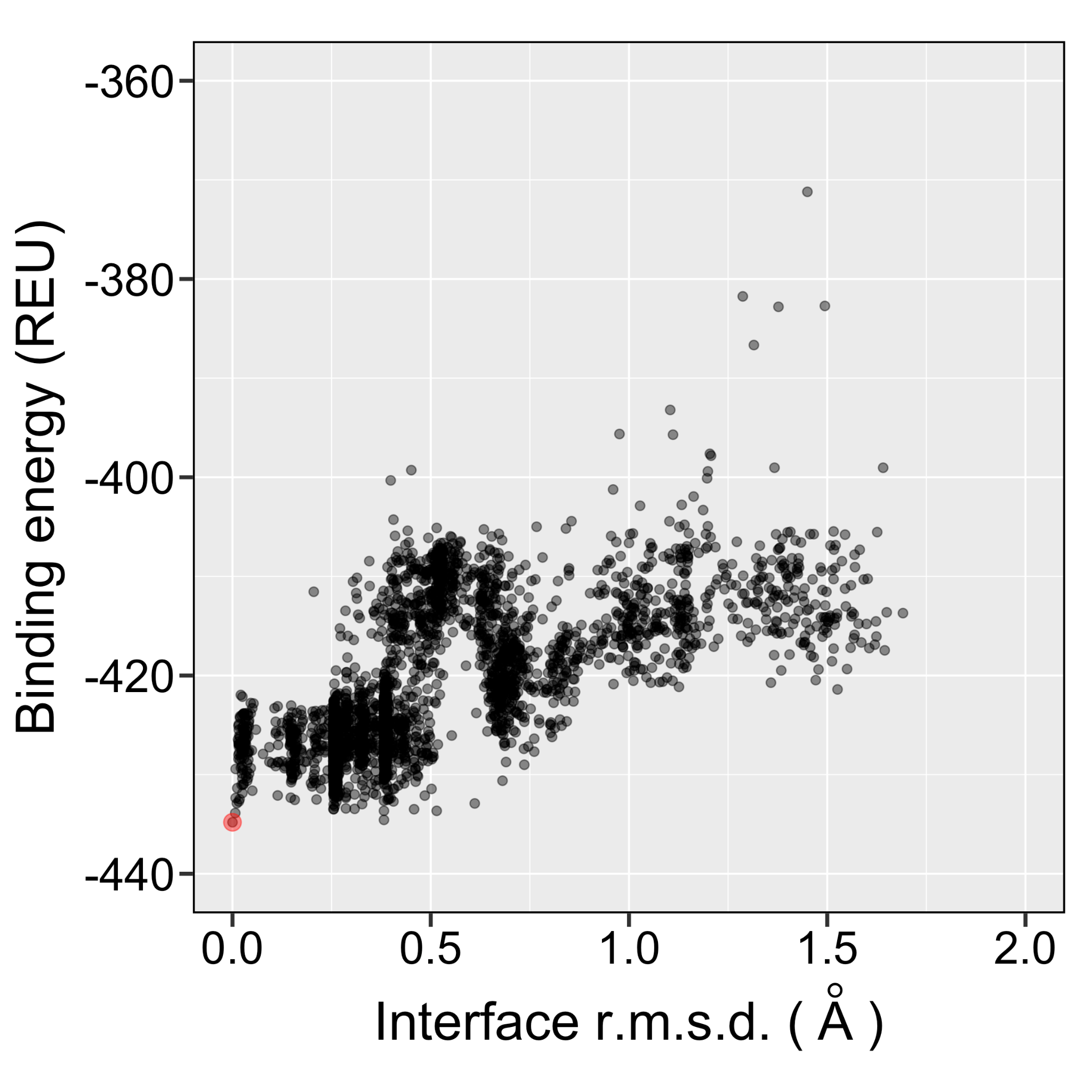


# Supplemental Tables

## Table S1: Primers used in this study

| **Primer** | **Sequence** | **Description** |
| --- | --- | --- |
| ag158 | gttcctgtagcggcggcgactctagatca | Quikchange mutate out NotI site from pIRES2-dsRed2 |
| ag201 | tgtacaagtaaagcggcggcgactctagatcataa | Quikchange mutate out NotI site from pIRES2:EGFP |
| ag289 | attacgtacgcttaagNNNNNNNNNNNNNNNNNNgacgtcctctagagcca | Barcode forward |
| ag290 | tggctctagaggacgtc | Barcode reverse |
| ag409 | attagcggccgcatgatcctgtctaacaccacagcg | Forward primer to amplify KCNE1-HA and add NotI site to subclone it to pIRES2-dsRed2 |
| ag410 | attagcggccgctcatggggaaggcttcgtctc | Reverse primer to amplify KCNE1-HA and add NotI site to subclone it to pIRES2-dsRed2 |
| ag422 | TACCAGATTATGCGCAGGGTGGCAACATGTCGG | KCNE1 HA tag 22-23 forward cloning primer (inverse PCR) |
| ag423 | CATCGTAGGGGTACTGAACTGTCTCCTGCCACAGC | KCNE1 HA tag 22-23 reverse cloning primer (inverse PCR) |
| ag424 | TACCAGATTATGCGCCCCGCAGCGGTGAC | KCNE1 HA tag 34-35 forward cloning primer (inverse PCR) |
| ag425 | CATCGTAGGGGTAGGACCTGCGGGCCAGG | KCNE1 HA tag 34-35 reverse cloning primer (inverse PCR) |
| ag594 | gaggtcgacgatgtaggtcacggcattccggaGCGGCCGCGACGTCatgatcctgtctaacaccacagcg | Forward primer for amplifying KCNE1 to Gibson assemble it into zone plasmid |
| ag595 | aagctgcaataaacaagttaacaacaacaattgGCGATCGGCGGCCGCCTTAAGtcatggggaaggcttcgtct | Reverse primer for amplifying KCNE1 to Gibson assemble it into zone plasmid |
| ag596 | CTCTACGTCCTCATGGTACATGGATTCTTCGGCTTCTTCA | Quikchange KCNE1 L51H |
| ag597 | CACACCTTCCTGAGACGAAGACTTCCCCATGA | Quikchange KCNE1 P127T |
| ag612 | attaggccatatggccatggccgcggcctc | Forward primer to amplify KCNQ1 with SfiI to clone into sleeping beauty plasmid |
| ag613 | attaggccatatggcctcaggacccctcatcggg | Reverse primer to amplify KCNQ1 with SfiI to clone into sleeping beauty plasmid |
| ag1591 | AATGATACGGCGACCACCGAGATCTACACATGAGATCATACACTCTTTCCCTACACGACGCTCTTCCGATCTTCTTCGCCCTTAGACACCAT | NovaSeq i5 for amplifying barcode after cell integration |
| ag1592 | AATGATACGGCGACCACCGAGATCTACACGCAGAGCTGCACACTCTTTCCCTACACGACGCTCTTCCGATCTTCTTCGCCCTTAGACACCAT | NovaSeq i5 for amplifying barcode after cell integration |
| ag1593 | AATGATACGGCGACCACCGAGATCTACACTGTCGCTGGTACACTCTTTCCCTACACGACGCTCTTCCGATCTTCTTCGCCCTTAGACACCAT | NovaSeq i5 for amplifying barcode after cell integration |
| ag1594 | AATGATACGGCGACCACCGAGATCTACACCACTATCAACACACTCTTTCCCTACACGACGCTCTTCCGATCTTCTTCGCCCTTAGACACCAT | NovaSeq i5 for amplifying barcode after cell integration |
| ag1595 | AATGATACGGCGACCACCGAGATCTACACCTCTGCAGCGACACTCTTTCCCTACACGACGCTCTTCCGATCTTCTTCGCCCTTAGACACCAT | NovaSeq i5 for amplifying barcode after cell integration |
| ag1596 | AATGATACGGCGACCACCGAGATCTACACTCTCATGATAACACTCTTTCCCTACACGACGCTCTTCCGATCTTCTTCGCCCTTAGACACCAT | NovaSeq i5 for amplifying barcode after cell integration |
| ag1597 | AATGATACGGCGACCACCGAGATCTACACTATCTTGTAGACACTCTTTCCCTACACGACGCTCTTCCGATCTTCTTCGCCCTTAGACACCAT | NovaSeq i5 for amplifying barcode after cell integration |
| ag1598 | AATGATACGGCGACCACCGAGATCTACACCGCTCCACGAACACTCTTTCCCTACACGACGCTCTTCCGATCTTCTTCGCCCTTAGACACCAT | NovaSeq i5 for amplifying barcode after cell integration |
| ag1599 | AATGATACGGCGACCACCGAGATCTACACATTGCCGAGTACACTCTTTCCCTACACGACGCTCTTCCGATCTTCTTCGCCCTTAGACACCAT | NovaSeq i5 for amplifying barcode after cell integration |
| ag1600 | AATGATACGGCGACCACCGAGATCTACACGCCATTAGACACACTCTTTCCCTACACGACGCTCTTCCGATCTTCTTCGCCCTTAGACACCAT | NovaSeq i5 for amplifying barcode after cell integration |
| ag1601 | AATGATACGGCGACCACCGAGATCTACACAGCACATCCTACACTCTTTCCCTACACGACGCTCTTCCGATCTTCTTCGCCCTTAGACACCAT | NovaSeq i5 for amplifying barcode after cell integration |
| ag1602 | AATGATACGGCGACCACCGAGATCTACACGATGTGCTTCACACTCTTTCCCTACACGACGCTCTTCCGATCTTCTTCGCCCTTAGACACCAT | NovaSeq i5 for amplifying barcode after cell integration |
| ag1603 | CAAGCAGAAGACGGCATACGAGATGCCGACAAGAGTGACTGGAGTTCAGACGTGTGCTCTTCCGATCTcggcAattccggaCGTACG | NovaSeq i7 for amplifying barcode after cell integration |
| ag1604 | CAAGCAGAAGACGGCATACGAGATATTAGTGGAGGTGACTGGAGTTCAGACGTGTGCTCTTCCGATCTcggcAattccggaCGTACG | NovaSeq i7 for amplifying barcode after cell integration |
| ag1605 | CAAGCAGAAGACGGCATACGAGATCTGGCTTGCCGTGACTGGAGTTCAGACGTGTGCTCTTCCGATCTcggcAattccggaCGTACG | NovaSeq i7 for amplifying barcode after cell integration |
| ag1606 | CAAGCAGAAGACGGCATACGAGATTCAATCCATTGTGACTGGAGTTCAGACGTGTGCTCTTCCGATCTcggcAattccggaCGTACG | NovaSeq i7 for amplifying barcode after cell integration |
| ag1609 | CAAGCAGAAGACGGCATACGAGATCTGTTGGTCCGTGACTGGAGTTCAGACGTGTGCTCTTCCGATCTcggcAattccggaCGTACG | NovaSeq i7 for amplifying barcode after cell integration |
| ag1610 | CAAGCAGAAGACGGCATACGAGATTCACCAACTTGTGACTGGAGTTCAGACGTGTGCTCTTCCGATCTcggcAattccggaCGTACG | NovaSeq i7 for amplifying barcode after cell integration |
| ag1611 | CAAGCAGAAGACGGCATACGAGATGGTGCTATATGTGACTGGAGTTCAGACGTGTGCTCTTCCGATCTcggcAattccggaCGTACG | NovaSeq i7 for amplifying barcode after cell integration |
| ag1612 | CAAGCAGAAGACGGCATACGAGATAACATCGCGCGTGACTGGAGTTCAGACGTGTGCTCTTCCGATCTcggcAattccggaCGTACG | NovaSeq i7 for amplifying barcode after cell integration |
| ag1613 | CAAGCAGAAGACGGCATACGAGATGGTGGACGTGGTGACTGGAGTTCAGACGTGTGCTCTTCCGATCTcggcAattccggaCGTACG | NovaSeq i7 for amplifying barcode after cell integration |
| ag1614 | CAAGCAGAAGACGGCATACGAGATAACAAGTACAGTGACTGGAGTTCAGACGTGTGCTCTTCCGATCTcggcAattccggaCGTACG | NovaSeq i7 for amplifying barcode after cell integration |
| ag1615 | CAAGCAGAAGACGGCATACGAGATACCGTTACAAGTGACTGGAGTTCAGACGTGTGCTCTTCCGATCTcggcAattccggaCGTACG | NovaSeq i7 for amplifying barcode after cell integration |
| ag1616 | CAAGCAGAAGACGGCATACGAGATGTTACCGTGGGTGACTGGAGTTCAGACGTGTGCTCTTCCGATCTcggcAattccggaCGTACG | NovaSeq i7 for amplifying barcode after cell integration |
| ag1818 | CCTGGAGAGCTACAGGTCGCTCTATGTCGTTGAAAACCAT | Quikchange KCNE1 C106L |
| ag1819 | GGAGAGCTACAGGTCGTGCCGTGTCGTTGAAAACCATCTG | Quikchange KCNE1 Y107R |
| ag2001 | ATGTCGGGCCTGGCCACCAGGTCCTACCC | Quikchange KCNE1 R32T |
| am5 | tctgcctcatcttcggcgtgctgtccacc | Quikchange KCNQ1 S140G Forward |
| am6 | ggtggacagcacgccgaagatgaggcaga | Quikchange KCNQ1 S140G Reverse |
| am10 | aatgatacggcgaccaccgagatctacacgccttgcgtctttccctacacgacgctcttccgatcttcttcgcccttagacaccat | NovaSeq i5 for amplifying barcode after cell integration |
| am11 | aatgatacggcgaccaccgagatctacactcctggtttctttccctacacgacgctcttccgatcttcttcgcccttagacaccat | NovaSeq i5 for amplifying barcode after cell integration |
| am12 | aatgatacggcgaccaccgagatctacaccttcaacctctttccctacacgacgctcttccgatcttcttcgcccttagacaccat | NovaSeq i5 for amplifying barcode after cell integration |
| am13 | aatgatacggcgaccaccgagatctacactcgctatgtctttccctacacgacgctcttccgatcttcttcgcccttagacaccat | NovaSeq i5 for amplifying barcode after cell integration |
| am14 | aatgatacggcgaccaccgagatctacacctatcgcatctttccctacacgacgctcttccgatcttcttcgcccttagacaccat | NovaSeq i5 for amplifying barcode after cell integration |
| am15 | aatgatacggcgaccaccgagatctacaccattagtgtctttccctacacgacgctcttccgatcttcttcgcccttagacaccat | NovaSeq i5 for amplifying barcode after cell integration |
| am20 | caagcagaagacggcatacgagataacgttaggtgactggagttcagacgtgtgctcttccgatctcggcaattccggacgtacg | NovaSeq i7 for amplifying barcode after cell integration |
| am21 | caagcagaagacggcatacgagatggtctattgtgactggagttcagacgtgtgctcttccgatctcggcaattccggacgtacg | NovaSeq i7 for amplifying barcode after cell integration |
| am22 | caagcagaagacggcatacgagataactcgccgtgactggagttcagacgtgtgctcttccgatctcggcaattccggacgtacg | NovaSeq i7 for amplifying barcode after cell integration |
| am23 | caagcagaagacggcatacgagatggagtgccgtgactggagttcagacgtgtgctcttccgatctcggcaattccggacgtacg | NovaSeq i7 for amplifying barcode after cell integration |
| am24 | caagcagaagacggcatacgagataagacattgtgactggagttcagacgtgtgctcttccgatctcggcaattccggacgtacg | NovaSeq i7 for amplifying barcode after cell integration |
| am25 | caagcagaagacggcatacgagattcgagccagtgactggagttcagacgtgtgctcttccgatctcggcaattccggacgtacg | NovaSeq i7 for amplifying barcode after cell integration |
| am30 | caagcagaagacggcatacgagatctagattggtgactggagttcagacgtgtgctcttccgatctcggcaattccggacgtacg | NovaSeq i7 for amplifying barcode after cell integration |
| am110 | caagcagaagacggcatacgagatggtaccgaccgtgactggagttcagacgtgtgctcttccgatct cggcaattccggacgtacg | NovaSeq sequencing primer for KCNE1 subassembly i7 |
| am110c | caagcagaagacggcatacgagattatcatgagagtgactggagttcagacgtgtgctcttccgatctcggcaattccggacgtacg | NovaSeq sequencing primer for KCNE1 subassembly i7 |
| am111 | aatgatacggcgaccaccgagatctacacgtggtatctgacactctttccctacacgacgctcttccgatctccggtgcggccgccttaag | NovaSeq sequencing primer for KCNE1 subassembly i5 |
| am112 | aatgatacggcgaccaccgagatctacacgtggtatctgacactctttccctacacgacgctcttccgatctttcgagtgctccagcttct | NovaSeq sequencing primer for KCNE1 subassembly i5 |
| am113 | aatgatacggcgaccaccgagatctacacgtggtatctgacactctttccctacacgacgctcttccgatcttggagcggatgtagctcag | NovaSeq sequencing primer for KCNE1 subassembly i5 |
| am114 | aatgatacggcgaccaccgagatctacacgtggtatctgacactctttccctacacgacgctcttccgatctgtctcaggaaggtgtgtgt | NovaSeq sequencing primer for KCNE1 subassembly i5 |
| am115 | aatgatacggcgaccaccgagatctacacgtggtatctgacactctttccctacacgacgctcttccgatcttgggttgttctatggccag | NovaSeq sequencing primer for KCNE1 subassembly i5 |
| am116 | aatgatacggcgaccaccgagatctacacgtggtatctgacactctttccctacacgacgctcttccgatctaatctggtacatcgtaggggta | NovaSeq sequencing primer for KCNE1 subassembly i5 |
| am120 | caagcagaagacggcatacgagataggccgtggtgactggagttcagacgtgtgctcttccgatctcggcaattccggacgtacg | NovaSeq i7 for amplifying barcode after cell integration |
| am125 | aatgatacggcgaccaccgagatctacacacaacgcttctttccctacacgacgctcttccgatcttcttcgcccttagacaccat | NovaSeq i5 for amplifying barcode after cell integration |
| am126 | aatgatacggcgaccaccgagatctacacattccatatctttccctacacgacgctcttccgatcttcttcgcccttagacaccat | NovaSeq i5 for amplifying barcode after cell integration |
| am178 | gcgacgtcatgatcccgtctaacaccacagc | Quikchange KCNE1 L3P |
| am179 | ccaagctgtggcaggatacagttcagcaggg | Quikchange KCNE1 E19D |
| am180 | ccgcagcggtggcggcaagctgg | Quikchange KCNE1 D39G |
| am181 | ggtactgggattcttcggcttctataccctgggcatc | Quikchange KCNE1 F57Y Forward |
| am182 | cgtctacatcgagtccaatgcctggcaagagaa | Quikchange KCNE1 D85N |
| am183 | cccgggtcctggtgagctacaggtc | Quikchange KCNE1 E101V |
| am184 | cacaccttcctgagatgaagccttccccatg | Quikchange KCNE1 T125M |
| am185 | tcctcatggtactgggatagttcggcttcttcaccc | Quikchange KCNE1 F53X Forward |
| am186 | ctcatggtactgggattcttctagttcttcaccctgggcatcatg | Quikchange KCNE1 G55X Forward |
| am189 | gcttcttcaccctgggctagatgctgagctacatccg | Quikchange KCNE1 I61X Forward |
| am191 | gggtgaagaagccgaactatcccagtaccatgagga | Quikchange KCNE1 F53X Reverse |
| am194 | agttcagcagggtgtcaacatgtcgggcc | Quikchange KCNE1 G25V Forward |
| am195 | ggcttcttcaccctggacatcatgctgagctac | Quikchange KCNE1 G60D Forward |
| am196 | ggcccgacatgttgacaccctgctgaact | Quikchange KCNE1 G25V Reverse |
| am197 | gtagctcagcatgatgtccagggtgaagaagcc | Quikchange KCNE1 G60D Reverse |
| am198 | catgatgcccagggtgaagaactagaagaatcccagtaccatgag | Quikchange KCNE1 G55X Reverse |
| am199 | gatgcccagggtatagaagccgaagaatcccagtacc | Quikchange KCNE1 F57Y Reverse |
| am206 | cggatgtagctcagcatctagcccagggtgaagaagc | Quikchange KCNE1 I61X Reverse |
| am208 | aatgatacggcgaccaccgagatctacacgtggtatctgacactctttccctacacgacgctcttccgatctttgccaggcatcggactcga | NovaSeq sequencing primer for KCNE1 subassembly i5 |
| am209 | aatgatacggcgaccaccgagatctacacgtggtatctgacactctttccctacacgacgctcttccgatctctcttgccaggcatcggact | NovaSeq sequencing primer for KCNE1 subassembly i5 |
| am210 | aatgatacggcgaccaccgagatctacacgtggtatctgacactctttccctacacgacgctcttccgatcttaggggtaggacctgcgggc | NovaSeq sequencing primer for KCNE1 subassembly i5 |
| bk196 | cttggcccgcgtccgccggtgag | Quikchange primer to mutate out NotI site in KCNQ1 |

## Table S2: Primers for Jain mutagenesis for *KCNE1* variant library generation

| **Position** | **Codon** | **Forward primer** | **Reverse primer** |
| --- | --- | --- | --- |
| 1 | ATG | NNNATCCTGTCTAACACCACAGCGGT | GACGTCGCGGCCGCTCC |
| 2 | ATC | NNNCTGTCTAACACCACAGCGGTGAC | CATGACGTCGCGGCCGCT |
| 3 | CTG | NNNTCTAACACCACAGCGGTGACGC | GATCATGACGTCGCGGCCG |
| 4 | TCT | NNNAACACCACAGCGGTGACGCC | CAGGATCATGACGTCGCGGC |
| 5 | AAC | NNNACCACAGCGGTGACGCCCTT | AGACAGGATCATGACGTCGCGG |
| 6 | ACC | NNNACAGCGGTGACGCCCTTTCTG | GTTAGACAGGATCATGACGTCGC |
| 7 | ACA | NNNGCGGTGACGCCCTTTCTGAC | GGTGTTAGACAGGATCATGACGTC |
| 8 | GCG | NNNGTGACGCCCTTTCTGACCAAGC | TGTGGTGTTAGACAGGATCATGACGT |
| 9 | GTG | NNNACGCCCTTTCTGACCAAGCTGTG | CGCTGTGGTGTTAGACAGGATCAT |
| 10 | ACG | NNNCCCTTTCTGACCAAGCTGTGGC | CACCGCTGTGGTGTTAGACAGG |
| 11 | CCC | NNNTTTCTGACCAAGCTGTGGCAGGA | CGTCACCGCTGTGGTGTTAGAC |
| 12 | TTT | NNNCTGACCAAGCTGTGGCAGGAG | GGGCGTCACCGCTGTGGT |
| 13 | CTG | NNNACCAAGCTGTGGCAGGAGACAG | AAAGGGCGTCACCGCTGTGG |
| 14 | ACC | NNNAAGCTGTGGCAGGAGACAGTTCA | CAGAAAGGGCGTCACCGCTG |
| 15 | AAG | NNNCTGTGGCAGGAGACAGTTCAGC | GGTCAGAAAGGGCGTCACCG |
| 16 | CTG | NNNTGGCAGGAGACAGTTCAGCAGG | CTTGGTCAGAAAGGGCGTCACC |
| 17 | TGG | NNNCAGGAGACAGTTCAGCAGGGTG | CAGCTTGGTCAGAAAGGGCGTC |
| 18 | CAG | NNNGAGACAGTTCAGCAGGGTGGC | CCACAGCTTGGTCAGAAAGGGC |
| 19 | GAG | NNNACAGTTCAGCAGGGTGGCAACAT | CTGCCACAGCTTGGTCAGAAAGG |
| 20 | ACA | NNNGTTCAGCAGGGTGGCAACATGTC | CTCCTGCCACAGCTTGGTCAG |
| 21 | GTT | NNNCAGCAGGGTGGCAACATGTCG | TGTCTCCTGCCACAGCTTGGTC |
| 22 | CAG | NNNCAGGGTGGCAACATGTCGGG | AACTGTCTCCTGCCACAGCTTGG |
| 23 | CAG | NNNGGTGGCAACATGTCGGGCCT | CTGAACTGTCTCCTGCCACAGC |
| 24 | GGT | NNNGGCAACATGTCGGGCCTGG | CTGCTGAACTGTCTCCTGCCAC |
| 25 | GGC | NNNAACATGTCGGGCCTGGCCC | ACCCTGCTGAACTGTCTCCTGC |
| 26 | AAC | NNNATGTCGGGCCTGGCCCG | GCCACCCTGCTGAACTGTCTC |
| 27 | ATG | NNNTCGGGCCTGGCCCGCAG | GTTGCCACCCTGCTGAACTGTC |
| 28 | TCG | NNNGGCCTGGCCCGCAGGTC | CATGTTGCCACCCTGCTGAACTG |
| 29 | GGC | NNNCTGGCCCGCAGGTCCTAC | CGACATGTTGCCACCCTGCTG |
| 30 | CTG | NNNGCCCGCAGGTCCTACCC | GCCCGACATGTTGCCACCCT |
| 31 | GCC | NNNCGCAGGTCCTACCCCTACGA | CAGGCCCGACATGTTGCCAC |
| 32 | CGC | NNNAGGTCCTACCCCTACGATGTACC | GGCCAGGCCCGACATGTTG |
| 33 | AGG | NNNTCCTACCCCTACGATGTACCAGATTA | GCGGGCCAGGCCCGAC |
| 34 | TCC | NNNTACCCCTACGATGTACCAGATTATGC | CCTGCGGGCCAGGCCC |
| 35 | CCC | NNNCGCAGCGGTGACGGCAAG | CGCATAATCTGGTACATCGTAGGG |
| 36 | CGC | NNNAGCGGTGACGGCAAGCTGGA | GGGCGCATAATCTGGTACATCGTA |
| 37 | AGC | NNNGGTGACGGCAAGCTGGAGG | GCGGGGCGCATAATCTGGTAC |
| 38 | GGT | NNNGACGGCAAGCTGGAGGCC | GCTGCGGGGCGCATAATCTG |
| 39 | GAC | NNNGGCAAGCTGGAGGCCCTCTA | ACCGCTGCGGGGCGCATAAT |
| 40 | GGC | NNNAAGCTGGAGGCCCTCTACGTC | GTCACCGCTGCGGGGCG |
| 41 | AAG | NNNCTGGAGGCCCTCTACGTCCT | GCCGTCACCGCTGCGGG |
| 42 | CTG | NNNGAGGCCCTCTACGTCCTCATG | CTTGCCGTCACCGCTGCG |
| 43 | GAG | NNNGCCCTCTACGTCCTCATGGTAC | CAGCTTGCCGTCACCGCTG |
| 44 | GCC | NNNCTCTACGTCCTCATGGTACTGGG | CTCCAGCTTGCCGTCACCG |
| 45 | CTC | NNNTACGTCCTCATGGTACTGGGATTCTT | GGCCTCCAGCTTGCCGTCA |
| 46 | TAC | NNNGTCCTCATGGTACTGGGATTCTTC | GAGGGCCTCCAGCTTGCC |
| 47 | GTC | NNNCTCATGGTACTGGGATTCTTCGG | GTAGAGGGCCTCCAGCTTGC |
| 48 | CTC | NNNATGGTACTGGGATTCTTCGGCTTCTT | GACGTAGAGGGCCTCCAGCT |
| 49 | ATG | NNNGTACTGGGATTCTTCGGCTTCTTCA | GAGGACGTAGAGGGCCTCCA |
| 50 | GTA | NNNCTGGGATTCTTCGGCTTCTTCAC | CATGAGGACGTAGAGGGCCTC |
| 51 | CTG | NNNGGATTCTTCGGCTTCTTCACCCT | TACCATGAGGACGTAGAGGGCC |
| 52 | GGA | NNNTTCTTCGGCTTCTTCACCCTGGG | CAGTACCATGAGGACGTAGAGGG |
| 53 | TTC | NNNTTCGGCTTCTTCACCCTGGGC | TCCCAGTACCATGAGGACGTAGA |
| 54 | TTC | NNNGGCTTCTTCACCCTGGGCATC | GAATCCCAGTACCATGAGGACGTA |
| 55 | GGC | NNNTTCTTCACCCTGGGCATCATGCT | GAAGAATCCCAGTACCATGAGGAC |
| 56 | TTC | NNNTTCACCCTGGGCATCATGCTGAG | GCCGAAGAATCCCAGTACCATGA |
| 57 | TTC | NNNACCCTGGGCATCATGCTGAGC | GAAGCCGAAGAATCCCAGTACCAT |
| 58 | ACC | NNNCTGGGCATCATGCTGAGCTACAT | GAAGAAGCCGAAGAATCCCAGTAC |
| 59 | CTG | NNNGGCATCATGCTGAGCTACATCCG | GGTGAAGAAGCCGAAGAATCCCA |
| 60 | GGC | NNNATCATGCTGAGCTACATCCGCTC | CAGGGTGAAGAAGCCGAAGAATC |
| 61 | ATC | NNNATGCTGAGCTACATCCGCTCCAA | GCCCAGGGTGAAGAAGCCGA |
| 62 | ATG | NNNCTGAGCTACATCCGCTCCAAGAA | GATGCCCAGGGTGAAGAAGCC |
| 63 | CTG | NNNAGCTACATCCGCTCCAAGAAGCT | CATGATGCCCAGGGTGAAGAAGC |
| 64 | AGC | NNNTACATCCGCTCCAAGAAGCTGGA | CAGCATGATGCCCAGGGTGAAG |
| 65 | TAC | NNNATCCGCTCCAAGAAGCTGGAGC | GCTCAGCATGATGCCCAGGG |
| 66 | ATC | NNNCGCTCCAAGAAGCTGGAGCAC | GTAGCTCAGCATGATGCCCAGG |
| 67 | CGC | NNNTCCAAGAAGCTGGAGCACTCGAA | GATGTAGCTCAGCATGATGCCCA |
| 68 | TCC | NNNAAGAAGCTGGAGCACTCGAACGA | GCGGATGTAGCTCAGCATGATGC |
| 69 | AAG | NNNAAGCTGGAGCACTCGAACGACC | GGAGCGGATGTAGCTCAGCATG |
| 70 | AAG | NNNCTGGAGCACTCGAACGACCC | CTTGGAGCGGATGTAGCTCAGC |
| 71 | CTG | NNNGAGCACTCGAACGACCCATTCAA | CTTCTTGGAGCGGATGTAGCTCA |
| 72 | GAG | NNNCACTCGAACGACCCATTCAACGT | CAGCTTCTTGGAGCGGATGTAGC |
| 73 | CAC | NNNTCGAACGACCCATTCAACGTCTACAT | CTCCAGCTTCTTGGAGCGGATG |
| 74 | TCG | NNNAACGACCCATTCAACGTCTACATCGA | GTGCTCCAGCTTCTTGGAGCG |
| 75 | AAC | NNNGACCCATTCAACGTCTACATCGAGT | CGAGTGCTCCAGCTTCTTGGAG |
| 76 | GAC | NNNCCATTCAACGTCTACATCGAGTCC | GTTCGAGTGCTCCAGCTTCTTGG |
| 77 | CCA | NNNTTCAACGTCTACATCGAGTCCGATG | GTCGTTCGAGTGCTCCAGCTTC |
| 78 | TTC | NNNAACGTCTACATCGAGTCCGATGC | TGGGTCGTTCGAGTGCTCCAG |
| 79 | AAC | NNNGTCTACATCGAGTCCGATGCCTG | GAATGGGTCGTTCGAGTGCTCC |
| 80 | GTC | NNNTACATCGAGTCCGATGCCTGGC | GTTGAATGGGTCGTTCGAGTGCT |
| 81 | TAC | NNNATCGAGTCCGATGCCTGGCAAG | GACGTTGAATGGGTCGTTCGAGT |
| 82 | ATC | NNNGAGTCCGATGCCTGGCAAGAG | GTAGACGTTGAATGGGTCGTTCGA |
| 83 | GAG | NNNTCCGATGCCTGGCAAGAGAAGG | GATGTAGACGTTGAATGGGTCGTTC |
| 84 | TCC | NNNGATGCCTGGCAAGAGAAGGACAA | CTCGATGTAGACGTTGAATGGGTC |
| 85 | GAT | NNNGCCTGGCAAGAGAAGGACAAGG | GGACTCGATGTAGACGTTGAATGG |
| 86 | GCC | NNNTGGCAAGAGAAGGACAAGGCCTAT | ATCGGACTCGATGTAGACGTTGAATG |
| 87 | TGG | NNNCAAGAGAAGGACAAGGCCTATGTC | GGCATCGGACTCGATGTAGACG |
| 88 | CAA | NNNGAGAAGGACAAGGCCTATGTCCA | CCAGGCATCGGACTCGATGTAG |
| 89 | GAG | NNNAAGGACAAGGCCTATGTCCAGGC | TTGCCAGGCATCGGACTCGATG |
| 90 | AAG | NNNGACAAGGCCTATGTCCAGGCC | CTCTTGCCAGGCATCGGACTC |
| 91 | GAC | NNNAAGGCCTATGTCCAGGCCCG | CTTCTCTTGCCAGGCATCGGAC |
| 92 | AAG | NNNGCCTATGTCCAGGCCCGG | GTCCTTCTCTTGCCAGGCATCG |
| 93 | GCC | NNNTATGTCCAGGCCCGGGTCCT | CTTGTCCTTCTCTTGCCAGGCAT |
| 94 | TAT | NNNGTCCAGGCCCGGGTCCT | GGCCTTGTCCTTCTCTTGCCAG |
| 95 | GTC | NNNCAGGCCCGGGTCCTGGA | ATAGGCCTTGTCCTTCTCTTGCCA |
| 96 | CAG | NNNGCCCGGGTCCTGGAGAG | GACATAGGCCTTGTCCTTCTCTTG |
| 97 | GCC | NNNCGGGTCCTGGAGAGCTACAG | CTGGACATAGGCCTTGTCCTTCT |
| 98 | CGG | NNNGTCCTGGAGAGCTACAGGTCG | GGCCTGGACATAGGCCTTGTC |
| 99 | GTC | NNNCTGGAGAGCTACAGGTCGTGC | CCGGGCCTGGACATAGGC |
| 100 | CTG | NNNGAGAGCTACAGGTCGTGCTATGT | GACCCGGGCCTGGACATAG |
| 101 | GAG | NNNAGCTACAGGTCGTGCTATGTCGTT | CAGGACCCGGGCCTGGA |
| 102 | AGC | NNNTACAGGTCGTGCTATGTCGTTGAAAAC | CTCCAGGACCCGGGCCT |
| 103 | TAC | NNNAGGTCGTGCTATGTCGTTGAAAACCA | GCTCTCCAGGACCCGGG |
| 104 | AGG | NNNTCGTGCTATGTCGTTGAAAACCATCTG | GTAGCTCTCCAGGACCCGG |
| 105 | TCG | NNNTGCTATGTCGTTGAAAACCATCTGGC | CCTGTAGCTCTCCAGGACCC |
| 106 | TGC | NNNTATGTCGTTGAAAACCATCTGGCCATAG | CGACCTGTAGCTCTCCAGGAC |
| 107 | TAT | NNNGTCGTTGAAAACCATCTGGCCATAGA | GCACGACCTGTAGCTCTCCAG |
| 108 | GTC | NNNGTTGAAAACCATCTGGCCATAGAACAAC | ATAGCACGACCTGTAGCTCTCCA |
| 109 | GTT | NNNGAAAACCATCTGGCCATAGAACAACC | GACATAGCACGACCTGTAGCTCT |
| 110 | GAA | NNNAACCATCTGGCCATAGAACAACCCAA | AACGACATAGCACGACCTGTAGCT |
| 111 | AAC | NNNCATCTGGCCATAGAACAACCCAACA | TTCAACGACATAGCACGACCTGTAG |
| 112 | CAT | NNNCTGGCCATAGAACAACCCAACACA | GTTTTCAACGACATAGCACGACCTGT |
| 113 | CTG | NNNGCCATAGAACAACCCAACACACAC | ATGGTTTTCAACGACATAGCACGACC |
| 114 | GCC | NNNATAGAACAACCCAACACACACCTTCC | CAGATGGTTTTCAACGACATAGCACG |
| 115 | ATA | NNNGAACAACCCAACACACACCTTCCT | GGCCAGATGGTTTTCAACGACATAG |
| 116 | GAA | NNNCAACCCAACACACACCTTCCTGA | TATGGCCAGATGGTTTTCAACGACATAG |
| 117 | CAA | NNNCCCAACACACACCTTCCTGAGAC | TTCTATGGCCAGATGGTTTTCAACGAC |
| 118 | CCC | NNNAACACACACCTTCCTGAGACGAAG | TTGTTCTATGGCCAGATGGTTTTCAACG |
| 119 | AAC | NNNACACACCTTCCTGAGACGAAGCC | GGGTTGTTCTATGGCCAGATGGTTTT |
| 120 | ACA | NNNCACCTTCCTGAGACGAAGCCTTC | GTTGGGTTGTTCTATGGCCAGATG |
| 121 | CAC | NNNCTTCCTGAGACGAAGCCTTCCC | TGTGTTGGGTTGTTCTATGGCCAGAT |
| 122 | CTT | NNNCCTGAGACGAAGCCTTCCCC | GTGTGTGTTGGGTTGTTCTATGGC |
| 123 | CCT | NNNGAGACGAAGCCTTCCCCATGAC | AAGGTGTGTGTTGGGTTGTTCTATGG |
| 124 | GAG | NNNACGAAGCCTTCCCCATGACTTAAG | AGGAAGGTGTGTGTTGGGTTGTTCTA |
| 125 | ACG | NNNAAGCCTTCCCCATGACTTAAGGC | CTCAGGAAGGTGTGTGTTGGGTT |
| 126 | AAG | NNNCCTTCCCCATGACTTAAGGCGG | CGTCTCAGGAAGGTGTGTGTTGG |
| 127 | CCT | NNNTCCCCATGACTTAAGGCGGCC | CTTCGTCTCAGGAAGGTGTGTGTT |
| 128 | TCC | NNNCCATGACTTAAGGCGGCCGC | AGGCTTCGTCTCAGGAAGGTGTG |
| 129 | CCA | NNNTGACTTAAGGCGGCCGCCGA | GGAAGGCTTCGTCTCAGGAAGG |

## Table S3: Primers used for each Illumina library prepared in this study

| **Library** | **Samples** | **Replicates** | **Description** | **Primers forward** | **Primer reverse** |
| --- | --- | --- | --- | --- | --- |
| 6014-AM | 4 | 1 | KCNE1 trafficking (with KCNQ1) replicate 1 | am10-am13 | am22-25 |
| 6436-AM | 8 | 2 | KCNE1 trafficking (with KCNQ1) replicates 2+3 | am10-am15, am30, am120 | am20-25, am125, am126 |
| 8844-AG | 12 | 3 | KCNE1 trafficking (no KCNQ1) | ag1591-ag1602 | ag1609-1616, ag1603-1606, |
| 9103-AG | 9 | 3 | KCNE1 selection | ag1591-ag1599 | ag1609-1616, ag1603 |
| 4564-AM | 6 | * | Subassembly Library 1 | am110 | am111-am116 |
| 5013-AM | 3 | * | Subassembly Library 2 | am110 | am111-am116 |
| 7207-AM | 3 | * | Subassembly Library 3 | am110c | am208-am210 |

## Table S4: List of presumed pathogenic and presumed benign variants included in the population level analyses

| Variant Name | ClinVar Classifications | Presumed category |
| --- | --- | --- |
| D91E | VUS | Presumed Pathogenic |
| F53S | NotProvided |  |
| F54V | NotProvided |  |
| G52R | P |  |
| H73Y | LP |  |
| I82F | VUS |  |
| K69E | VUS |  |
| K70M | NotProvided |  |
| K70N | NotProvided |  |
| L16P | NotProvided |  |
| L51H | NotProvided |  |
| L59P | VUS |  |
| P127T | NotProvided |  |
| R67L | VUS |  |
| S74L | VUS |  |
| T20I | NotProvided |  |
| T58P | VUS |  |
| T7I | P |  |
| V47F | NotProvided |  |
| W87R | NotProvided |  |
| Y46C | Conf |  |
| Y81C | VUS |  |
| A8V | VUS | Presumed Benign |
| D85N | Conf |  |
| E83K | VUS |  |
| E89K | LB |  |
| G38D | LB |  |
| G38N | LB |  |
| G38S | B |  |
| G40S | LB |  |
| K69R | Conf |  |
| L3P | LB |  |
| R36C | VUS |  |
| R36H | Conf |  |
| R98Q | VUS |  |
| S105L | Conf |  |
| T10M | Conf |  |
| T125M | Conf |  |
| V109I | Conf |  |
| V47I | B |  |
| V80I | VUS |  |

## Table S5: Number of variants in each trafficking category

| **Trafficking**  **category** | **Trafficking score**  **point estimate** | **Trafficking score**  **confidence interval** | **Number of**  **total variants** | **Number of**  **missense variants** |
| --- | --- | --- | --- | --- |
| Loss | <0.20 | N/A | 154 | 105 |
| Partial loss | 0.20-0.83 | CI < 0.83 | 388 | 365 |
| Possible loss | Near 0.83 | CI overlaps 0.83 | 581 | 533 |
| Normal | 0.83-1.18 | 0.83 ≤ CI ≤ 1.18 | 655 | 576 |
| Possible gain | Near 1.18 | CI overlaps 1.18 | 442 | 428 |
| Gain | >1.18 | CI > 1.18 | 334 | 310 |
| All | N/A | N/A | 2554 | 2317 |

## Table S6: Expression levels of *KCNQ1* and *KCNE1* by RNAseq

| **Sample** | ***KCNQ1* variant** | ***KCNE1* variant** | **RNAseq**  **replicates** | ***KCNQ1* FPKM** | ***KCNE1* FPKM** |
| --- | --- | --- | --- | --- | --- |
| Cell line 1 | Wildtype | Wildtype (HA) | 3 | 629.1 ± 5.6 | 82.6 ± 2.5 |
| Cell line 2 | S140G | Wildtype (HA) | 3 | 1065.3 ± 39.9 | 46.4 ± 4.2 |

## Table S7: Number of variants in each functional score category

| **Functional**  **category** | **Functional score**  **point estimate** | **Functional score**  **confidence interval** | **Number of**  **total variants** | **Number of**  **missense variants** |
| --- | --- | --- | --- | --- |
| Loss | <0.09 | N/A | 252 | 173 |
| Partial loss | 0.09-0.44 | CI < 0.44 | 426 | 401 |
| Possible loss | Near 0.44 | CI overlaps 0.44 | 139 | 135 |
| Normal | 0.44-1.53 | 0.44 ≤ CI ≤ 1.53 | 1459 | 1363 |
| Possible gain | Near 1.53 | CI overlaps 1.53 | 262 | 247 |
| Gain | >1.53 | CI > 1.53 | 1 | 1 |
| All | N/A | N/A | 2539 | 2320 |

## Table S8: Relationship between trafficking and functional score categories for missense variants

|  | **Functional category** | | | | | | |
| --- | --- | --- | --- | --- | --- | --- | --- |
| **Trafficking**  **category** | **Loss** | **Partial loss** | **Possible loss** | **Normal** | **Possible gain** | **Gain** | **Any** |
| **Loss** | 64 | 16 | 4 | 19 | 2 | 0 | 105 |
| **Partial loss** | 20 | 52 | 31 | 233 | 29 | 0 | 365 |
| **Possible loss** | 12 | 68 | 34 | 359 | 59 | 1 | 533 |
| **Normal** | 19 | 100 | 28 | 363 | 66 | 0 | 576 |
| **Possible gain** | 16 | 72 | 20 | 256 | 64 | 0 | 428 |
| **Gain** | 42 | 93 | 18 | 130 | 27 | 0 | 310 |
| **Any** | 173 | 401 | 135 | 1360 | 247 | 1 | 2317 |

Missense variants with both measured trafficking and functional scores are shown. There was a statistically significant relationship between these categories (p<2.2X10**^-16^**, Chi square test). The gain-of-function category was removed from the statistical test due to low counts.

## Table S9: Patch clamp data generated in this study

| **Variant** | **Peak-20 (pA/pF)** | **Peak0 (pA/pF)** | **Peak20 (pA/pF)** | **Patch System** | **V_1/2_act (Mut-WT)** | **Peak summary (% of WT)** |
| --- | --- | --- | --- | --- | --- | --- |
| WT | 15.89 | 48.56 | 92.1 | HEK | 0 | 100 |
| I61X | 0 | 0.03 | 0.01 | HEK | * | 0.010857763 |
| G25V | 34.78 | 87.4 | 156.1 | HEK | -18.3 | 169.4896851 |
| G60D | 28.49 | 71.5 | 114.9 | HEK | -1.9 | 124.7557003 |
| D76N | 0.18 | 3.19 | 10.1 | HEK | -13.9 | 10.96634093 |
| G55X | 0.18 | 0.19 | 0.2 | HEK | * | 0.217155266 |
| F57Y | 2.54 | 14.86 | 48.78 | HEK | -1.9 | 52.96416938 |
| R98W | 4.73 | 15.26 | 31.72 | HEK | -12.8 | 34.44082519 |
| D85N | 32.3 | 117.22 | 215.59 | CHO | -20.09 | 234.082519 |
| F53X | 10.03 | 41.94 | 91.23 | CHO | 1.5 | 99.05537459 |
| L3P | 4.8 | 30.3 | 79.9 | CHO | 2.6 | 86.75352877 |
| E19D | 6.69 | 25.07 | 52.15 | HEK | -14.7 | 56.62323561 |
| D39G | 43.9 | 112.5 | 185.1 | HEK | -27.9 | 200.9771987 |
| E101V | 9.08 | 22.39 | 33.38 | HEK | -13.2 | 36.2432139 |
| T125M | 2.99 | 9.36 | 20.43 | HEK | -12.1 | 22.18241042 |
| P127T | 4.8 | 13.1 | 22.7 | CHO | * | 24.64712269 |
| R32T | 7.9 | 36.0 | 78.3 | HEK | -0.1 | 84.989194 |
| Y107R | 64 | 140 | 348 | HEK | -29.4 | 158.18181818 |
| C106L | 52 | 125 | 320 | HEK | -30.0 | 145.45454545 |

## Table S10: List of previously developed computational predictors compared to MAVE scores in this study

| **Name of Metric** | **Reference** | **Year published** |
| --- | --- | --- |
| AlphaMissense | Cheng et al. | 2023 |
| SIFT_score | Ng and Henikoff | 2003 |
| Polyphen2_HVAR_score | Adzhubei et al. | 2010 |
| LRT_score | Chun and Fay | 2009 |
| MutationTaster_score (v2) | Schwarz et al. | 2014 |
| MutationAssessor_score | Reva et al. | 2011 |
| FATHMM_score (weighted) | Shihab et al. | 2013 |
| PROVEAN_score | Choi and Chan | 2015 |
| VEST4_score | Carter et al. | 2013 |
| MetaSVM_score | Dong et al. | 2015 |
| MetaLR_score | Dong et al. | 2015 |
| REVEL_score | Ioannidis et al. | 2016 |
| MutPred_score (v1.2) | Li et al. | 2009 |
| MVP_score | Qi et al. | 2018 |
| MPC_score | Samocha et al. | 2017 |
| CADD_raw | Kircher et al. | 2014 |
| GERP++_RS | Davydov et al. | 2010 |
| phyloP100way_vertebrate | Pollard et al. | 2010 |
| phastCons100way_vertebrate | Siepel et al. | 2005 |

# References

1. Li, H. A statistical framework for SNP calling, mutation discovery, association mapping and population genetical parameter estimation from sequencing data. *Bioinformatics* **27**, 2987–2993 (2011).

2. Li, H. *et al.* The Sequence Alignment/Map format and SAMtools. *Bioinformatics* **25**, 2078–2079 (2009).

3. Warnes, G. R. *et al.* gplots: Various R programming tools for plotting data. *R package version* **2**, 1 (2015).

4. Michael, C. *plotROC: Generate Useful ROC Curve Charts for Print and Interactive Use*. (2015).

5. Roberts, J. D. *et al.* An International Multicenter Evaluation of Type 5 Long QT Syndrome: A Low Penetrant Primary Arrhythmic Condition. *Circulation* **141**, 429–439 (2020).

6. Ávalos Prado, P. *et al.* KCNE1 is an auxiliary subunit of two distinct ion channel superfamilies. *Cell* **184**, 534-544.e11 (2021).

7. Young, W. J. *et al.* Genetic analyses of the electrocardiographic QT interval and its components identify additional loci and pathways. *Nat. Commun.* **13**, 5144 (2022).

8. Lin, Z. *et al.* Evolutionary-scale prediction of atomic level protein structure with a language model. *bioRxiv* 2022.07.20.500902 (2022) doi:10.1101/2022.07.20.500902.

9. Du, Z. *et al.* The trRosetta server for fast and accurate protein structure prediction. *Nat. Protoc.* **16**, 5634–5651 (2021).

10. Jumper, J. *et al.* Highly accurate protein structure prediction with AlphaFold. *Nature* **596**, 583–589 (2021).

11. Mirdita, M. *et al.* ColabFold: making protein folding accessible to all. *Nat. Methods* **19**, 679–682 (2022).

12. Evans, R. *et al.* Protein complex prediction with AlphaFold-Multimer. *bioRxiv* 2021.10.04.463034 (2022) doi:10.1101/2021.10.04.463034.

13. Bisong, E. Google Colaboratory. in *Building Machine Learning and Deep Learning Models on Google Cloud Platform* 59–64 (Apress, 2019).

14. Kang, C. *et al.* Structure of KCNE1 and implications for how it modulates the KCNQ1 potassium channel. *Biochemistry* **47**, 7999–8006 (2008).

15. Gray, J. J. *et al.* Protein-protein docking with simultaneous optimization of rigid-body displacement and side-chain conformations. *J. Mol. Biol.* **331**, 281–299 (2003).

16. Leaver-Fay, A. *et al.* ROSETTA3: an object-oriented software suite for the simulation and design of macromolecules. *Methods Enzymol.* **487**, 545–574 (2011).

17. Barth, P., Schonbrun, J. & Baker, D. Toward high-resolution prediction and design of transmembrane helical protein structures. *Proc. Natl. Acad. Sci. U. S. A.* **104**, 15682–15687 (2007).

18. Sun, J. & MacKinnon, R. Structural basis of human KCNQ1 modulation and gating. *Cell* **180**, 340-347.e9 (2020).

19. Ng, P. C. & Henikoff, S. SIFT: Predicting amino acid changes that affect protein function. *Nucleic Acids Res.* **31**, 3812–3814 (2003).

20. Adzhubei, I. A. *et al.* A method and server for predicting damaging missense mutations. *Nat. Methods* **7**, 248–249 (2010).

21. Chun, S. & Fay, J. C. Identification of deleterious mutations within three human genomes. *Genome Res.* **19**, 1553–1561 (2009).

22. Schwarz, J. M., Cooper, D. N., Schuelke, M. & Seelow, D. MutationTaster2: mutation prediction for the deep-sequencing age. *Nat. Methods* **11**, 361–362 (2014).

23. Reva, B., Antipin, Y. & Sander, C. Predicting the functional impact of protein mutations: application to cancer genomics. *Nucleic Acids Res.* **39**, e118 (2011).

24. Shihab, H. A. *et al.* Predicting the functional, molecular, and phenotypic consequences of amino acid substitutions using hidden Markov models. *Hum. Mutat.* **34**, 57–65 (2013).

25. Choi, Y. & Chan, A. P. PROVEAN web server: a tool to predict the functional effect of amino acid substitutions and indels. *Bioinformatics* **31**, 2745–2747 (2015).

26. Carter, H., Douville, C., Stenson, P. D., Cooper, D. N. & Karchin, R. Identifying Mendelian disease genes with the variant effect scoring tool. *BMC Genomics* **14 Suppl 3**, S3 (2013).

27. Dong, C. *et al.* Comparison and integration of deleteriousness prediction methods for nonsynonymous SNVs in whole exome sequencing studies. *Hum. Mol. Genet.* **24**, 2125–2137 (2015).

28. Ioannidis, N. M. *et al.* REVEL: An ensemble method for predicting the pathogenicity of rare missense variants. *Am. J. Hum. Genet.* **99**, 877–885 (2016).

29. Samocha, K. E. *et al.* Regional missense constraint improves variant deleteriousness prediction. *bioRxiv* 148353 (2017) doi:10.1101/148353.

30. Kircher, M. *et al.* A general framework for estimating the relative pathogenicity of human genetic variants. *Nat. Genet.* **46**, 310–315 (2014).

31. Davydov, E. V. *et al.* Identifying a high fraction of the human genome to be under selective constraint using GERP++. *PLoS Comput. Biol.* **6**, e1001025 (2010).

32. Pollard, K. S., Hubisz, M. J., Rosenbloom, K. R. & Siepel, A. Detection of nonneutral substitution rates on mammalian phylogenies. *Genome Res.* **20**, 110–121 (2010).

33. Siepel, A. *et al.* Evolutionarily conserved elements in vertebrate, insect, worm, and yeast genomes. *Genome Res.* **15**, 1034–1050 (2005).

34. Li, B. *et al.* Automated inference of molecular mechanisms of disease from amino acid substitutions. *Bioinformatics* **25**, 2744–2750 (2009).

35. Cheng, J. *et al.* Accurate proteome-wide missense variant effect prediction with AlphaMissense. *Science* **381**, eadg7492 (2023).

36. Liu, X. & Jian, X. dbNSFP: a lightweight database of human non-synonymous SNPs and their functional predictions. *Human Mutation* **32**, 894–899 (2011).

37. Liu, X., Li, C., Mou, C., Dong, Y. & Tu, Y. dbNSFP v4: a comprehensive database of transcript-specific functional predictions and annotations for human nonsynonymous and splice-site SNVs. *Genome Med.* **12**, 103 (2020).

38. Chen, S., Zhou, Y., Chen, Y. & Gu, J. fastp: an ultra-fast all-in-one FASTQ preprocessor. *Bioinformatics* **34**, i884–i890 (2018).

39. Kim, D., Paggi, J. M., Park, C., Bennett, C. & Salzberg, S. L. Graph-based genome alignment and genotyping with HISAT2 and HISAT-genotype. *Nat. Biotechnol.* **37**, 907–915 (2019).

40. Wagner, J. *et al.* Curated variation benchmarks for challenging medically relevant autosomal genes. *Nat. Biotechnol.* **40**, 672–680 (2022).

41. Pertea, M. *et al.* StringTie enables improved reconstruction of a transcriptome from RNA-seq reads. *Nat. Biotechnol.* **33**, 290–295 (2015).

42. Pertea, M., Kim, D., Pertea, G. M., Leek, J. T. & Salzberg, S. L. Transcript-level expression analysis of RNA-seq experiments with HISAT, StringTie and Ballgown. *Nat. Protoc.* **11**, 1650–1667 (2016).

43. Wang, K. W. & Goldstein, S. A. Subunit composition of minK potassium channels. *Neuron* **14**, 1303–1309 (1995).
